# Maize specialized metabolome networks reveal organ-preferential mixed glycosides

**DOI:** 10.1016/j.csbj.2021.01.004

**Published:** 2021-01-26

**Authors:** Sandrien Desmet, Yvan Saeys, Kevin Verstaen, Rebecca Dauwe, Hoon Kim, Claudiu Niculaes, Atsushi Fukushima, Geert Goeminne, Ruben Vanholme, John Ralph, Wout Boerjan, Kris Morreel

**Affiliations:** a Department of Plant Biotechnology and Bioinformatics, Ghent University, B-9052 Gent, Belgium; b Center for Plant Systems Biology, VIB, B-9052 Gent, Belgium; c Department of Applied Mathematics, Computer Science and Statistics, Ghent University, B-9052 Gent, Belgium; d Data Mining and Modelling for Biomedicine, Center for Inflammation Research, VIB, B-9052 Gent, Belgium; e Unité de Recherche BIOPI EA3900, Université de Picardie Jules Verne, 80000 Amiens, France; f Department of Biochemistry and the U.S. Department of Energy Great Lakes Bioenergy Research Center, Wisconsin Energy Institute, University of Wisconsin, Madison, WI 53726, United States; g Plant Breeding, TUM School of Life Sciences Weihenstephan, Technical University of Munich, 85354 Freising, Germany; h RIKEN Center for Sustainable Resource Science, Yokohama, Kanagawa 230-0045, Japan; i VIB Metabolomics Core Ghent, VIB, B-9052 Gent, Belgium

**Keywords:** Mass spectrometry, Specialized metabolism, Spectral metadata analysis, Zea mays

## Abstract

Despite the scientific and economic importance of maize, little is known about its specialized metabolism. Here, five maize organs were profiled using different reversed-phase liquid chromatography-mass spectrometry methods. The resulting spectral metadata, combined with candidate substrate-product pair (CSPP) networks, allowed the structural characterization of 427 of the 5,420 profiled compounds, including phenylpropanoids, flavonoids, benzoxazinoids, and auxin-related compounds, among others. Only 75 of the 427 compounds were already described in maize. Analysis of the CSPP networks showed that phenylpropanoids are present in all organs, whereas other metabolic classes are rather organ-enriched. Frequently occurring CSPP mass differences often corresponded with glycosyl- and acyltransferase reactions. The interplay of glycosylations and acylations yields a wide variety of mixed glycosides, bearing substructures corresponding to the different biochemical classes. For example, in the tassel, many phenylpropanoid and flavonoid-bearing glycosides also contain auxin-derived moieties. The characterized compounds and mass differences are an important step forward in metabolic pathway discovery and systems biology research. The spectral metadata of the 5,420 compounds is publicly available (DynLib spectral database, https://bioit3.irc.ugent.be/dynlib/).

## Graphical abstract

**Figure:**
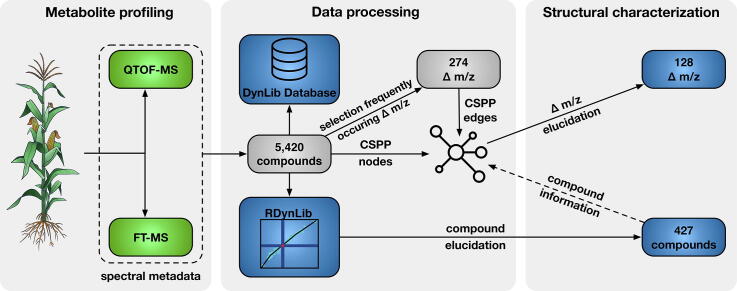

## 1 Introduction

Maize (*Zea mays L.* ssp. *Mays*) is widely known for its importance as a food and feed crop, but it is also a predominant feedstock for the production of renewable chemicals and fuels, such as bio-ethanol [1], [2], [3], [4]. In addition, maize is of substantial scientific importance; the availability of a vast collection of mutants, the high degree of genomic collinearity with other cereal crops and related grasses, and the availability of the genome sequence together with the extensive nucleotide diversity, have made maize a model system for basic and applied research [5], [6], [7], [8].

Current research focuses mainly on deciphering the maize genome by functionally annotating the numerous unknown genes [9], [10], [11]. This functional genomics approach has been advanced considerably by the integration of metabolomics [12]. The metabolome, i.e., the complete set of metabolites in an organism, provides a phenotypic read-out, which can yield insight into how genes, transcripts, proteins and metabolites drive and influence the phenotype of a system. This property makes metabolomics an essential player in the understanding of cellular systems and in decoding the function of genes [13]. Nonetheless, metabolome data and knowledge of metabolite identities remain scarce for maize, especially concerning its specialized (secondary) metabolism. This information gap not only prevents understanding the system-wide biology of maize, but also the continuous development of maize as a model system.

The lack of metabolite identities also highly contrasts with the importance of specialized metabolites in various biological processes; they are involved in the attraction of pollinators, in the interaction of the plant with its environment (e.g., microorganisms in the soil), in nutrient uptake, and in plant defense against biotic (e.g., herbivores and pathogens) and abiotic (e.g., UV-radiation) stresses. Furthermore, many specialized metabolites possess biological activity [14], [15], [16], which is exploited in the pharmaceutical industry. Indeed, many drugs or precursors for drug synthesis are derived from plant specialized metabolites [17].

The main reason for the low number of known metabolites is the tremendous effort needed to identify the structures of new metabolites, which is either done via purification followed by nuclear magnetic resonance (NMR) analysis, or via authentication based on chemically synthesized standards and subsequent analysis by mass spectrometry (MS). In MS-based metabolite profiling, structural information on an unknown metabolite is gained via its collision-induced dissociation (CID)-spectrum, which can be matched against spectral databases [18], [19]. However, spectral matching typically yields very few annotations, because these databases cover only a small fraction of all metabolites in plants [20]. This shortcoming has led to the development of various *de novo* structural elucidation programs [21], [22], [23], [24], [25], [26], [27], [28]. Spectral databases are quickly emerging [29], but tend to focus on certain types of CID spectra, consequently restricting spectral matching and structural prediction to those spectral databases and *de novo* elucidation programs that can handle the particular CID spectral type. There are two types of CID spectra that are primarily employed in liquid chromatography (LC)-MS [30]: tandem-in-space MS/MS spectra, generated in, e.g., quadrupole-time-of-flight (QTOF) MS instruments, and tandem-in-time MS^n^ spectra, generated in, e.g., ion trap (IT) MS instruments. Both CID spectra provide complementary information on the structure of the unknown compound, which is advantageous for spectral database matching, spectral interpretation using chemical principles, and automated, often machine-learning-based, structural elucidation. Ideally, samples have to be analyzed several times, on different instruments and using different settings to collect, for each compound, an extensive set of mass spectral data, referred to as spectral metadata hereafter. However, prior to the use of spectral metadata for structural characterization, the multiple MS/MS and MS^n^ data for each profiled compound have to be associated. This requires the development of a spectral database in combination with an alignment tool.

**Fig. 1:**
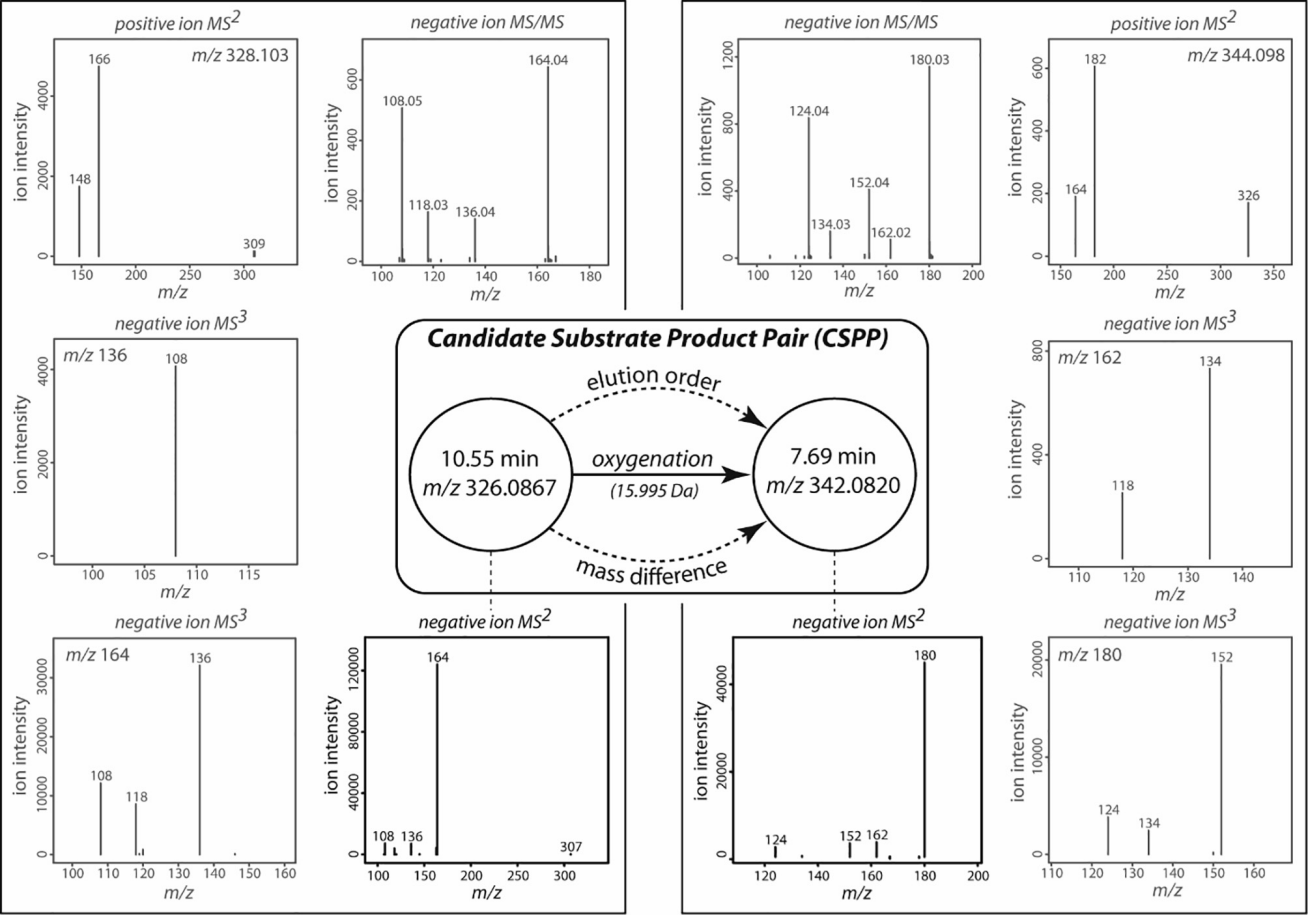
Combined Candidate Substrate–Product Pair (CSPP) / Spectral Meta Data Analysis. A CSPP is defined whenever two features have a mass difference corresponding to a biotransformation (e.g., a difference of 15.995 Da is expected in the case of an oxygenation) and an elution order that agrees with the expected change in molecular structure (e.g., the compound representing the “product” feature is expected to elute earlier than that representing the “substrate” feature on a reverse-phase column in the case of an oxygenation). In Morreel *et al*. (2014) [35], further support that the CSPP reflects a biochemical conversion had been obtained by considering the similarity between the negative ion MS^2^ spectra (black) of the CSPP “substrate” and “product” features. In this study, both positive and negative ionization MS^n^ and MS/MS spectra (gray), were associated with the CSPP.

In addition to the progress made in CID spectral analysis, MS spectral interpretation has also been improved by considering biotransformations. Because a limited number of organic reactions represent most of the enzymatic reactions, mass differences corresponding to those organic reactions, for example, 14.015 Da in the case of a methylation, can be searched for between pairs of features [peaks that are defined by a retention time and a mass-to-charge (*m*/*z*) value]. Biotransformations were first taken into account when interpreting direct infusion Fourier-transform (FT) MS spectra [31], [32]. This method has been further developed for use with LC-MS data [33], [34], and extended by including the elution order between the candidate “substrate” and candidate “product” features (Fig. 1) [35]. Concatenating these candidate “substrate–product” feature pairs into a candidate substrate–product pair (CSPP) network with nodes and edges representing features and biotransformations, respectively, significantly advanced structural characterization via a propagation approach starting from known network nodes [35], [36]. Associating multiple CID spectra, e.g., MS/MS and MS^n^ spectra, with each CSPP network node, provides complementary structural information (Fig. 1) to further boost the CSPP-based structural elucidation pipeline. In addition, the CSPP-based structural elucidation pipeline would benefit from including as many pathways and their intermediates as possible. Because different classes of specialized metabolites often accumulate in specific plant organs [37], [38], CSPP network propagation would be improved by the analysis of different organs in order to maximize the variety of profiled specialized metabolites.

In this research, we focused on the phenolic metabolism of maize. Five maize organs and four genotypes were profiled using different reversed-phase LC-MS methods. All CID spectra were archived in a spectral database called DynLib, and are publicly available via an online webtool (https://bioit3.irc.ugent.be/dynlib/). The CID spectra associated with the same compound were linked using a newly developed R package, called RDynLib. This package also allows the visualization of spectral metadata and local CSPP networks for each compound. Using the various tools implemented in RDynLib, 427 compounds were structurally elucidated, of which 200 were at least partially authenticated via profiling of purchased compounds. Remarkable in this compound set was the rich variety of auxin glycosides in the tassel and corn cob, most of which had not been described before. Using the set of characterized compounds, the most frequently occurring mass differences within the maize metabolite profiles were determined and characterized. Acylations and glycosylations were among the most frequently observed biotransformations in the CSPP network, yielding a wide variety of glycosylated molecules bearing moieties corresponding to different metabolic classes.

The combination of the characterized compounds and mass differences are an important step forward in metabolic pathway discovery in maize, and the study of the specialized metabolism in general.

## 2 Methods

### 2.1 Growth, harvest and metabolite extraction conditions

From a maize field plot planted in May 2017 at the ILVO fields in Wetteren (Belgium), ears of the genotype CML91, H99, W153R, and OH43 were harvested at the end of June. Late cobs, leaves, stems (internodium that bears the maize cob), and tassels from these four genotypes were harvested at the end of August. The 20 samples (four genotypes and five organ types, no biological replicates were included) were separately homogenized using a GRINDOMIX GM 200 (Retch GmbH, Germany). Approximately 200 mg fresh weight was extracted with 1 mL methanol. Following evaporation of the methanol supernatant, an extraction with 0.8 mL of Milli-Q water/cyclohexane (1/1, v/v) was performed as previously described [39]. Finally, 0.2 mL of the aqueous phase was stored at −80 °C.

### 2.2 LC-MS profiling

Each metabolic extract originating from the maize field plot (10 μL injected) was profiled in negative and positive ionization mode using two mass spectrometers, a UHPLC-ESI-QTOF-MS (Acquity UPLC system coupled to a Synapt High Definition MS, Waters Corporation, Manchester, UK) and a UHPLC-IT-FT-ICR-MS (Accela UHPLC system coupled to an LTQ FT Ultra, Thermo Scientific, Bremen, Germany). On both instrument platforms, a reversed-phase separation was performed using an Acquity UPLC BEH C18 (2.1 × 150 mm, 1.7 μm; Waters Corporation) column heated to 40 °C. The mobile phase was gradually changed from 99% solvent A (99/1/0.1 Milli-Q water/acetonitrile/formic acid, v/v/v) to 50% solvent B (99/1/0.1 acetonitrile/Milli-Q water/formic acid, v/v/v) in 30 min using a flow of 350 μL/min. On the FT platform, full FT-ICR-MS scans between *m*/*z* 100 and *m*/*z* 1000 were recorded in parallel with data-dependent IT-MS^n^ scans (35% collision energy) consisting of one MS^2^ scan and three MS^3^ scans. For each ionization mode and each sample, two runs were performed, recording MS^3^ spectra of the 1st, 2nd and 3rd and of the 3rd, 4th and 5th most abundant MS^2^ product ions, respectively. The ESI source voltage, capillary voltage, tube lens, capillary temperature, sheath gas, and aux gas were set at −4.5 kV, −18 V, −150 V, 275 °C, 20 (arb) and 5 (arb) and 4 kV, 1 V, 40 V, 275 °C, 8 (arb) and 0 (arb) in negative and positive ionization mode. On the QTOF platform, two runs per ionization mode and per sample were performed, recording full MS data between *m*/*z* 100 and *m*/*z* 1000 in the first run, and recording data-dependent analysis-based MS/MS spectra for a maximum of three ions for prominent masses selected from a single MS survey scan in the second run. The capillary voltage, sampling cone and extraction cone were set at −2.5 kV, −37 V and −3.5 V and 2.5 kV, 40 V and 3.5 V in negative and positive ionization mode. In both ionization modes, the source and desolvation temperatures were 120 and 400 °C. The cone and desolvation gas flows were set at 50 and 550 L/h and 50 and 500 L/h in the case of negative and positive ionization mode. The trap and transfer collision energies were 4 and 3 V, and 6 and 4 V for negative and positive ionization. For data-dependent analysis, a ramping between 10 and 20 eV and between 20 and 45 eV was applied for the low and high mass ions.

### 2.3 Data processing, database and RDynLib construction, and structural elucidation

The processing of the LC-MS data is described in the Supplemental Text (*LC-MS data processing*). Also the construction of the DynLib database (*DynLib database construction*), the development and application of the RDynLib package (*RDynLib construction and application*), and the structural elucidation of the CID spectra are described in the Supplemental Text (*Structural elucidation of CID spectra*; Supplemental Fig. 10 and 14–23). The maize DynLib database csv files, the perl scripts to upload CID spectra into the DynLib database, the RDynLib package, a file explaining the different functions in RDynLib (‘RDynLib tools’), and two pptx files explaining how to upload data into the DynLib database via RDynLib and how to elucidate CID spectra via RDynLib, are available at https://floppy.psb.ugent.be/index.php/s/O9z6mU8IiAlWGbT. The DynLib database webtool can be consulted at https://bioit3.irc.ugent.be/dynlib/.

### 2.4 CSPP network construction and data analyses

The construction of Manhattan plots displaying the number of feature pairs versus the mass difference, selection of the prevailing mass differences, and construction of CSPP networks, were performed as described previously [35]. All multivariate data analyses occurred in R version 3.4.2 [40]. PCA analysis [*PCA(data, graph = F)* function, FactoMineR package [41]] was performed using either feature abundances or mass difference frequencies. In the case of mass differences, proportional data were obtained by dividing each mass difference by its frequency threshold computed following the approach described in Morreel (2014) [35]. Both feature abundances and mass difference frequencies were centered and unit variance-scaled. PCA results were visualized using the *fviz_pca_ind(PCA, col.ind=“cos2″)* and *fviz_pca_biplot(PCA, repel = TRUE, select.var = list(contrib = 10))* functions of the FactoExtra package (https://CRAN.R-project.org/package = factoextra). The Venn diagrams were generated using the *venn.diagram()* function in the VennDiagram package [42] in R.

## 3 Results

### 3.1 Adding LC-MS data to the DynLib database

In order to characterize the maize phenolic metabolism, methanol extracts from five different organs (stem internodium, leaf, tassel, ear and late cob) and four genotypes (CML91, H99, W153R and OH43) were profiled via reversed-phase LC-MS using two instrument platforms: (i) an ultra-high-performance liquid chromatography (UHPLC) hyphenated via an electrospray ionization (ESI) source to an ion-trap Fourier-transform ion-cyclotron-resonance mass spectrometer (IT-FT-ICR-MS; hereafter abbreviated simply as FT) and (ii) an UHPLC-ESI-QTOF-MS (hereafter abbreviated simply as QTOF). Negative and positive ionization data were recorded on each platform yielding the FTneg, FTpos, QTOFneg, and QTOFpos sets of raw data. The FT was used to generate MS^n^ spectra (in which each IT-based MS^n^ spectrum represents an MS^2^ spectrum and optionally one or more MS^3^ spectra, each displaying second-order product ions resulting from the fragmentation of a particular, MS^2^-derived, first-order product ion), whereas the QTOF was used to generate MS/MS spectra.

**Fig. 2:**
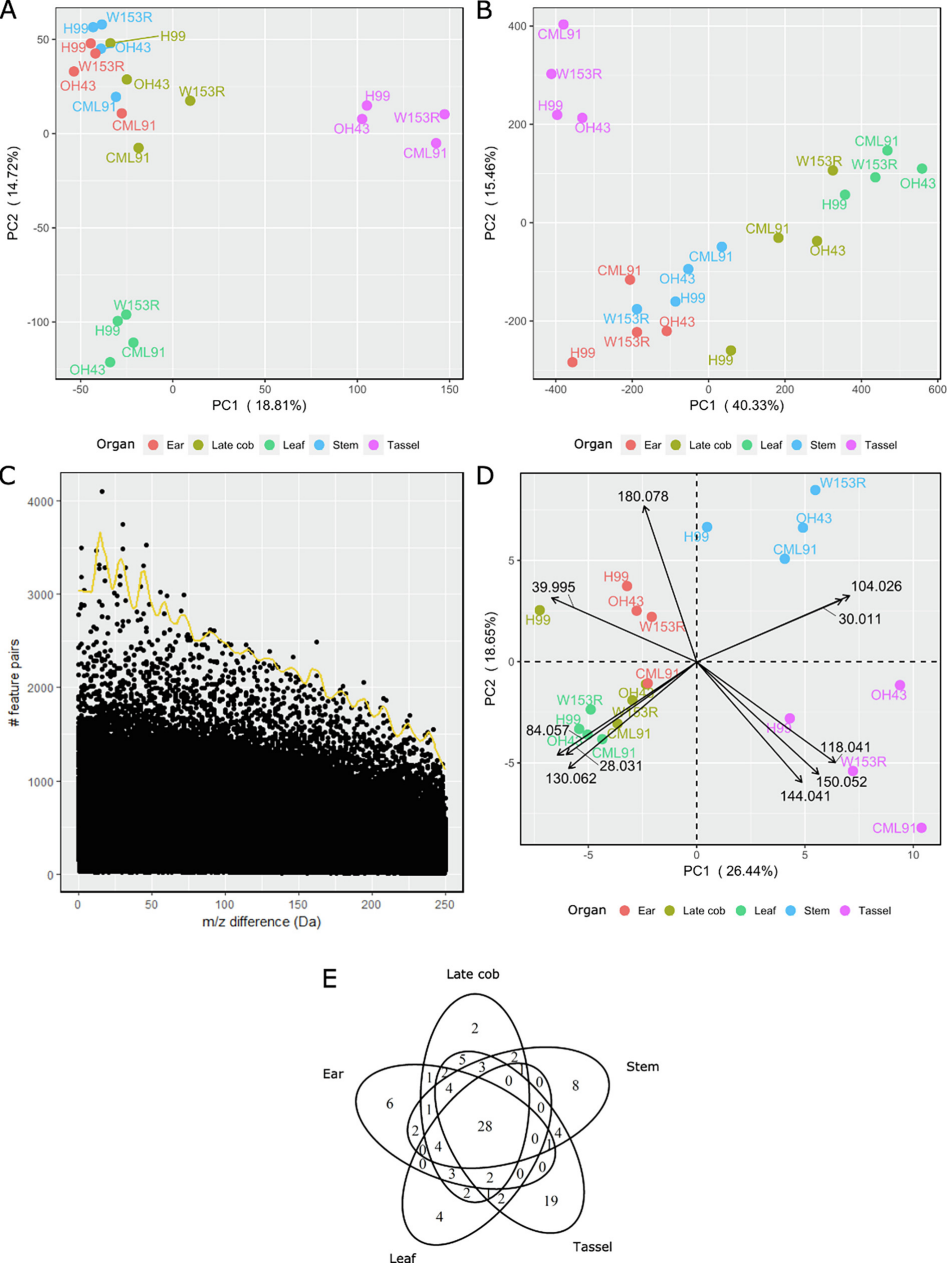
Principal Component Analysis (PCA) and Differential Biotransformation Enrichment. All plots are based on negative ionization UHPLC-FT profiling. (A) PCA plot based on the feature abundances. Data were centered and unit variance-scaled. (B) PCA plot of the number of feature pairs for all 250,000 mass differences (between 0 and 250 Da, with a precision of 0.001 Da). Mass differences were divided by their local frequency threshold following the approach in Morreel *et al*. (2014) [35], and subsequently centered and unit variance-scaled. (C) Manhattan plot showing the number of feature pairs for each mass difference between 0 and 250 Da (0.001 Da interval) based on the stem data from all genotypes. Filtering of frequently occurring mass differences is based on the threshold line shown in yellow (see Methods). (D) Biplot (PCA plot + loading plot) based on the number of feature pairs for the selected mass differences, i.e., biotransformations. The loadings represent the top ten variables (mass differences, see Table 3), contributing to the principal components. (E) Venn diagram of the organ distribution of the biotransformations. Presence/absence of a biotransformation in a particular organ is based on the threshold line displayed in the Manhattan plots (see C and Table 3). PCA plots display the principal component 1 (PC1) and 2 (PC2) values. The variances explained by PC1 and PC2 are indicated between parentheses. (For interpretation of the references to color in this figure legend, the reader is referred to the web version of this article.)

To obtain a general impression of the variation between the profiles, the FT data were subjected to a principal component analysis (PCA) of the feature abundances following chromatogram processing. The PCA yielded three distinct clusters based on the first and second principal components (PC1 and PC2) for the FTneg (Fig. 2A) as well as the FTpos data sets (Supplemental Fig. 1A). The profiles from stem, ear, and late cob clustered together, whereas those of leaf and tassel were present in two distinct clusters. Together, PC1 and PC2 captured 34% (FTneg) and 23% (FTpos) of the variation between metabolite profiles, reflecting differences between the plant organs rather than between the genotypes.

Because different CID spectra provide complementary information on the structure of the unknown compounds, structural characterization would optimally benefit from including all profiling data, i.e., negative and positive ionization spectra and both MS/MS and MS^n^ spectra. To initiate such a strategy, all CID spectra obtained by LC-MS profiling of the different organs of the four maize genotypes were collected in an in-house-generated database (Supplemental Table 1), referred to as the ‘Dynamic Library’ or, simply the DynLib database (Supplemental Fig. 2; an in-depth description is given in Supplemental Text, *DynLib database construction* and *Adding MS spectral data to the DynLib database*). The DynLib database consists of four sub-databases (hereafter referred to as subDBs), i.e., for each instrument platform (FT or QTOF) and for each ionization mode, and these subDBs are called ‘FTMS_neg’, ‘FTMS_pos’, ‘QTOF_neg’, and ‘QTOF_pos’ (names differ slightly from those used for the corresponding raw data sets to stress the distinction between a raw LC-MS data set and a CID spectra-specific subDB). Following CID spectral archiving, the FTMS_neg and FTMS_pos, and the QTOF_neg and QTOF_pos subDBs contained 4,208 and 1,785 MS^n^ spectra, and 2,665 and 1,816 MS/MS spectra, respectively. Taking into account the many LC-MS features (and their associated CID spectra) representing a particular compound, and the presence of the CID spectral information across the four different DynLib subDBs, CID spectral data of approximately 5,420 compounds (see Supplemental Text, *Adding MS spectral data to the DynLib database*) were uploaded into the DynLib database.

In order to exploit the complementarity of the different CID spectra, the features representing the same ions in the different subDBs of the DynLib database have to be aligned. Therefore, an R package called RDynLib was created. Because the alignment occurs between subDBs rather than chromatograms, only features for which CID spectra are available were included in the alignment. The alignment via the RDynLib package is therefore based on a combination of CID spectral matching and retention time alignment (an in-depth description is given in Supplemental Text, *Aligning SubDB experiments using RDynLib*; Supplemental Figs. 3 and 4). This alignment procedure was executed between the FTMS_neg and QTOF_neg, the FTMS_pos and QTOF_pos, the FTMS_neg and the FTMS_pos, and the QTOF_neg and QTOF_pos subDBs and resulted in 843, 287, 542 and 477 aligned features, respectively. Thus, between any pair of subDBs, the number of features that could be aligned ranged between 13% and 32% of the total number of features in each of the two subDBs in the considered alignment (Table 1).

**Table 1:** Number of CID spectra per DynLib subDB.

	DynLib subDB			
	FTMS_neg	FTMS_pos	QTOF_neg	QTOF_pos
# CID spectra	4,208	1,785	2,665	1,816
vs FTMS_neg	4,208 (1.00)	542 (0.30)	843 (0.32)	
vs FTMS_pos	542 (0.13)	1,785 (1.00)		287 (0.16)
vs QTOF_neg	843 (0.20)		2,665 (1.00)	477 (0.26)
vs QTOF_pos		287 (0.16)	477 (0.18)	1,816 (1.00)

Each column represents a DynLib subDB. The first row represents the number of CID spectra (MS^n^ and MS/MS spectra in case of the FTMS-based and the QTOF-based subDB, respectively) in each of the subDB. The cells in the remaining rows display the number of aligned features between the considered subDBs, indicated by the row and column header of each cell. Between parentheses, the proportion is shown of the number of aligned features versus the total number of features in the subDB mentioned in the column header. Feature alignment was not performed between the QTOF_neg and the FTMS_pos, and between the QTOF_pos and the FTMS_neg subDBs.

### 3.2 Added value of including both MS/MS and MS^n^ spectra for structural elucidation

To gain insight into the added value of the aligned MS spectral metadata (MS^n^ and MS/MS spectra) in the DynLib database for structural elucidation, we performed spectral matching of the unique CID spectra with publicly available CID spectral databases (http://mona.fiehnlab.ucdavis.edu/downloads) (see Supplemental Text for details, *Structural elucidation of CID spectra*). The external CID spectral databases that were consulted comprised MassBank [18], ReSpect [43], HMDB [44], GNPS [29], iTree [45] and MetaboBASE (https://sumnerlab.missouri.edu/download/).

Using a spectral similarity threshold of 0.6, 39 of the 4,208 (0.93%), 44 of the 1,785 (2.46%), 58 of the 2,665 (2.18%), and 43 of the 1,816 (2.37%) CID spectra in the FTMS_neg, FTMS_pos, QTOF_neg and QTOF_pos DynLib subDBs, respectively, had a positive match with at least one CID spectrum in the external databases. Focusing on ions having negative ionization MS^2^ as well as negative ionization MS/MS spectra (843 ions), 15 ions (1.78%) had a positive match based on their MS^2^ spectrum and 16 ions (1.90%) had a positive match based on their MS/MS spectrum. Only 3 of the 843 ions (0.36%) had a spectral match with both their MS^2^ and MS/MS spectrum. When focusing on ions having both positive ionization MS^2^ and positive ionization MS/MS spectra (287 ions), 12 (4.18%) ions had a positive match based on their MS^2^ spectra and 7 ions (2.44%) had a positive match based in their MS/MS spectrum. Similar to the results based on the negative ionization mode, only 3 of the 287 ions (1.05%) were matched via both their MS^2^ and MS/MS spectrum. Thus, most of the ions were matched to external CID spectral databases via either their MS^2^ or their MS/MS spectrum, showing the importance of including both types of CID spectra for spectral matching. However, these results also illustrate the very low number of ions from specialized metabolism that can be annotated via spectral matching with publicly available CID spectral databases, highlighting the need for structural characterization tools that take advantage of the information present in LC-MS data and different types of CID spectra.

### 3.3 Characterizing the maize specialized metabolome via RDynLib

Various MS spectral analysis tools are included in RDynLib to facilitate structural characterization and to exploit the spectral metadata (see Supplemental Text, *Structural characterization tools in RDynLib*; Supplemental Figs. 5–10). In addition to these tools, RDynLib allows mass difference analysis via the construction of local CSPP networks (based on a fixed set of 34 well-known biotransformations) (see Supplemental Text, *Structural characterization tools in RDynLib*; Supplemental Fig. 11). Using the MS spectral metadata, the spectral and mass-difference analysis tools in RDynLib, and knowledge about the gas-phase fragmentations for particular compound classes (see Supplemental Text, *Compound class-specific gas-phase fragmentations*), we structurally characterized 427 compounds from the maize-derived CID spectra present in the DynLib database (Supplemental Data Set 1). The structures of 72 compounds were verified via identity matching of the CID spectra with those of purchased standards (see Supplemental Text, *Compound class-specific gas-phase fragmentations*). For another 128 compounds, structural moieties were identified via identity matching of the corresponding MS^3^ spectra with the MS^2^ spectra of purchased standards. When consulting the PubChem database (https://pubchem.ncbi.nlm.nih.gov/), a compound database containing the structures of over 100 million compounds, 168 of the 427 compounds could be found, whereas a structurally highly similar isomer (Tanimoto coefficient > 0.95) was present for another 104 compounds. Based on the FooDB (http://foodb.ca/) and the CornCyc (https://www.plantcyc.org/databases/corncyc/9.0) databases, only 75 of the 427 compounds were already found in maize.

**Fig. 3:**
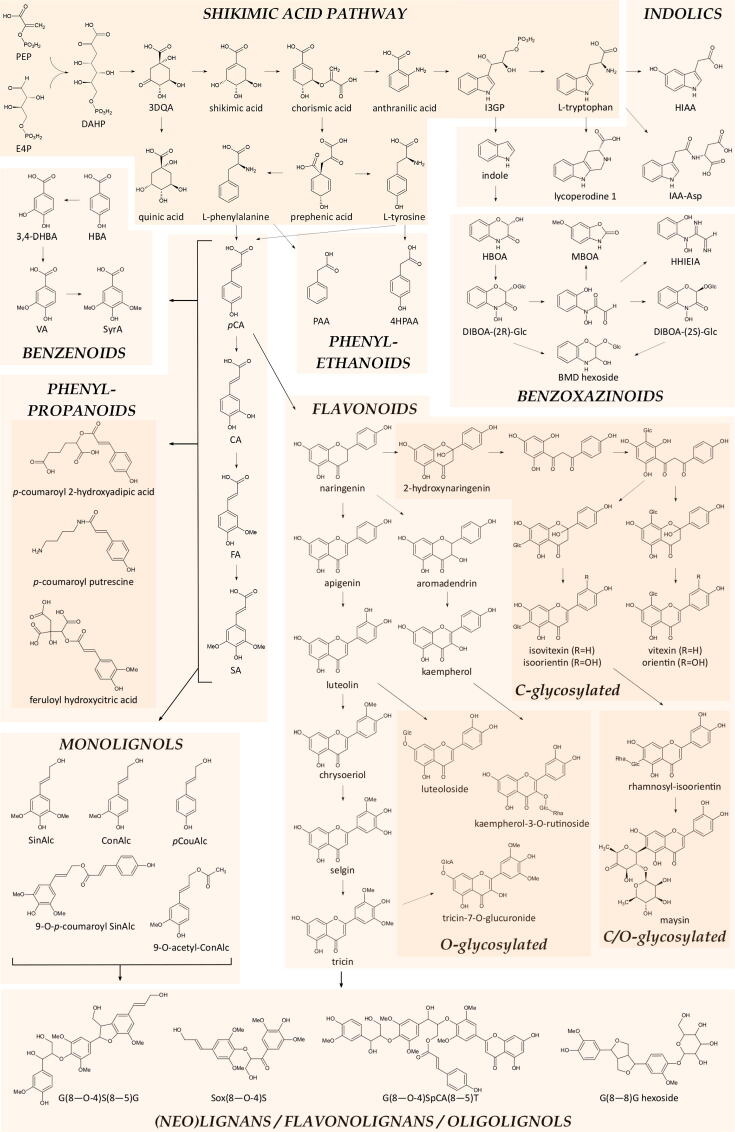
Overview of Shikimic Acid-Derived Metabolic Pathways. Thin arrows represent one or multiple, either well-known or presumed, biochemical conversion(s). Thick arrows indicate one or multiple compound(s) that serve as precursor(s) without specification of a particular biochemical route. (Neo)Lignans, flavonolignans and oligolignols are given descriptive shorthand names following a previously described convention [35]. G, S, T, Sox, and SpCA refer to moieties derived from coniferyl alcohol (yielding the guaiacyl unit), sinapyl alcohol (yielding the syringyl unit), tricin, 7-oxo-sinapyl alcohol, and 9-*O*-*p*-coumaroyl sinapyl alcohol, respectively. The (8–O-4)-, (8–5)- and (8–8)-linkages refer to β-aryl ether, phenylcoumaran and resinol units, respectively. 3DQA 3-dehydroquinic acid; 3,4-DHBA 3,4-dihydroxybenzoic acid; 4HPAA 4-hydroxyphenylacetic acid; BMD benzomorpholine-2,3-diol; CA caffeic acid; ConAlc coniferyl alcohol; DAHP 3-deoxy-D-*arabino*-heptulosonate-7-phosphate; DIBOA-(2R)-Glc 2,4-dihydroxy-2*H*-1,4-benzoxazin-3(4*H*)-one-(2*R*)-β-D-glucoside; DIBOA-(2S)-Glc 2,4-dihydroxy-2*H*-1,4-benzoxazin-3(4*H*)-one-(2*S*)-β-D-glucoside; E4P D-erythrose-4-phosphate; FA ferulic acid; Glc glucose; GlcA glucuronic acid; HBA *p*-hydroxybenzoic acid; HBOA 2-hydroxy-2–1,4-benzoxazin-3-one; HIAA 5-hydroxyindole-3-acetic acid; I3GP indole-3-glycerol phosphate; IAA-Asp indole-3-acetyl-L-aspartic acid; MBOA 6-methoxybenzoxazolinone; PAA phenylacetic acid; *p*CA *p*-coumaric acid; *p*CouAlc *p*-coumaryl alcohol; PEP phosphoenolpyruvic acid; Rha rhamnose; SA sinapic acid; SinAlc sinapyl alcohol; SyrA syringic acid; VA vanillic acid.

**Fig. 4:**
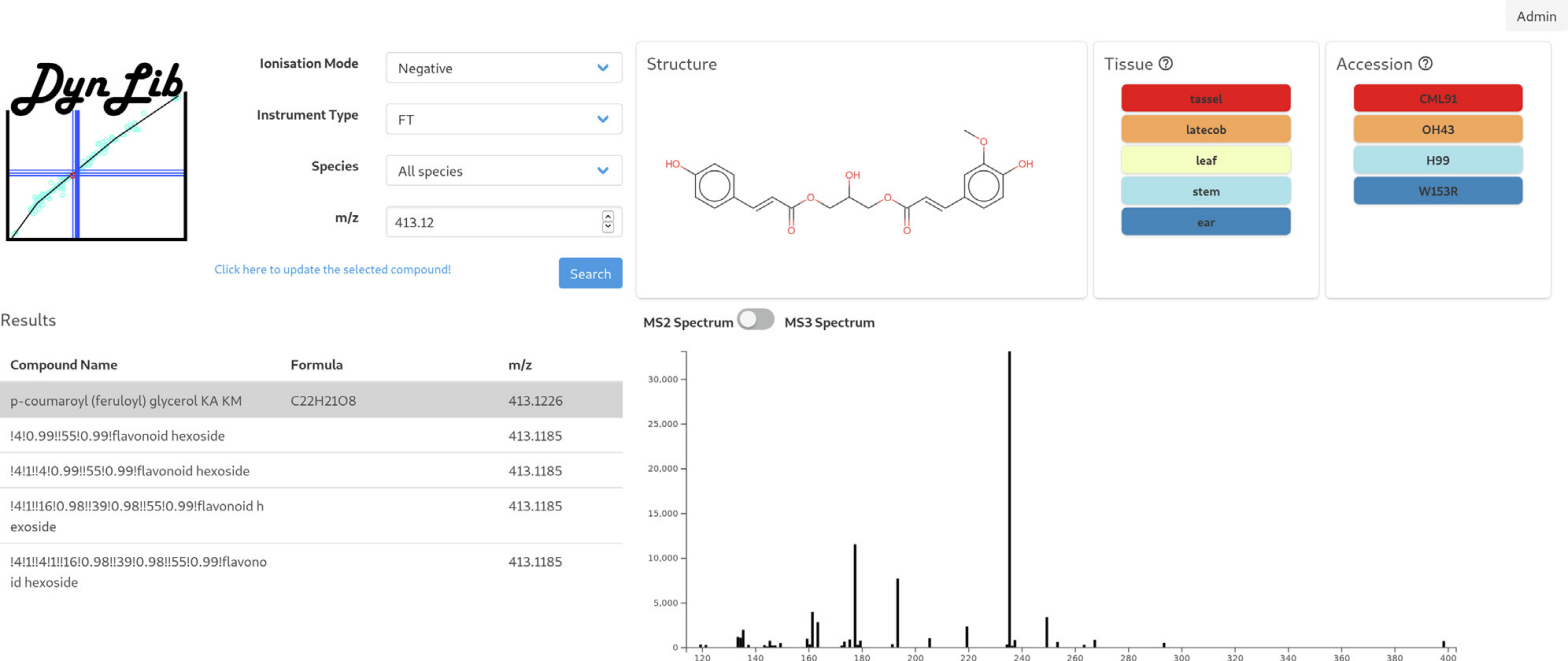
Screenshot of the DynLib Webtool. Based on the entered ionization mode, instrument, species and *m*/*z* value, all CID spectra are returned. Relative abundances (lowest and highest abundances are indicated by blue and red, respectively) of the precursor ion in the five organs and the four genotypes are shown. In case the CID spectrum was elucidated, a trivial name for the compound and its structure are returned. (For interpretation of the references to color in this figure legend, the reader is referred to the web version of this article.)

The number of characterized compounds per chemical class is shown in Table 2. The metabolic classes with the largest number of characterized compounds were the phenylpropanoids and their glycosides, the *O*- and *C*-glycosylated flavonoids and the mixed glycosides with 82, 40, 36 and 77 characterized compounds, respectively. An overview of the shikimic acid-derived metabolic pathways is shown in Fig. 3. The ‘mixed glycoside’ class contained saccharides to which moieties of at least two different chemical classes were attached, hence, preventing them from being included in one of the other chemical classes. For example, many phenylpropanoid and flavonoid-bearing glycosides also contain auxin-derived moieties. Phenylpropanoids were found in all organs, whereas *O*- and *C*-glycosylated flavonoids were mainly present in leaf and tassel, with the *O*-glycosylated flavonoids being abundant in the stem as well. Other specialized metabolic classes that frequently occurred were the flavonolignans (28; enriched in the leaf, stem, and tassel), benzenoids (21; tassel), indolics (22; tassel), and benzoxazinoids (15; ear and leaf). In addition, 14 oligolignols and a number of compounds belonging to other metabolic classes were characterized. The class of oligolignols contained only aglycones; the eight characterized oligolignol glycosides were classified as (neo)lignans. Oligolignols and (neo)lignans were enriched in the stem, and in the stem and leaf, respectively. A webtool (https://bioit3.irc.ugent.be/dynlib/) that allows searching known and unknown CID spectra of the profiled maize compounds in the DynLib database is available (Fig. 4).

**Table 2:** Number of characterized compounds per compound class.

Compound class	# characterized compounds	Organ distribution
(neo)lignan	8	S, L > T, E, C
amine	1	
amino acid	21	T > E, C, L, S
apocarotenoid	1	
benzenoid	22	T > S, L, E, C
benzofuran	1	
benzoxazinoid	15	E, L > C, T, S
C-glycosylated flavonoid	36	L, T > S, E, C
coumarin	1	
dioic acid	3	
flavonoid	5	
flavonolignan	28	L, S, T > E, C
gibberellin	1	
glutathione	6	
indole	22	T > C > S, L, E
mixed glycosides	77	T > L, S, E, C
monolignol	2	
monoterpenoid	1	
nucleoside	5	
O-glycosylated flavonoid	40	L, S, T > E, C
oligolignol	14	S > E, T > L, C
organic acid	9	E, C > L, S, T
oxylipin	9	S, E, T, C > L
phenethylamine	1	
phenol	3	
phenylethanoid	4	
phenylpropanoid	82	All organs
phosphate	3	
quinoline	1	
sugar	4	
vitamin	1	
Total	427	

Organ distributions are shown for compound classes that have at least eight members. C, late cob; E, ear; L, leaf; S, stem; T, tassel.

### 3.4 Mass differences of feature pairs

CSPPs are pairs of features of which the mass difference and elution order correspond to those expected for known organic reactions [35]. In order to obtain insight into the extent in which such organic reactions are enriched in specific organs, we searched for mass differences between pairs of LC-MS features that frequently occur in a given organ. To this end, all mass differences between 0 and 250 Da in intervals of 0.001 Da were computed in the FTneg and Ftpos raw data sets of the five different maize organs from the four genotypes (Fig. 2C, Supplemental Fig. 1C and 12). Following an organ- and genotype-wide PCA performed on the number of feature pairs for each of these 250,000 mass differences, the two highest-order PCs explained 56% (Fig. 2B) and 35% (Supplemental Fig. 1B) of the variation within the FTneg and FTpos raw data sets, respectively. These data indicate that specific mass differences occur more frequently in a given organ as compared to the other organs.

Most of the 250,000 mass differences do not reflect any combination between the chemical elements that occur most commonly in living organisms, i.e., C, H, O, N, P, and S elements, indicating that these mass differences do not reflect true biochemical conversions. Furthermore, in some cases for which a chemical formula can be computed from a mass difference, a chemically valid structure cannot be drawn (for example, the mass difference of 17.039 Da corresponds to CH_5_). To ease the interpretation of the data, we enriched for true biotransformations based on the assumption that their associated mass differences should occur more frequently than mass differences that do not reflect biotransformations. Therefore, a Manhattan plot was constructed that reflects the frequency of each of the 250,000 mass differences (Fig. 2C and Supplemental Fig. 1C). From such a Manhattan plot, prevailing mass differences, hereafter called ‘candidate biotransformations’, were selected [35]. Manhattan plots were constructed for each organ and for the FTneg and FTpos raw data sets (Supplemental Fig. 12). From the raw data sets of the FTneg, 107 candidate biotransformations were selected (Table 3) and for the FTpos, 167 candidate biotransformations (Supplemental Data Set 2). Upon PCA, 45% (PC1 and PC2; Fig. 2D) and 38% (PC1 and PC2; Supplemental Fig. 1D) of the variation within the selected candidate biotransformation sets of the FTneg and FTpos raw data sets were explained, respectively. Regarding the FTneg raw data set, the tassel, leaf, and stem profiles were present in three distinct clusters, whereas the ear and late cob profiles clustered together (Fig. 2D). For the FTpos raw data set, the ear and stem samples, the leaf and late cob samples, and the tassel samples formed three distinct clusters (Supplemental Fig. 1D). Similar to the PCA results obtained on feature abundances, most of the variation in biotransformation frequencies was among organs rather than among genotypes.

**Table 3:** Candidate biotransformations derived from the FTneg raw data set.

m/z	Chemical Formula	Putative Conversion	Short	Organ [OOI(avOOI)]					Class
				EAR	LATE COB	LEAF	STEM	TASSEL	
0.015	–	Unknown	UNK1	716 (911)	1180 (1339)	1249 (1468)	293 (453)	**17 (499)**	–
0.021	–	Unknown	UNK2	603 (838)	994 (1213)	1094 (1209)	508 (661)	**14 (460)**	–
0.036	–	MET + RED - OXY	DIFF1	84 (137)	112 (229)	170 (198)	48 (113)	**3 (115)**	1
0.037	–	MET + RED - OXY	DIFF1	387 (553)	522 (711)	504 (582)	236 (334)	**18 (740)**	1
1.003	–	^13^C - ^12^C	13C	**2 (11)**	**37 (49)**	91 (111)	**5 (7)**	**1 (2)**	–
1.004	–	^13^C - ^12^C	13C	121 (166)	325 (336)	438 (537)	64 (114)	**5 (26)**	–
1.979	–	OXY - MET	DIFF2	78 (125)	84 (194)	139 (173)	64 (93)	**6 (91)**	1
1.980	–	OXY - MET	DIFF2	247 (347)	369 (532)	352 (392)	294 (418)	**8 (87)**	1
2.015	H_2_	Reduction	RED	**7 (11)**	**14 (32)**	**24 (33)**	**10 (18)**	**4 (45)**	1
2.016	H_2_	Reduction	RED	**1 (1)**	**1 (1)**	**1 (2)**	**1 (1)**	**1 (7)**	1
3.995	-	MO	MO1	65 (93)	48 (111)	55 (79)	45 (73)	**7 (15)**	4
4.031	H_4_	MO	MO2	44 (91)	**26 (50)**	70 (86)	66 (97)	**15 (263)**	4
12.000	C	^12^C addition	12C	**26 (43)**	**30 (61)**	**23 (36)**	**18 (24)**	**25 (27)**	1
15.995	O	Oxygenation	OXY	**1 (3)**	**3 (3)**	**1 (1)**	**4 (5)**	**1 (5)**	1
18.011	H_2_O	Hydration	HYD	**14 (35)**	**25 (47)**	77 (95)	**30 (44)**	**37 (57)**	1
27.995	CO	MO	MO3	65 (101)	64 (81)	66 (95)	93 (102)	**36 (41)**	4
28.031	C_2_H_4_	Ethylene addition	ETH	126 (145)	57 (81)	**32 (55)**	152 (221)	166 (186)	2
30.010	CH_2_O	OXY + MET	MOX	**3 (5)**	**4 (7)**	**4 (5)**	**1 (3)**	**3 (7)**	1
30.011	CH_2_O	OXY + MET	MOX	**12 (16)**	**29 (58)**	44 (56)	**6 (11)**	**6 (10)**	1
32.026	CH_4_O	MOX + RED	MXRD	**25 (29)**	**16 (29)**	**25 (41)**	**26 (44)**	50 (74)	1
39.995	C_2_O	MO	MO4	**35 (72)**	**34 (84)**	49 (74)	56 (100)	144 (181)	4
42.010	C_2_H_2_O	Acetylation	ACE	47 (67)	**20 (42)**	**26 (37)**	**40 (59)**	53 (74)	1
44.026	C_2_H_4_O	MET + MOX	MTMX	**27 (46)**	**13 (34)**	**19 (24)**	**35 (51)**	**30 (37)**	1
46.005	CH_2_O_2_	Formic acid addition	FORM	**3 (4)**	**4 (5)**	**4 (6)**	**3 (4)**	**3 (6)**	-
56.026	C_3_H_4_O	MOX + ETY	MXETY	**7 (12)**	**8 (9)**	**6 (6)**	**10 (22)**	**17 (20)**	1
58.005	C_2_H_2_O_2_	OXY + ACE	OXYACE	42 (82)	84 (102)	67 (95)	**27 (60)**	62 (81)	1
60.021	C_2_H_4_O_2_	Cross ring cleavage	CRC60	**20 (28)**	**18 (19)**	**17 (18)**	**8 (13)**	**7 (13)**	–
70.005	C_3_H_2_O_2_	CAR + ETY	CRETY	**30 (54)**	**28 (41)**	**20 (29)**	**24 (41)**	71 (104)	1
72.021	C_3_H_4_O_2_	MOX + ACE	MXACE	**10 (11)**	**7 (8)**	**8 (10)**	**9 (12)**	**6 (11)**	1
74.036	C_3_H_6_O_2_	Glycerol coupling	GLY	82 (104)	55 (74)	54 (83)	**33 (95)**	122 (194)	1
76.016	C_2_H_4_O_3_	PEN - MOX - ETY	DIFF3	56 (92)	119 (149)	104 (125)	**38 (61)**	**30 (45)**	1
80.026	C_5_H_4_O	HQL - MO3	DIFF4	**22 (34)**	**28 (49)**	**24 (43)**	47 (72)	48 (84)	2
82.041	C_5_H_6_O	MO	MO5	**24 (65)**	92 (109)	67 (94)	89 (193)	233 (240)	4
84.021	C_4_H_4_O_2_	Unknown	UNK3	42 (90)	48 (99)	63 (118)	48 (101)	**23 (35)**	-
84.057	C_5_H_8_O	MO	MO6	62 (146)	107 (118)	**29 (39)**	255 (367)	248 (274)	4
86.000	C_3_H_2_O_3_	Unknown	UNK4	**20 (55)**	**10 (21)**	40 (45)	**20 (28)**	98 (130)	–
88.016	C_3_H_4_O_3_	Glyceric acid coupling	GLC	75 (88)	108 (132)	80 (92)	36 (64)	**38 (67)**	1
90.031	C_3_H_6_O_3_	Cross ring cleavage	CRC90	**23 (33)**	**22 (36)**	**12 (20)**	**21 (26)**	**29 (42)**	–
92.026	C_6_H_4_O	Phenol coupling	QUL	58 (94)	89 (136)	90 (103)	**24 (38)**	**21 (39)**	3
96.021	C_5_H_4_O_2_	DHBA - ETY	DIFF5	**23 (30)**	**19 (41)**	36 (48)	49 (57)	**32 (51)**	3
98.036	C_5_H_6_O_2_	Unknown	UNK5	**16 (22)**	**8 (17)**	**11 (19)**	53 (84)	44 (57)	–
100.052	C_5_H_8_O_2_	MO	MO7	49 (98)	**24 (63)**	**14 (39)**	75 (137)	125 (156)	4
102.031	C_4_H_6_O_3_	PEN - MOX	DIFF6	**32 (45)**	**38 (59)**	**31 (48)**	**24 (30)**	**25 (29)**	1
104.026	C_7_H_4_O	Benzoylation	BEN	146 (168)	103 (148)	177 (281)	**23 (26)**	**32 (48)**	3
104.047	C_4_H_8_O_3_	Unknown	UNK6	45 (85)	**29 (57)**	46 (53)	**30 (44)**	43 (78)	–
106.026	C_3_H_6_O_4_	PEN - ETY	DIFF7	46 (84)	**27 (135)**	138 (166)	51 (62)	92 (120)	1
108.021	C_6_H_4_O_2_	Quinone coupling	HQL	84 (125)	56 (84)	87 (100)	67 (80)	**43 (52)**	2
110.036	C_6_H_6_O_2_	Unknown	UNK7	**34 (79)**	45 (90)	61 (90)	102 (126)	89 (113)	-
112.052	C_6_H_8_O_2_	Mevalonolactone coupling	MEV	**22 (54)**	**27 (59)**	**27 (34)**	**34 (70)**	51 (85)	1
114.031	C_5_H_6_O_3_	GLC + ETY	GLCETY	**17 (21)**	**17 (27)**	**19 (29)**	**17 (29)**	**27 (31)**	1
116.010	C_4_H_4_O_4_	Unknown	UNK8	162 (196)	139 (184)	191 (219)	**34 (64)**	121 (205)	–
116.047	C_5_H_8_O_3_	PEN - OXY	DIFF8	**23 (44)**	**17 (22)**	**12 (14)**	**31 (39)**	**34 (37)**	1
118.041	C_8_H_6_O	Phenylacetyl coupling	PHA	316 (372)	242 (369)	329 (424)	263 (349)	**44 (121)**	3
120.021	C_7_H_4_O_2_	Hydroxybenzoylation	HBEN	119 (170)	72 (149)	133 (168)	86 (99)	**20 (29)**	3
120.042	C_4_H_8_O_4_	Cross ring cleavage	CRC120	91 (134)	75 (84)	77 (107)	**35 (54)**	68 (91)	–
122.036	C_7_H_6_O_2_	Dihydroxybenzylalcohol coupling	DHBA	**21 (73)**	46 (73)	35 (61)	**29 (48)**	63 (83)	3
124.052	C_7_H_8_O_2_	Unknown	UNK9	52 (67)	**38 (65)**	**30 (41)**	51 (80)	86 (144)	–
126.031	C_6_H_6_O_3_	DHBA - ETY + MOX	DIFF9	**23 (45)**	**32 (59)**	**23 (41)**	42 (80)	63 (83)	3
128.047	C_6_H_8_O_3_	Unknown	UNK10	**36 (92)**	75 (93)	51 (66)	82 (120)	72 (79)	–
130.026	C_5_H_6_O_4_	MO	MO8	**9 (14)**	**5 (25)**	**13 (16)**	**21 (30)**	**14 (25)**	4
130.062	C_6_H_10_O_3_	HEX - OXY - OXY	DIFF10	67 (96)	64 (69)	**21 (36)**	98 (168)	97 (128)	1
132.041	C_5_H_8_O_4_	Pentose coupling	PEN	53 (99)	106 (131)	104 (141)	92 (115)	**42 (103)**	1
132.042	C_5_H_8_O_4_	Pentose coupling	PEN	60 (90)	49 (102)	52 (84)	**26 (57)**	**26 (67)**	1
134.036	C_8_H_6_O_2_	PHA + OXY	HPHA	43 (80)	**15 (29)**	32 (59)	**22 (36)**	**15 (23)**	3
136.052	C_8_H_8_O_2_	Unknown	UNK11	46 (97)	98 (155)	109 (123)	**31 (38)**	61 (75)	–
142.026	C_6_H_6_O_4_	Unknown	UNK12	**20 (34)**	**21 (52)**	43 (52)	61 (76)	**40 (65)**	–
144.041	C_6_H_8_O_4_	Hydroxyadipic acid coupling	HADI	51 (78)	43 (95)	76 (83)	87 (91)	**12 (38)**	1
144.042	C_6_H_8_O_4_	Hydroxyadipic acid coupling	HADI	**32 (89)**	62 (97)	57 (91)	63 (88)	60 (129)	1
146.057	C_6_H_10_O_4_	Deoxyhexosylation	RHA	**8 (14)**	**6 (7)**	**5 (6)**	**11 (16)**	**8 (25)**	1
150.031	C_8_H_6_O_3_	Vanillic acid coupling	VAN	**37 (90)**	62 (99)	107 (116)	**35 (42)**	**40 (54)**	3
150.052	C_5_H_10_O_5_	MO	MO9	120 (164)	120 (181)	98 (123)	82 (140)	**28 (60)**	4
154.062	C_8_H_10_O_3_	Unknown	UNK13	78 (103)	71 (137)	39 (40)	**34 (64)**	59 (119)	–
162.052	C_6_H_10_O_5_	Hexosylation	HEX	**2 (4)**	**1 (2)**	**2 (3)**	**2 (3)**	**2 (5)**	1
174.052	C_7_H_10_O_5_	Quinic acid coupling	QUI	**19 (50)**	**27 (51)**	**15 (17)**	**42 (52)**	**31 (50)**	1
178.047	C_6_H_10_O_6_	HEX + OXY	HXOXY	**10 (23)**	**12 (36)**	38 (51)	**28 (38)**	**10 (17)**	1
178.062	C_10_H_10_O_3_	Condensed guaiacyl coupling	GUN8	**32 (49)**	41 (82)	94 (116)	**17 (35)**	70 (121)	3
180.041	C_9_H_8_O_4_	Syringic acid coupling	SYR	70 (162)	**35 (102)**	35 (47)	**19 (90)**	86 (125)	3
180.042	C_9_H_8_O_4_	Syringic acid coupling	SYR	104 (167)	91 (126)	121 (140)	**41 (72)**	155 (188)	3
180.078	C_10_H_12_O_3_	GUN8 + RED	GN8RD	**12 (36)**	**31 (52)**	**33 (68)**	**13 (16)**	56 (220)	3
182.057	C_9_H_10_O_4_	Unknown	UNK14	39 (73)	**29 (55)**	39 (53)	**27 (45)**	**43 (53)**	–
186.052	C_8_H_10_O_5_	MO	MO10	**25 (49)**	**15 (25)**	**13 (22)**	47 (88)	**36 (46)**	4
192.062	C_7_H_12_O_6_	HEX + MOX	HXMX	70 (234)	160 (212)	**33 (70)**	77 (122)	**11 (36)**	1
194.057	C_10_H_10_O_4_	FA + HYD	FAHYD	**6 (11)**	**6 (14)**	**10 (19)**	**7 (10)**	**24 (43)**	3
196.036	C_9_H_8_O_5_	Unknown	UNK15	40 (81)	**33 (51)**	**18 (21)**	79 (98)	**14 (43)**	–
196.073	C_10_H_12_O_4_	Non-condensed guaiacyl coupling	GUN4	**17 (29)**	**14 (35)**	**18 (29)**	**15 (23)**	**22 (113)**	3
198.052	C_9_H_10_O_5_	Unknown	UNK16	84 (137)	**22 (54)**	48 (75)	60 (101)	**13 (37)**	–
204.062	C_8_H_12_O_6_	HEX + ACE	HXACE	63 (119)	83 (146)	37 (56)	90 (170)	**27 (76)**	1
206.057	C_11_H_10_O_4_	Sinapic acid coupling	SIN	**15 (23)**	**8 (32)**	**26 (32)**	**14 (20)**	**26 (47)**	3
208.073	C_11_H_12_O_4_	Condensed syringyl coupling	SUN8	**15 (23)**	**12 (23)**	**27 (59)**	**7 (12)**	**11 (18)**	3
210.052	C_10_H_10_O_5_	SYR + MOX	SRMX	**14 (29)**	**16 (23)**	**8 (10)**	**26 (33)**	**16 (33)**	3
210.088	C_11_H_14_O_4_	MO	MO11	34 (125)	72 (147)	75 (129)	**27 (60)**	250 (365)	4
212.067	C_10_H_12_O_5_	MO	MO12	**37 (51)**	42 (107)	70 (92)	71 (85)	97 (115)	4
214.047	C_9_H_10_O_6_	Unknown	UNK17	105 (130)	**29 (63)**	74 (98)	91 (153)	42 (78)	–
222.052	C_11_H_10_O_5_	HQL + RHA - MOX - RED	DIFF11	29 (37)	**12 (19)**	39 (63)	**30 (59)**	**39 (53)**	2
224.067	C_11_H_12_O_5_	HQL + RHA - MOX	DIFF12	**8 (24)**	**11 (21)**	**15 (29)**	**11 (27)**	**23 (47)**	2
224.068	C_11_H_12_O_5_	HQL + RHA - MOX	DIFF12	54 (118)	**23 (90)**	74 (109)	57 (84)	**18 (45)**	2
226.047	C_10_H_10_O_6_	Unknown	UNK18	59 (95)	**33 (56)**	38 (68)	60 (163)	**11 (30)**	-
226.083	C_11_H_14_O_5_	Non-condensed syringyl coupling	SUN4	**9 (21)**	**11 (21)**	**8 (9)**	**6 (9)**	**19 (43)**	3
236.067	C_12_H_12_O_5_	SIN + MOX	SNMX	**9 (40)**	**10 (25)**	**10 (14)**	36 (52)	**10 (14)**	3
238.047	C_11_H_10_O_6_	HQL + RHA - MET - RED	DIFF13	41 (62)	42 (62)	**30 (65)**	120 (140)	78 (103)	2
238.083	C_12_H_14_O_5_	GUN4 + ACE	GN4ACE	**9 (22)**	**17 (40)**	**16 (25)**	**10 (15)**	**25 (59)**	3
240.062	C_11_H_12_O_6_	HQL + HEX - MOX	DIFF14	44 (71)	**31 (43)**	56 (79)	61 (108)	**33 (47)**	2
242.078	C_11_H_14_O_6_	HQL + HEX - MOX + RED	DIFF15	**6 (21)**	**4 (9)**	**9 (15)**	**8 (10)**	**19 (36)**	2
246.073	C_10_H_14_O_7_	Unknown	UNK19	**33 (94)**	102 (145)	41 (54)	158 (216)	96 (180)	–
246.109	C_11_H_18_O_6_	Unknown	UNK20	56 (141)	133 (157)	**33 (126)**	180 (268)	513 (704)	–
248.067	C_13_H_12_O_5_	Unknown	UNK21	**8 (79)**	**13 (100)**	**29 (66)**	**25 (50)**	**7 (37)**	–
248.068	C_13_H_12_O_5_	Unknown	UNK21	93 (230)	99 (194)	271 (402)	125 (157)	**33 (74)**	–

Mass difference frequencies, displayed in Manhattan plots, were computed for all genotypes and all organs. Mass differences are given whenever their frequencies surpassed the local frequency threshold (the frequency threshold varied dependent on the considered mass difference; see Morreel *et al*. (2014) [35]) in an organ of at least one of the four genotypes; in these organs, the mass difference frequency is shown in bold and underlined. For each organ and each genotype, the frequencies of all mass differences were normalized and ranked. Normalization was based on the division of the frequency of the mass difference by its local frequency threshold. These normalized mass difference frequencies were then ranked in decreasing order and an order-of-importance (OOI) number was assigned in increasing order (mass differences that show a high frequency obtained a low OOI number). The OOI number given for the considered mass difference in the table, corresponds with that of the genotype having the lowest OOI number among all genotypes for the particular organ. To account for the variation in OOI number for a particular mass difference among the genotypes, the average OOI (avOOI) value across the genotypes was computed and is given between parentheses. Abbreviations used when naming the ‘Putative Conversion’ can be traced in the ‘Short’ column; the full name is then mentioned in the ‘Putative Conversion’ column. Five abbreviations that cannot be found in the ‘Short’ column are CAR, carboxylation (CO_2_, 43.990 Da); ETY, ethyne addition (C_2_H_2_, 26.016 Da); FA, feruloylation (C_10_H_8_O_3_, 176.047 Da); MET, methylation (CH_2_, 14.016 Da); and MOX, oxygenation (OXY) + methylation (MET) (CH_2_O, 30.011 Da). A + and – sign indicate that the second putative conversion represents an addition and elimination, respectively. Class 1, 2, 3 and 4 represent ‘decoration’-type, ‘structural’-type, ‘core transfer’-type and ‘multiple options’ (MO)-type biotransformations, respectively.

### 3.5 Obtaining insight into organ-preferential biotransformations via CSPP networks

**Fig. 5:**
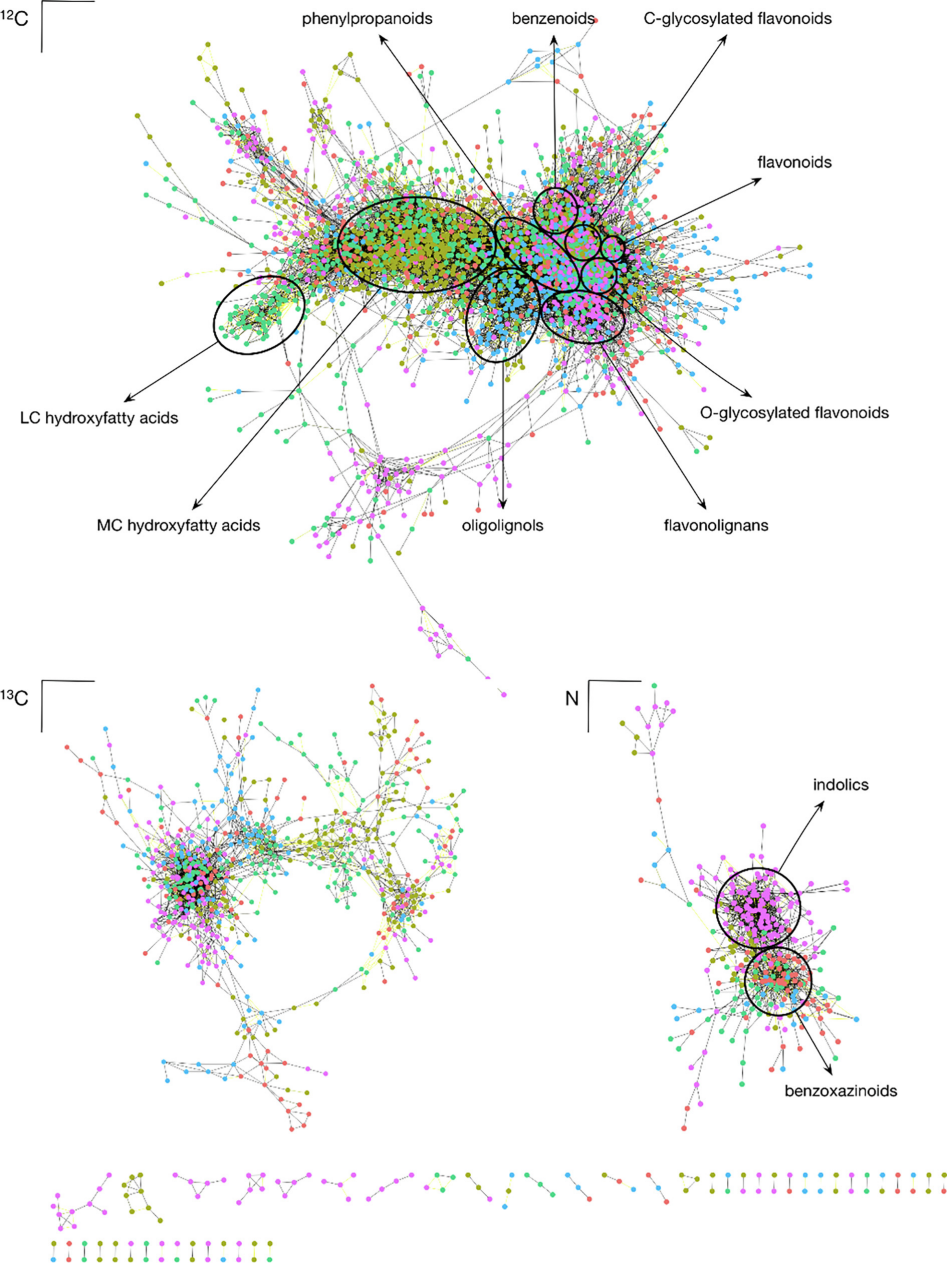
CSPP Network Based on the FT Chromatograms Using Negative Ionization Mode. The node color (red, greenish brown, green, blue and purple) represents the organ in which the feature was the most abundant (the ear, late cob, leaf, stem or tassel, respectively). The color of the network edges shows the MS^2^ similarity of the substrate and the product. LC, long chain; MC, medium chain. (For interpretation of the references to color in this figure legend, the reader is referred to the web version of this article.)

In an attempt to gain insight into the organ specificity or organ enrichment of the candidate biotransformations, CSPP networks were constructed based on the selected biotransformations (Table 3 and Supplemental Data Set 2) for the FTneg (Fig. 5) and FTpos (Supplemental Fig. 13) raw data sets. In both CSPP networks, nodes represent features for which CID spectra were recorded, i.e., features that were present in the DynLib database. Based on the annotated features in the DynLib database, which were characterized via RDynLib, compound information was added to the network nodes.

The CSPP networks constructed from the features present in the FTMS_neg and FTMS_pos subDBs contained 3,505 and 652 nodes, and 32,252 and 2,552 edges, respectively. Besides the main sub-network, the FTMS_neg-based CSPP network (Fig. 5) contained two medium-sized sub-networks, which predominantly represented N-containing compounds and the natural ^13^C isotope distribution of the profiled compounds. In the network, different clusters were associated with different compound classes. When considering the node color, which represents the organ in which the feature was the most abundant, different regions in the network became apparent. For example, nodes representing oligolignols appeared as a cluster and were mainly present in the LC-MS profiles of stem extracts (Fig. 5, blue), whereas a cluster of nodes associated with medium-chain-length hydroxyfatty acids was predominantly found in late cob and leaf extracts (Fig. 5, greenish brown and green). Similarly, a distinction between benzoxazinoids and dihydroxyindole-3-acetic acid derivatives was observed in the sub-network of the N-containing compounds, with the latter compound class being almost solely present in the tassel (Fig. 5, purple). The FTMS_pos-based CSPP network consisted of only one large sub-network, in which clusters of features that were more abundant in particular organs could be distinguished (Supplemental Fig. 13).

The CSPP networks were subsequently consulted to characterize the included candidate biotransformations derived from the FTneg (Table 3) and FTpos raw data sets (Supplemental Data Set 2). In addition to mass differences that were associated with a single conversion, some reflected a unique combination of conversions [e.g., oxygenation + methylation + reduction (32.026 Da)], whereas others remained unassigned because they corresponded to multiple possible combinations of conversions (multiple options or MO).

The ten candidate biotransformations from which the frequencies contributed the most to the PCA-based discrimination between the different organs, based on the FTneg raw data set, are visualized in Fig. 2D. These biotransformations were associated with mass differences of 28.031 Da, 84.057 Da and 130.062 Da (enriched in leaf), 118.041 Da, 144.041 Da and 150.052 Da (enriched in tassel), 30.011 Da (enriched in ear, late cob, stem, and tassel), 104.026 Da (enriched in stem and tassel), 180.078 Da (enriched in ear, late cob, leaf, and stem) and 39.995 Da (enriched in ear and late cob). Based on the annotated features in the DynLib database, six of these biotransformations could be annotated as the addition of phenylacetic acid (118.041 Da), benzoic acid (104.026 Da), ‘condensed guaiacyl’ + reduction [180.078 Da; ‘condensed guaiacyl’ is the result of a radical–radical coupling of coniferyl alcohol into oligolignols/(neo)lignans via a condensed bond (i.e., characterized by an 8–5 or 8–8 interunit bond)], ethylene (28.031 Da; generally observed as the mass difference between a benzoic acid and a dehydrated phenylpropanoid), hydroxyadipic acid (144.041 Da), and a methoxy group (30.011 Da).

A PCA-based selection of the ten most important organ-discriminating candidate biotransformations (Supplemental Fig. 1D) derived from the FTpos raw data set included mass differences of 134.037 Da, 189.043 Da, and 207.054 Da (enriched in tassel), 42.011 Da (enriched in ear, late cob, leaf and tassel), 130.063 Da (enriched in leaf), 2.016 Da and 44.026 Da (enriched in all organs), 58.042 Da (enriched in late cob and leaf), 21.982 Da (enriched in ear, late cob and stem) and 88.053 Da (enriched in ear, stem and tassel). Of these biotransformations, the conversion could be tentatively assigned for 2.016 Da (reduction), 42.011 Da (acetylation), 44.026 Da (methylation + oxygenation + methylation), 134.037 Da (addition of phenylacetic acid + oxygenation), and 189.043 Da (addition of dihydroxyindole-3-acetic acid).

In a search for the organ distribution of the candidate biotransformations, we visualized all prevalent FTneg biotransformations (see Table 3) in a Venn diagram (Fig. 2E). Twenty-eight candidate biotransformations occurred frequently in all organs. These mass differences were general biotransformations, such as reduction, oxygenation, hexosylation, sinapic acid coupling or syringyl coupling. The Venn diagram also shows biotransformations that were prevailing in only one or a few organs. Thirty-nine candidate biotransformations were enriched in only one particular organ (ranging from two in the late cob to 19 in the tassel; Fig. 2E). Among them, four were assigned to a single conversion (Table 3): coupling/insertion of ethylene (28.031 Da, leaf), glycerol (74.036 Da, stem), glyceric acid (88.016 Da, tassel), and hydroxybenzoic acid (120.021 Da, tassel).

For the FTpos raw data set, 24 candidate biotransformations (Supplemental Data Set 2) occurred frequently in all organs (Supplemental Fig. 1E). Seventy-nine candidate biotransformations (Supplemental Fig. 1E) were solely enriched in one particular organ, of which nine were tentatively assigned as a single conversion (Supplemental Data Set 2): addition of carboxylic acid (43.990 Da, leaf), phenol (92.026 Da, ear), hydroxybenzyl alcohol (106.042 Da, stem), phenylacetic acid (118.042 Da, tassel), dihydroxybenzyl alcohol (122.037 Da, stem), vanillic acid (150.032 Da, stem), ferulic acid (176.048 Da, ear), ‘condensed guaiacyl’ (178.063 Da, stem), and syringic acid (180.043 Da, stem). Compared to the FTneg raw data set, the FTpos raw data set revealed more biotransformations that were prevailing in one particular organ. To conclude, the combination of different reversed-phase LC-MS methods with CSPP-networks, allowed the structural characterization of a collection of specialized metabolites and biotransformations, which is a critical necessity in the understanding of the maize phenolic metabolism.

## 4 Discussion

### 4.1 Structural characterization is enhanced using spectral metadata

The structural annotation of the many unknown compounds in metabolome data has been limited by the low coverage and slow growth of CID spectral libraries [20]. Consequently, only a minority of the profiled specialized metabolites can be annotated through matching their CID spectra with CID spectral libraries. This limitation was confirmed by our observation that only 0.93% up to 2.46% (dependent on the DynLib subDB) of the unique CID spectra of the maize specialized metabolome could be annotated through matching with publicly available spectral databases. The inclusion of all DynLib subDBs, and thus different types of CID spectra, for spectral matching led to more annotations than could be obtained for any of the individual subDBs. For the “library-matched and MS/MS-MS^n^-aligned” CID spectra in the FTMS_neg, QTOF_neg, FTMS_pos and QTOF_pos DynLib subDBs, 20% (3/15), 19% (3/16), 25% (3/12), and 43% (3/7), respectively, were annotated via both MS/MS and MS^n^ spectral matching. Thus, the majority of these CID spectra were annotated via either its MS/MS or its MS^n^ spectrum. This positive increase in spectral matches by considering different types of CID spectra resulted in part from the low compound overlap between the different DynLib subDBs, which is acknowledged by the rather low number of features for which both MS/MS and MS^n^ spectra were recorded (Table 1). Furthermore, the added value of including all DynLib subDBs for spectral matching is the consequence of two limitations of currently existing spectral databases. Firstly, spectral databases do not fully overlap; part of each spectral database represents spectral data from compounds for which CID spectra are unavailable in other spectral databases. Secondly, these spectral databases are often focused on a particular type of CID spectrum, i.e., MS/MS or MS^n^ spectra. Whereas the former limitation implies an augmented annotation rate when matching with several spectral databases instead of just one, the latter limitation guarantees more success in structural annotation when different types of CID spectra for a given compound are available for spectral matching. With the RDynLib package, we enabled linking of the MS/MS and MS^n^ spectral data belonging to a given feature, thereby increasing the chance of finding a spectral match for this feature with publicly available spectral databases, now also including the DynLib database.

Nonetheless, for the majority of the profiled features there is no CID spectrum available in the currently existing spectral databases, which prevents the annotation of the unknown compound by spectral matching. However, the structure of the corresponding compounds might be present in compound databases such as the PubChem database [46]. Upon computation of the chemical formula of the unknown feature, candidate structures can be retrieved from these compound databases. Subsequently, the CID spectra of these candidate structures can be predicted *in silico* and matched against the unknown CID spectrum. Consequently, *de novo* CID spectral elucidation software, such as MetFrag [28] and CSI:FingerID [22], have been developed. *De novo* CID spectral elucidation software takes full advantage of the large size of these compound databases, sometimes comprising tens of millions of compounds. In this study, 168 of the 427 characterized compounds (i.e., 39.3%; Supplemental Data Set 1) were present in the PubChem database. Installing a connection between the DynLib database and *de novo* CID spectral elucidation software would therefore further enhance the efficiency of compound characterization.

Structural characterization of metabolites by CSPP network propagation also benefits from including as much MS data as possible. For example, the complementary information of negative and positive CID spectra allows a distinction between charge-driven and charge-remote fragmentations [47]. In addition, the spectral information gained from QTOF-MS and IT-MS instruments is complementary. Whereas QTOF-based MS/MS spectra offer a higher mass accuracy of the product ions and also display the low-mass product ions, IT-based MS^n^ spectra allow the relationships between the product ions to be delineated. Furthermore, CID in IT-MS is less energetic than in QTOF-MS, which partially prevents further fragmentation of the initially formed first-order product ions. The longer timescale of CID reactions in IT-MS as compared to those in QTOF-MS coincides with the higher importance of thermodynamic control during CID in IT-MS as compared to kinetic control in QTOF-MS. As an example, Supplemental Fig. 15 displays the complementarity between MS/MS and MS^2^ upon CID of the diferuloyl glycerol anion. To date, the RDynLib package is equipped with a battery of tools to aid in structural elucidation (see Supplemental Text, *Structural characterization tools in RDynLib*; Supplemental Figs. 5-11). The combination of complementary CID spectra and their simultaneous analysis using the RDynLib tools, and the interplay with CSPP network propagation, played an important role in the elucidation of the 427 characterized compounds, of which the majority had not been described in maize before.

### 4.2 Compound abundances as well as biotransformation frequencies reflect organ-preferential metabolism

The annotated biotransformations displayed in Table 3 can be grouped into four classes. The first class represents mass differences corresponding to true (bio)chemical conversions, such as hexosylation (HEX), methylation (MET), and reduction (RED). Sometimes they reflect the subsequent action of two or more enzymes such as an oxygenation followed by a methylation (MOX). The CID spectra of the CSPP substrates and products of these biotransformations often show a high spectral similarity. Some of these biotransformations or combinations of biotransformations seem to be rather specific to particular taxa. For example, the combination of carboxylation and ethylation (CAR + ETH) was not enriched in the CSPP networks derived from leaf extracts of Arabidopsis [35], nor in those derived from the extracts of different poplar organs (data not shown). The biotransformations of this class seem to be involved in decorating core structures of specialized metabolism, e.g., a flavonoid or phenylpropanoid, and were suggested by Wang et al. (2019) [48] to occur following the biosynthesis of the core structure of the specialized metabolic class. However, some of these biotransformations seem to occur more frequently for particular classes of specialized metabolites. For instance, methoxylation (MOX, 30.011 Da; see Fig. 2D) was most frequently associated with oligolignol biosynthesis and, consequently, discriminated stem organs – in which oligolignols are enriched – from the other organs. This first class of biotransformations could be referred to as ‘decoration’-type biotransformations. Morreel *et al*. (2014) [35] argued that these CSPP biotransformations were often observed between glycosylated derivatives, whereas the corresponding enzymatic reactions are often known to occur on the aglycone level. For example, the conversion between caffeoyl hexose and feruloyl hexose is annotated as a methylation, yet, biochemically, this methylation reaction is known to use caffeoyl CoA as substrate. Therefore, a ‘decoration’-type biotransformation often includes multiple enzymatic reactions besides the annotated reaction.

Frequently occurring mass differences from the second class do not represent one or more true enzymatic reactions but rather the structural difference between two core structures from specialized metabolism. For example, the mass difference of 108.021 Da reflects the structural difference between a phenylpropanoid and a flavonoid, e.g., between dihydroxyindole-3-acetic acid (caffeoyl) hexoside and dihydroxyindole-3-acetic acid (eriodictyol-*O*-) hexoside. These ‘structural’-type biotransformations can only occur between two metabolites belonging to different specialized metabolic classes if their core structures share the same type and number of chemical modifications/decorations. Therefore, their occurrence supports the hypothesis that largely the same ‘decoration’-type biotransformations occur in the biosynthesis pathways of different specialized metabolic classes [48]. The CID spectra of the candidate substrates and products corresponding to different metabolic classes are dissimilar. Consequently, a lower average CID spectral similarity are expected for CSPPs representing this class of conversions as compared to those belonging to the first class. Although not reflecting enzymatic reactions, these ‘structural’-type biotransformations might aid deriving the biochemical pathways from the CSPP network by revealing the absence of particular pathway intermediates (Supplemental Fig. 24).

A third class of biotransformations includes true (bio)chemical conversions that involve the coupling of the core structure of a specific metabolic class onto another molecule. Among these ‘core transfer’-type biotransformations are phenylacetyl coupling (PHA), syringic acid coupling (SYR), and oligolignol unit extensions such as the (non–)condensed guaiacyl (GUN58 and GUN4) and syringyl (SUN58 and SUN4) coupling reactions. The large number of glycosides and sugar esters encountered among the characterized compounds (Supplemental Data Set 1) suggests that these ‘core transfer’-type biotransformations mainly happen onto a sugar moiety of a glycosylated molecule.

The fourth class of biotransformations are those that can arise via multiple, putative biotransformation pathways, each pathway often including multiple reactions. For instance, a mass difference of 3.995 Da can be explained by the mass difference between a hydrated and a methylated molecule (HYD - MET) as well as resulting from a methoxylation followed by the loss of an ethyne group (MOX - ETY). Thus, for these ‘multiple options’ (MO)-type biotransformations, the reactions within a particular candidate substrate and candidate product pair differ from those of another feature pair with the same mass difference. From the variety of possible reaction sequences, it might be expected that these MO-type biotransformations frequently occur in all organs, yet most of them seem to be restricted to only one or a few organs (Table 3). This suggests that a particular biotransformation pathway is prevailing for at least some of these mass differences, implying that some of these mass differences would better fit in one of the former biotransformation classes. The selection of the relevant, i.e. frequently occurring, mass differences was based on all LC-MS features, whereas structural characterization of the candidate substrates and products of the biotransformations was restricted to the LC-MS features for which CID spectra were recorded. Therefore, the correct annotation of the MO-type biotransformations might have been prevented by the sometimes low number of characterized compounds on which the characterization of the biotransformations, and thus the biotransformation classification, was based. Vice versa, biotransformations that were classified as ‘decoration’-, ‘structural’- or ‘core transfer’-type biotransformations might include feature pairs of which the mass difference results from an alternative biotransformation pathway than the one proposed in Table 3.

PCA-based organ clustering of the maize metabolome data sets was similar when using either biotransformation frequencies or feature abundances. This indicates that the separation between the different organs on the PCA plot based on biotransformation frequencies reflects both the different metabolic classes and the differentially enriched biotransformations in these organs. Consequently, many of the biotransformations that explain most of the variation between the PCA clusters represent ‘core transfer’-type biotransformations (180.078 Da, GUN58 + RED; 104.026 Da, BEN; 118.041 Da, PHA; 144.041 Da, HADI; Fig. 2D; Table 3). For example, oligolignols were enriched in stems, and the clustering of this organ upon PCA could be related to the frequent occurrence of the ‘GUN58 + RED’ biotransformation (180.078 Da; Fig. 2D; Table 3), reflecting the oxidative coupling of a lignin monomer. ‘Structural’-type biotransformations may also contribute to the PCA-based organ clustering (28.031 Da, ETH; Fig. 2D; Table 3). For example, the earlier mentioned mass difference of 108.021 Da occurs more frequently in the tassel (Table 3) than in other organs, and is the consequence of the rich variety of *O*- and *C*-glycosylated flavonoids in the tassel (Table 2) and their structural difference with the phenylpropanoid glycosides that are present in all organs. These ‘structural’-type biotransformations were generally observed between glycosylated molecules from different specialized metabolic classes. Therefore, the effect of the ‘structural’-type biotransformations on the PCA clustering results in part from the large number of glycosides present in the maize metabolome data set (Supplemental Data Set 1). The high number of glycosylated molecules among the characterized compounds highlights the large number of glycosyl- and acyltransferases operating in the maize specialized metabolism and/or the broad substrate specificities of these enzymes, allowing the anchoring of specialized metabolites from different classes onto the same sugar moieties. This results in the observation of many glycosylation and acylation reactions, and the great variety of high-molecular-weight (mixed) glycosides in the CSPP network. Many of these high-molecular-weight mixed glycosides represented concatenation products between phenylpropanoid glycosides and benzoic acids, flavonoids, phenylethanoids and indolics (Supplemental Data Set 1). Acylation is established by either cytosolic CoA-dependent BAHD acyltransferases or vacuolar/apoplastic acylsugar-dependent serine carboxypeptidase-like (SCPL) acyltransferases [49]. Many of the SCPL enzymes have a broad substrate specificity and are implemented in transesterification reactions, e.g., the formation of disinapoyl glucose from two sinapoyl glucoses [50], leading to, among others, the high-molecular-weight (mixed) glycosides in the vacuole [51], [52]. In addition, the presence of vacuolar glycoside hydrolases (GHs) that hydrolyze or rearrange glycosidic bonds suggests that these (mixed) glycosides are actively metabolized [53] and that the released sugars and aglycones can be exported to the cytosol [54]. Glycosylation and sugar ester formation are generally accepted as a strategy of the plant to detoxify, to increase the solubility, and/or to alter the biological activity of specialized metabolites [53]. However, the abundant presence of high-molecular-weight (mixed) glycosides and the metabolic malleability of the mixed glycosides suggest the importance of mixed glycoside biosynthesis as a strategy of the plant to store metabolites from different classes of primary and specialized metabolism. In addition, the coupling of multiple core structures onto one sugar unit might be essential in controlling the osmotic pressure and/or in reducing the sugar amount that would otherwise be trapped when storing low-molecular-weight glucosides.

### 4.3 Phenylpropanoids are prominent in all five investigated maize organs

The PCA plot based on feature abundances indicated more differences in the maize metabolome among different organ types than among different genotypes (Fig. 2A, Table 2). The metabolic fingerprint of the organ types was also reflected in the CSPP network. In the CSPP networks, features that are candidate substrates and products are linked via the selected candidate biotransformations. Consequently, compounds that belong to the same metabolic class typically represent sub-networks within the CSPP network. Coloring the nodes in the CSPP network, according to the organ in which the abundance of the corresponding feature was the highest, revealed a pattern closely associated with the metabolite class-based sub-networks (Fig. 5). For instance, indolics were more abundant in the tassel as compared to other organs, and oligolignols were more abundant in stem internodes. Based on the constructed CSPP networks, most specialized compound classes prevailed in a restricted set of maize organs. A notable exception were phenylpropanoids that were common in all organs (Table 2).

Among the phenylpropanoids, many *p*-coumarate esters/amides, and to a lesser extent caffeate and ferulate esters/amides, were observed. These phenylpropanoids were bearing moieties derived from glycolaldehyde (in its hydrate form, i.e., ethanetriol), 2-hydroxyglutaric acid, 2-hydroxyadipic acid, isocitric acid, putrescine, hexaric acid, threonic/erythronic acid, shikimic acid, hexose, quinic acid, glycerol, tyramine, and hydroxycitric acid. Many of these acids and amines are chiral and are therefore of interest for the chemical and pharmaceutical industries. For example, hydroxycitric acid has received a lot of attention owing to its anti-obesity effect [55], [56] and as a promising agent for the treatment of kidney stones [57], [58]. High concentrations of hydroxycitric acid have been found in a few tropical plant species such as *Hibiscus sabdariffa* [59] and *Garcinia* species [60], and extracts from the latter are already used as food ingredients or as dietary supplements [61], [62].

### 4.4 A variety of auxin storage forms differentially accumulate in late cob and tassel

A remarkable CSPP sub-network represented a variety of auxin-related compounds that were almost solely present in the late cob and the tassel (Fig. 5). Most of these compounds were not only never described in maize, but were also not present in the Pubchem database (Supplemental Data Set 1). Because the presence of many of these auxin-related compounds was often associated with specific maize organs, we used this class to illustrate the potential of our findings in the construction of putative biosynthetic pathways.

**Fig. 6:**
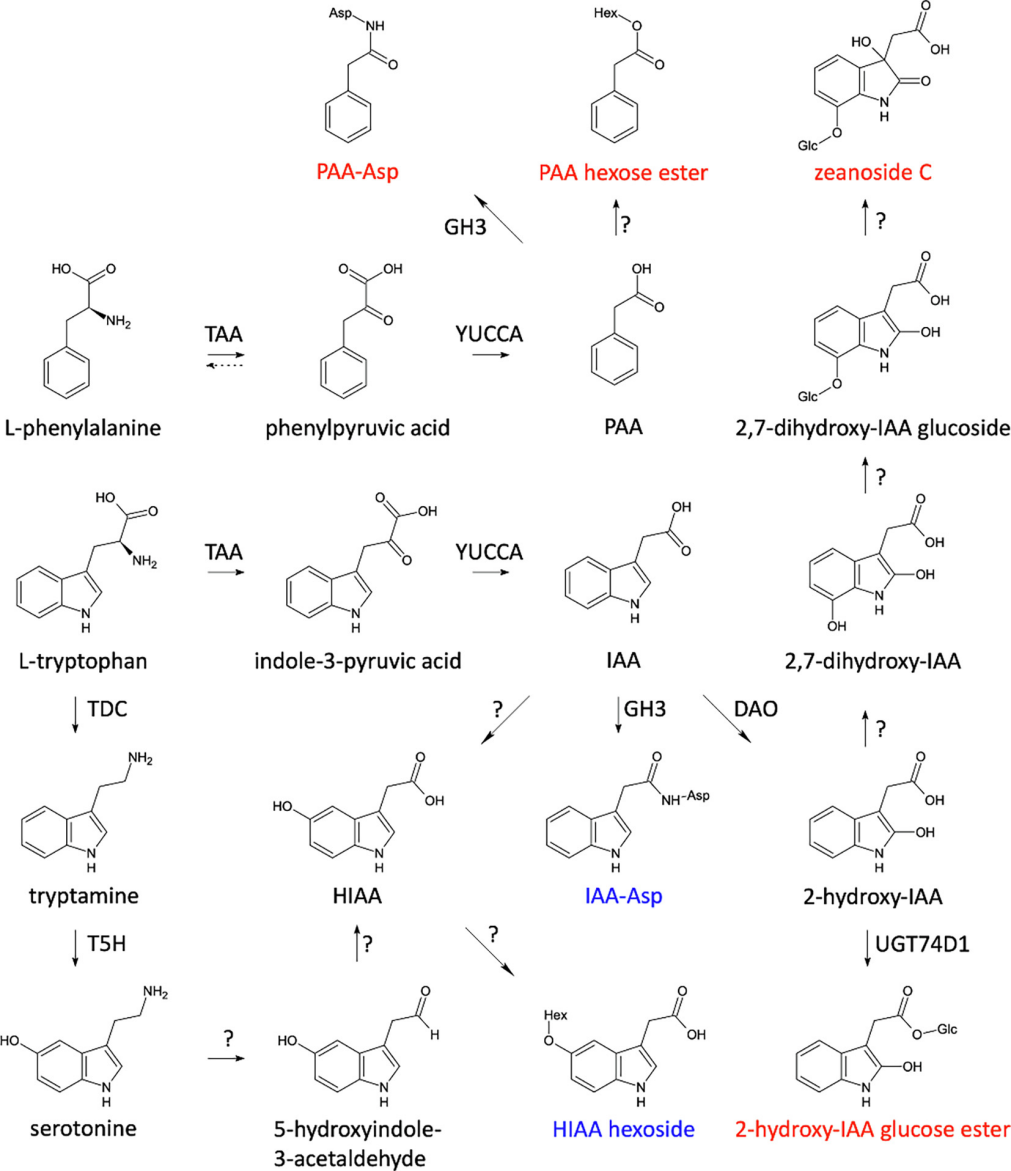
Auxin metabolism. Auxin derivates that were especially abundant in tassel and late cob are indicated in red and blue. A question mark above an arrow indicates a reaction that is not yet known to operate in plants. The dotted arrow indicates that phenylpyruvic acid is an intermediate in L-phenylalanine biosynthesis. DAO, DIOXYGENASE FOR AUXIN OXIDATION; GH3, GRETCHEN-HAGEN3; T5H, tryptamine-5-hydroxylase; TAA, TRP AMINOTRANSFERASE of ARABIDOPSIS; TDC, tryptophan decarboxylase; UGT, uridine diphosphate glycosyltransferase. (For interpretation of the references to color in this figure legend, the reader is referred to the web version of this article.)

The conjugation of aspartate or glutamate to indole-3-acetic acid (IAA), the core structure of the indolics, and to phenylacetic acid (PAA), the core structure of the phenylethanoids, is an important aspect of auxin homeostasis in plants [63], [64]. Of these four conjugates, only IAA aspartate and PAA aspartate were found in this study. IAA aspartate was detected at higher levels in the late cob than in the tassel, whereas the aspartate amide of PAA was more abundant in the tassel. IAA and PAA are both auxins and have been proposed to be synthesized via parallel biosynthetic pathways starting from tryptophan and phenylalanine, respectively (Fig. 6) [65], [66]. In these pathways, a transamination is followed by an oxidative decarboxylation [65], [66]. For IAA, this pathway occurs via TAA (TRP AMINOTRANSFERASE of ARABIDOPSIS) and YUCCA family members [67]. For PAA biosynthesis, the transamination step of phenylalanine might not be necessary because phenylpyruvic acid is also a precursor of phenylalanine biosynthesis, but the YUCCA family members do play a role [68]. In line with the enrichment of the indolic and phenylethanoid metabolic classes in late cob and tassel, several members of the *TAA* and the *YUCCA* gene families are highly expressed in the cob and tassel according to the Maize eFP browser [69], making them candidate genes involved in the biosynthesis of IAA and PAA in the respective tissues. In addition to the aspartate amides of IAA and PAA, two hexose derivates of hydroxyindole-3-acetic acid were present in especially late cob and tassel: the hexose ester of 2-hydroxyindole-3-acetic acid (2-hydroxyIAA) was more abundant in the tassel, whereas the hexoside of 5-hydroxyindole-3-acetic acid (5-hydroxyIAA) was prevalent in the late cob. The former compound is likely the glucose ester of 2-hydroxyIAA, and is formed via the oxidation of IAA by DAO (DIOXYGENASE FOR AUXIN OXIDATION) and a subsequent glucosylation by the uridine diphosphate glycosyltransferase UGT74D1 [63]. The tassel also showed high abundances of the hexose esters of PAA and hydroxyphenylacetic acid (HPAA). In maize, 2-hydroxyIAA is an intermediate in the biosynthesis of zeanoside C and, accordingly, the latter compound was highly abundant in the tassel.

Hydroxylation of IAA might yield the aglycone of the 5-hydroxyIAA hexoside, yet such a reaction has not yet been documented to our knowledge. An alternative pathway might start from serotonin (Fig. 6). In plants, serotonin biosynthesis involves the decarboxylation of tryptophan to tryptamine, which is then hydroxylated to serotonin. Despite being undetected in this study, serotonin is known to be synthesized in maize [70]. Furthermore, tryptamine is produced by tryptophan decarboxylase, and a member of the associated gene family has been shown to be highly expressed in the tassel and to a lesser extent in the cob [69]. In addition, a tryptamine 5-hydroxylase, encoded by *CYP71A1*, which converts tryptamine to serotonin, has been characterized in rice [71] and the corresponding enzyme in maize could be annotated when blasting the amino acid sequence via UniProt (https://www.uniprot.org/blast/; 88.6% identity). In human metabolism, once formed, serotonin can be converted to 5-hydroxyindole-3-acetaldehyde and, finally, to 5-hydroxyIAA by the subsequent actions of monoamine oxidase (MAO) and aldehyde dehydrogenase [72]. Some aldehyde dehydrogenase-encoding genes have been shown to be highly expressed in the tassel and to a lesser extent in cobs [69], yet their substrates are still unknown. However, to date, no MAO-encoding gene is known in plants. Consequently, although both the IAA-dependent and -independent pathways are plausible routes for the biosynthesis of 5-hydroxyIAA, gene function analysis remains necessary to pinpoint which biosynthesis path is functional in plants. Nevertheless, knowledge about the presence or absence of specific metabolites in a particular organ is a crucial first step in the construction of putative biosynthetic pathways and in the understanding of the relevance of these pathways in specific organs.

In conclusion, this study shows the benefit of combining different types of CID spectra for structural characterization both via hands-on spectral interpretation and via matching with spectral databases. Despite concluding that spectral matching yielded hits for only 1–2% of the spectra, the use of both MS/MS and MS^n^ spectral elucidation doubled the number of hits with public databases as compared to the use of either type of CID spectrum alone. To allow this spectral metadata analysis, a tool (RDynLib) was created to align chromatograms from different instruments, to analyze the multitude of recorded CID spectra, and to combine this with the analysis of mass-difference networks, such as CSPP networks, for structural characterization. This resulted in the structural characterization of 427 of the 5,420 profiled compounds, of which most had not been described in maize before. Towards the future, the RDynLib package will further benefit from the increase in the number of CID spectra in spectral databases, as well as from the increase in the number of features for which different types of CID spectra are available. The resulting set of characterized compounds revealed the nature of prevailing mass differences among all features, representing enzymatic conversions, and also structural differences between well-known core molecules within specialized metabolism. The latter type of prevailing mass differences is at least partially due to the common nature of the various decorations, e.g., methylation or oxygenation, and the tendency of plants to concatenate specialized metabolite aglycone structures into high-molecular-weight mixed glycosides.

By utilizing different types of CID spectra recorded under different ionization modes, the DynLib database will aid the interpretation of structural features in future comparative metabolome studies in maize (and, likely, other monocots). Besides the spectra of 427 characterized compounds, all recorded unknown spectra are available via an online webtool (https://bioit3.irc.ugent.be/dynlib/). Using this database, the authors intend to continue their own structural elucidation efforts, but welcome proposed structures, which can be uploaded via the webtool associated with the DynLib database, via the metabolomics community.

## CRediT authorship contribution statement

**Sandrien Desmet:** Conceptualization, Formal analysis, Methodology, Software, Writing - original draft, Writing - review & editing. **Yvan Saeys:** Conceptualization, Software, Writing - review & editing. **Kevin Verstaen:** Software, Writing - review & editing. **Rebecca Dauwe:** Conceptualization, Methodology, Writing - review & editing. **Hoon Kim:** Formal analysis, Writing - review & editing. **Claudiu Niculaes:** Formal analysis, Methodology, Writing - review & editing. **Atsushi Fukushima:** Writing - review & editing. **Geert Goeminne:** Formal analysis, Writing - review & editing. **Ruben Vanholme:** Conceptualization, Methodology, Writing - review & editing. **John Ralph:** Formal analysis, Writing - review & editing. **Wout Boerjan:** Conceptualization, Methodology, Writing - original draft, Writing - review & editing. **Kris Morreel:** Conceptualization, Formal analysis, Methodology, Software, Writing - original draft, Writing - review & editing.
