# Supplementary material for: Maize specialized metabolome networks reveal organ-preferential mixed glycosides
Source: Comput Struct Biotechnol J. 2021 Jan 26;19:1127–44. doi: 10.1016/j.csbj.2021.01.004 (PMC7890092; doi:10.1016/j.csbj.2021.01.004)
Supplement: Supplementary data 1 [file mmc1.pdf]

SUPPLEMENTAL TEXT

Compound purification and NMR experiments

Maize plants (genotype B104) were grown in a greenhouse (day 25 °C, night 23 °C, 16h/8h day/night rhythm), until they reached a height of approximately 1 m. The internode below the cob (21 plants) was harvested and homogenized in liquid nitrogen. Following extraction of all homogenized tissues in 200 mL of methanol, the supernatant was evaporated with a Rotavapor RII (Buchi, Flawil, Switzerland). Evaporation occurred using a 200 mbar vacuum applied by a Vacuum controller V-855 (Buchi) coupled to a Vacuum pump V-700 (Buchi), and a temperature gradient (hot water bath, condenser and condensate-collecting flask were kept at 40, 20 and 0 ˚ C, respectively). After evaporation, the residue was dissolved in 20 mL cyclohexane/MilliQ water (1/1, v/v). The aqueous phase was then separated on a Reveleris X2 Flash chromatography system (Grace, Columbia, MD) using a Reveleris® C18 12g reverse-phase column (Grace). A gradient was run from 99% solvent A (solvent A: MilliQ water/methanol/formic acid, 98.9/1/0.1, v/v/v) to 55% solvent B (solvent B: methanol) in 30 min at 30 mL/min, collecting 10-mL fractions. Each fraction was profiled via the UHPLC-IT-FT-ICR-MS approach described in Methods. Fractions containing unknown compounds of interest were further purified via High Performance LC (HPLC) using a 625LC pump (Waters) coupled to a 996 Photodiode Array Detector (Waters). Two subsequent separations were performed using a gradient of 99% solvent A (solvent A: MilliQ water/acetonitrile/acetic acid, 98.9/1/0.1, v/v/v) to 100% solvent B (solvent B: acetonitrile/MilliQ water/acetic acid, 98.9/1/0.1, v/v/v) in 30 min at 6 mL/min. For the first and second separation, a Luna C18(2) (10 x 250 mm, 10 µ; Phenomenex, Torrance, CA) and a Platinum EPS C18 (10 x 250 mm, 10 µ; Grace) column were employed. 3-mL fractions were collected with a BioFrac™ Fraction Collector (Bio-Rad, Hercules, CA). Based on the interpretation of the MS data from UHPLC-IT-FT-ICR-MS profiling, the presence of DIMBOA glucoside and vanilloyl hexose in two fractions could be authenticated by NMR. Furthermore, TRIBOA glucoside was identified in the vanilloyl hexose-containing fraction. As TRIBOA glucoside was not detected via the MS platforms, this identified compound was excluded from the set of 427 compounds that were all, at least initially, structurally elucidated based on MS data.

NMR spectra for the isolated compounds were authenticated by the normal range of 1D (^{1}H and ^{13}C) and standard 2D (COSY, HSQC, and HMBC) experiments on a Bruker Biospin (Billerica, MA) Avance III 600 MHz spectrometer equipped with a 1.7-mm TCI ^{1}H/^{13}C/^{15}N cryoprobe with inverse geometry. All experiments were performed in methanol-d$_4$. The central methanol solvent peak was used as the internal reference (δ_C 49.0, δ_H 3.30 ppm).

DIMBOA glucoside (2,4-dihydroxy-7-methoxy-1,4-benzoxazin-3-one glucoside): ^{1}H NMR (methanol-d$_4$): δ 7.25 (d, *J* = 8.8 Hz, 1H, 5), 6.75 (d, *J* = 2.4 Hz, 1H, 8), 6.69 (dd, *J* = 8.8, 2.4 Hz, 1H, 6), 5.90 (s, 1H, 2), 4.67 (d, *J* = 8.0 Hz, 1H, 1′), 3.85 (m, 1H, 6′A), 3.77 (s, 3H, OMe), 3.67 (dd, *J* = 11.8, 5.0 Hz, 1H, 6′B), 3.38-3.15 (m, 4H, 2′,3′,4′ and 5′); ^{13}C NMR (methanol-d$_4$): δ 158.7 (7), 157.5 (3), 143.3 (9), 122.8 (10), 115.1 (5), 109.8 (6), 104.7 (8), 103.9 (1′), 98.2 (2), 78.5 (4′), 77.8 (3′), 74.8 (2′), 71.0 (5′), 62.5 (6′), 56.2 (OMe).

TRIBOA glucoside (2,4,7- trihydroxy-2H-1,4-benzoxazin-3-(4H)-one glucoside): ^{1}H NMR (methanol-d$_4$): δ 6.74 (d, *J* = 8.5 Hz, 1H, 5), 6.53 (d, *J* = 2.0 Hz, 1H, 8), 6.45 (dd, *J* = 8.5, 2.0 Hz, 1H, 6), 5.70 (s, 1H, 2), 4.65 (s, 1H, 1′), 3.85 (d, *J* = 11.8 Hz, 1H, 6′A), 3.69 (d, *J* = 12.8 Hz, 1H, 6′B), 3.40-3.15 (m, 4H, 2′,3′,4′ and 5′); ^{13}C NMR (methanol-d$_4$): δ 117.0 (5), 110.7 (6), 105.9 (8), 103.7 (1′), 96.3 (2), 78.2 (4′), 77.6 (3′), 74.6 (2′), 70.7 (5′), 62.1 (6′).

1-*O*-Vanilloyl-β-D-glucose: ^{1}H NMR (methanol-d$_4$): δ 7.63 (br d, *J* = 8.2 Hz, 1H, 6), 7.60 (br s, 1H, 2), 6.85 (d, *J* = 8.2, 1H, 5), 5.67 (d, *J* = 7.4 Hz, 1H, 1′), 3.90 (s, 3H, OMe), 3.85 (d, *J* = 11.8 Hz, 1H, 6′A), 3.69 (d, *J* = 12.3 Hz, 1H, 6′B), 3.50-3.38 (m, 4H, 2′,3′,4′ and 5′); ^{13}C NMR (methanol-d$_4$): δ 125.5 (6), 115.7 (5), 113.5 (2), 95.8 (1′), 78.5, 77.8, 73.8, 70.8 (2′, 3′, 4′ and 5′), 56.1 (OMe).

LC-MS data processing

Chromatograms obtained via the QTOF were processed with Progenesis QI software version 2.1 (Waters Corporation). The raw data were imported in this software using a filter strength of 1. A reference chromatogram was manually chosen for the alignment procedure and additional vectors were added in chromatographic regions that were not well aligned. Peak picking was based on all runs with a sensitivity set on 'automatic' (value = 5). The normalization was set on 'external standards' and was based on the dry weight of the samples (Morreel et al., 2006). In total, 8,514 and 7,647 features were integrated and aligned across all chromatograms in negative and positive ionization mode. All recorded MS/MS spectra were exported to an msp file by Progenesis. The processed data (csv file) and the MSMS.msp file were added to a dedicated ~/Experimenten/Exp* directory in the considered DynLib subDB (Supplemental Figure 2). The csv and msp files were subsequently converted to the nodes.txt and MSMS.txt files. Within the MSMS.txt file, all MS/MS spectra were unique.

FT chromatograms were sliced with RecalOffline vs. 2.0.2.0614 (Thermo Electron Corporation, Waltham, MA) into three data files containing the full MS, MS^2, and MS^3 spectra. The full MS data files and the MS^2 and MS^3 files were exported, using the File Converter function in Xcalibur 2.0 SR2 (Thermo Electron Corporation), to cdf and txt files, respectively. For each of the individual FT chromatograms, the MS2.txt and MS3.txt files were stored in their respective ~/Experimenten/Exp* directories in the considered DynLib subDB (Supplemental Figure 2). Within the MS2.txt and MS3.txt files, all MS^2 and MS^3 spectra were unique. The full MS-containing cdf files were processed via the XCMS package in R (Smith et al., 2006) using previously published parameter values for the different functions (Morreel et al., 2014). Processing all chromatograms yielded 23,005 and 9,373 features in the negative and positive ionization mode. For each ionization mode, the resulting xcms.tsv file was then stored in all ~/Experimenten/Exp* directories corresponding to each of the individual FT chromatograms in the considered DynLib subDB. The xcms.tsv file was subjected to the *XCMSgrouping()* function present within the RDynLib package (see further in this section and the file 'RDynLib tools' available at https://floppy.psb.ugent.be/index.php/s/O9z6mU8IiAlWGbT). This yielded the xcms2.txt and the nodes.txt files that were both copied to all considered ~/Experimenten/Exp* directories. It should be stressed that each FT chromatogram was associated with its own ~/Experimenten/Exp* directory, whereas, in the case of QTOF analyses, an overall ~/Experimenten/Exp* directory was created to contain chromatographic data from all organs and genotypes. Entering FT chromatograms as independent subDB experiments allowed alignment of the two subDB experiments representing the same organ and genotype but of which the chromatograms were based on recording different sets of MS^3 spectra (see above).

DynLib database construction

To enter MS data into the DynLib database (upgrading the csv files in the 'CSV' folder; see Supplemental Figure 2), a perl script called add_experiment.pl (which itself depends on the match_experiment.pl script and on the extract_dataMS2.pl and fill_database.pl scripts in the case of the FTMS-based and QTOF-based subDBs, respectively) was written that takes the nodes.txt, and either the MS2.txt and MS3.txt or an MSMS.txt as input files. In addition, an experiment.txt file was necessary (Supplemental Figure 2). For each feature in the nodes.txt file that had a CID spectrum, the best matching DynLib subDB spectrum (based on a dot product of the product ions and losses in common between both spectra; see Morreel et al., 2014) was given in the matches.txt output file. If the matching DynLib spectrum had a COMPNAME (Supplemental Figure 2), then the COMPNAME was preceded by the matching score and entered into the COMPNAME field of the newly entered feature via the RDynLib-associated *AddingNames()* function (see the file 'RDynLib tools' available at https://floppy.psb.ugent.be/index.php/s/O9z6mU8IiAlWGbT). Both

the perl script to add CID spectra from any new experiment, as well as the database itself, are available on https://floppy.psb.ugent.be/index.php/s/O9z6mU8IiAlWGbT. In addition, a webtool of the DynLib database was constructed using Laravel (conversion of csv files to MySQL) and Vue.

RDynLib construction and application

The RDynLib package was constructed using R version 3.4.2 (R Core Team, 2017). The RDynLib package also requires the R packages igraph (Csardi and Nepusz, 2006), RColorBrewer (Neuwirth, 2014), and rcdk (Guha and Cherto, 2017). The different functions (see the file 'RDynLib tools' available at https://floppy.psb.ugent.be/index.php/s/O9z6mU8IiAlWGbT for an overview) tackle either the (i) DynLib data maintenance or (ii) CID spectral elucidation. The following RDynLib functions were applied to relate all subDB experiments present within the DynLib subDBs using the default parameter values. In the case of an FT experiment and following the addition of all MS data to the DynLib database, the RDynLib-associated *XCMSgrouping2()* function was run, annotating features representing in-source fragments and adducts. For a particular ionization mode, the LC-MS data within each subDB experiment in the FTMS-based DynLib subDB were then aligned with those of the QTOF subDB experiment in the respective DynLib subDB via the *Aligning_FT_QTOF()* function. For the negative and positive ionization-based DynLib subDBs of a particular LC-MS platform, LC-MS data within the corresponding subDB experiments were aligned via the *AligningFT_neg_pos()* function. In the case of FT analyses, two chromatograms were recorded for each sample, each chromatogram containing data from different MS^3 scan events (see above). Within a particular subDB, subDB experiments associated with the same sample were aligned via the *Aligning_Exp()* function. Within each DynLib subDB experiment, CSPPs (Morreel et al., 2014) were pinpointed via the *MultExp_CSPP()* function, whereas pairs of features with similar CID spectra and their mass differences were computed with the *MultExp_GNPS()* function. Within the same FTMS-based DynLib subDB experiment, the names (COMPNAME; Supplementary data 2 - Supplemental Data Set 1) of structurally characterized compounds were copied via the *TablewiseIdent()* function to other entries that represented the same compound but belonged to different subDB experiments. Between the DynLib subDBs, names (COMPNAME) were transferred to entries that were linked to the same compound using the *SubDBNameTransfer()* function.

Structural elucidation of CID spectra

CID spectral elucidation started by matching all spectra against external MS spectral databases (http://mona.fiehnlab.ucdavis.edu/downloads). The external MS spectral databases were converted into the DynLib-based database structure using python version 3.6.5. Subsequently, all CID spectra within the MS2spectra.txt files of the different DynLib subDB experiments were compared against those in the external MS spectral database (DynLib format). An identical MS spectral match was based on the dot product using both a reverse- and forward-search algorithm written in python. All python scripts are available on https://floppy.psb.ugent.be/index.php/s/O9z6mU8IiAlWGbT.

Next, CID spectra were structurally elucidated using the DynLib database in conjunction with the RDynLib package, predominantly focusing on the negative ionization CID spectra (Supplemental Text, *Compound class-specific gas-phase fragmentations*; Supplemental Figures 10, 14-23). Whenever possible, candidate structures were taken from compound databases such as the PubChem (Kim et al., 2016), the KNApSAcK metabolite (Nakamura et al., 2014), and the FooDB (http://foodb.ca) databases. When available, the latter database also offers access to CID spectra. In the absence of database CID spectra, MetFrag (Wolf et al., 2010) and CSI:FingerID (Dührkop et al., 2015) were applied to reveal possible candidate structures using the PubChem database. Alternatively, the CID spectrum of a proposed structure was predicted via CFM-ID (Allen et al., 2015). Two compounds were authenticated by NMR upon HPLC-based purification to

confidently assign the structures for DIMBOA glucoside and 1-*O*-vanilloyl-β-D-glucose (see above, *Compound purification and NMR experiments*). In the fraction of the last compound, TRIBOA glucoside was also present based on NMR. Other structures were authenticated by an identical match of their CID spectra and retention times to those of a synthesized or purchased compound (see Supplementary data - Supplemental Data Set 1 for details). For a number of compounds, structural moieties could be identified by matching the corresponding MS^3 spectrum to the MS^2 spectrum of standard compounds (see Supplementary data - Supplemental Data Set 1). Verifying the presence of the characterized structures in PubChem (https://pubchem.ncbi.nlm.nih.gov/) was performed via the ChemmineR package (Cao et al., 2008) in R version 3.4.2 (R Core Team, 2017) using an adapted version of the *pubchemSmilesSearch()* function. The presence of the structures in maize was checked using the FooDB and CornCyc databases from which the FooDB.csv (version of June 29, 2017) and the corncyc_compounds.20180702 files were downloaded. For database matching, the chemical formula and the most structurally similar isomer were determined using the SMILES representation of the structure. Computing the chemical formula occurred with the following functions: *parse.smiles()[[1]]*, *convert.implicit.to.explicit(),* and *get.mol2formula()@string*. The most similar isomer was picked using *lapply(get.fingerprint, type='circular')* and *fingerprint::fp.sim.matrix(method='tanimoto')[-1,1]*.

Adding MS spectral data to the DynLib database

The CID spectral data sets of the different maize organs and genotypes that were recorded with each of the different LC-MS platforms were added as a new subDB experiment within their LC-MS platform-corresponding subDB of the DynLib database, called the 'FTMS_neg', 'FTMS_pos', 'QTOF_neg', and 'QTOF_pos' subDBs (Supplemental Figure 2). A subDB experiment might contain data from several aligned chromatograms in which precursor ion features are represented by median m/z and retention time values, or it could store data from only one chromatogram. Each DynLib subDB includes the 'CSV' directory with csv files containing information on the experimental settings, the sample type, and the MS data of all profiled compounds. The 'CSV_add' directory stores information about potential "substrate–product" associations (based on a fixed set of biotransformations) between features within a subDB experiment (see further), and connections between LC-MS data obtained from the same sample but present in different subDB experiments within a particular subDB. The original chromatographic data for each subDB experiment is retained within the 'Experimenten' directory. In addition to the four DynLib subDBs, a fifth section within the DynLib database, called 'DynLib SubDB alignment', contains information concerning connections between subDB experiments belonging to different subDBs that contain LC-MS data from the same sample. Also, it stores information on putative m/z ion–structure and mass difference/neutral loss–structure associations.

Supplemental Table 1 displays the number of MS^n and MS/MS spectra for each organ and each genotype present in the DynLib subDBs. Different processing approaches were used for the FT and the QTOF raw data sets (see Methods). For the FT raw data sets, a list of MS^n spectra and, thus, a subDB experiment, was generated for each organ and genotype separately. For the QTOF raw data sets, in contrast, the MS/MS spectral list encompassed all organs and genotypes and, thus, yielded only one subDB experiment for each ionization mode. Consequently, if a particular compound was present in different organs and/or genotypes, multiple MS^2 spectra of the corresponding feature were available in the FT-based subDBs, whereas each MS/MS spectrum was unique in the QTOF-based subDBs. Per organ and genotype, the average numbers of features having MS^n spectra in the FTMS_neg (589 features) and FTMS_pos (317 features) subDBs were only 52% and 35%, respectively, of those having MS/MS spectra in the QTOF_neg (1135 features) and QTOF_pos (892 features) subDBs. The lower number of MS^n spectral trees retrieved via FT was mainly caused by the recording of MS^3 spectra in addition to MS^2 spectra, requiring more time than the recording of a single MS/MS spectrum. In total, 4208 and 1785 unique MS^n spectra (for

readability referred to as ‘MS^n spectra’ instead of ‘unique MS^n spectra’ in the manuscript), and 2665 and 1816 MS/MS spectra were entered into the FTMS_neg and FTMS_pos, and into the QTOF_neg and QTOF_pos subDBs, respectively. Starting from the number of unique CID spectra, the number of maize compounds represented in the DynLib database was computed by taking into account that (i) CID spectra of different MS^1 ions of the same compound are sometimes archived in a DynLib subDB, and (ii) the alignment success rate between the different DynLib subDB.

Aligning SubDB experiments using RDynLib

Features representing the same ions in different subDB experiments of the DynLib database were aligned using an in-house-developed R package called RDynLib (the RDynLib functions are described in more detail in the file ‘RDynLib tools’ available at https://floppy.psb.ugent.be/index.php/s/O9z6mU8IiAlWGbT). Whenever a subDB experiment is based on one chromatogram only, it is aligned to another subDB experiment using the recorded m/z and retention time values. In case a subDB experiment is constructed from multiple chromatograms, its alignment to another subDB experiment involves the median m/z and retention time values, which are computed based on the recorded values across all chromatograms. Because the alignment occurs between subDB experiments rather than chromatograms, only features for which CID spectra are available are included.
The alignment of subDB experiments involves retention time modeling, which can be improved by selecting reference features that are more or less evenly distributed throughout the chromatograms; features that show highly similar CID spectra between subDB experiments of the DynLib database can be readily taken as reference feature pairs (RFPs, Supplemental Figure 3, step 2). However, careful selection is needed for finding RFPs. Even in the case of selecting RFPs based on identical CID spectra (e.g., between subDB experiments belonging to the same DynLib subDB), retention time regression (Supplemental Figure 3, step 3) will be biased whenever isomer mismatching occurs between the subDB experiments. For example, many *cis*/*trans* isomers in phenylpropanoid metabolism elute closely and show identical CID spectra. Such false-positive RFPs will occur even more frequently whenever subDB experiments that belong to different subDBs are matched. This happens because the different DynLib subDBs represent different types of CID spectra so that a perfect match is no longer feasible. Indeed, the MS/MS spectrum of a compound will contain more low-mass m/z product ions and will show different ion abundances as compared to its MS^2 spectrum. Furthermore, only some of the fragmentations occurring in negative and positive ionization mode for a particular compound are similar (taking into account that the resulting m/z product ions are shifted by 2.014 Da, the mass difference between the positive and negative pseudo-molecular ion). To avoid such false-positive RFPs, a prior so-called ‘local retention time alignment’ step, only based on m/z value and retention time, was included in the overall alignment procedure (Supplemental Figure 3, step 1). A local retention time alignment between two different DynLib subDB experiments is performed by searching the second subDB experiment for a local region of adjacent features picked from the first, reference subDB experiment. If distinct isomers are present among the features in the local region, all possible local alignments are explored, and the match with the lowest total retention time shift is selected (see below, *Local retention time alignment*; Supplemental Figure 4). This ‘local region’ matching happens iteratively for all features of the reference subDB experiment.
Overall, the full alignment procedure consists of (i) generating a list of putative RFPs via local retention time alignment, (ii) filtering this RFP list by CID spectral matching, (iii) performing retention time regression on the retained RFPs, (iv) using the so-obtained model to correct the retention times of the features present in the second subDB experiment, and (v) re-alignment of features in both subDB experiments based on m/z value, retention time difference (based on the corrected retention time for the features in the second subDB experiment) and CID spectral similarity. The so-obtained feature (and CID

spectral) associations are stored in the 'DynLib SubDB alignment' section of the DynLib database (Supplemental Figure 2).

Less stringent CID spectral matching parameters, and a slightly different alignment approach, were implemented in the alignment of subDB experiments belonging to different DynLib subDBs than when both subDB experiments were present in the same subDB (see below, *CID spectral matching and regression*). In the manuscript, the numbers and frequencies of aligned features between the different subDBs are based on matching unique MS^n spectra (see above).

Local retention time alignment

The local retention time alignment procedure (Supplemental Figure 4) occurs iteratively from the start to the end of both subDB experiments (i.e., chromatograms), each time taking a region of, e.g., five neighboring features in the reference subDB experiment (e.g., from the DynLib FTMS_neg sub-database, representing a LC-FT-MS chromatogram) and tracing the analogous region in the second subDB experiment (e.g., from the DynLib QTOF_neg sub-database, representing a LC-QTOF-MS chromatogram). All possible retention time differences for each of the five LC-FT-MS features with all possible isomeric LC-QTOF-MS features are calculated. For each combination of LC-FT-MS / LC-QTOF-MS feature pairs, the standard deviation on the retention time differences is computed and the combination that yields the lowest standard deviation is selected as the most likely. For simplicity, only the m/z 583 feature from the selected region is aligned and retained in Supplemental Figure 4. The next iteration occurs via a moving window principle in which the local region of five adjacent features in the reference chromatogram is shifted to the right, including a new feature with a slightly higher retention time and dropping the feature with the lowest retention time. Except at both ends of the LC-FT-MS chromatograms, each LC-FT-MS feature will therefore enter such a local retention time alignment iteration five times. In the case of a close correspondence between a LC-FT-MS and a LC-QTOF-MS region, the same feature associations will be repetitively picked up during subsequent iterations, providing an improved regression modeling later onwards. Alternatively, in the absence of a good regional similarity between the LC-FT-MS and LC-QTOF-MS chromatograms, different associations for an involved LC-FT-MS feature might be obtained during subsequent iterations down-scaling the importance of this feature for regression modeling, yet also introducing false-positive associations. These false-positive associations are expected to occur randomly, having either a positive or a negative retention time difference between the LC-FT-MS and LC-QTOF-MS chromatograms. This will introduce additional variance, but is not expected to yield a more biased model. In addition, such false-positive associations will be largely removed from the list of putative reference feature pairs (RFPs) via collision-induced dissociation (CID) spectral matching.

CID spectral matching and regression

The most stringent matching is achieved when associating two subDB experiments within the same DynLib sub-database as identical CID spectra are expected. Here, CID spectral matching demands that all product ions of one CID spectrum are traced in the other CID spectrum in either a forward or a reverse match with a minimal dot product of 0.9 by default. Less stringent criteria are necessary in the case of CID spectral matching between subDB experiments belonging to different DynLib sub-databases: when aligning either MS^n versus MS/MS data or negative-ionization-based versus positive-ionization-based CID spectral data, the spectral matching criterion involves the presence of a minimum number of product ions with the same m/z value in common. In the case of aligning negative-ionization-based versus positive-ionization-based CID spectral data, such a 'count' criterion necessitates taking a mass difference of 2.014 Da into account between the product ions in both types of spectra owing to the difference in charge. The latter procedure is valid as positive- and negative-ionization CID spectra of the same compound reflect at least in part the

same charge-remote gas-phase fragmentation channels (Demarque et al., 2016). By default, the 'count' threshold is set at 0.6 (60% of the MS^2 product ions should be traced in the MS/MS spectrum) and 0.2 (20% of the negative product ions should be traced in the positive-ionization spectrum) in the case of MS^n versus MS/MS spectral matching and negative-ionization-based versus positive-ionization-based CID spectral matching, respectively. These default 'count' thresholds are rather low, but avoid rejecting too many true-positive RFPs. On the other hand, combining the default 'count' thresholds for CID spectral matching with a prior local retention time alignment, aids preventing the inclusion of false-positive RFPs. In addition, RFPs of which the retention time difference exceeds a preset threshold are removed. The latter is necessary as structural isomers might readily lead to false positive RFPs. Using a retention time difference filter is not carried out in the case of aligning subDB experiments from the same sub-database. In this case, as identical CID spectra are a prerequisite for a RFP, only *cis/trans* isomers and perhaps diastereomers might lead to false-positive RFPs; such isomers elute in close proximity to each other.
The list of so-obtained RFPs is then used for regression modeling. Robust regression (Iteratively Weighed Least Squares-based, *rlm* function in R) outperformed classical regression (*lm* function in R) for several test sets (data not shown). In the case of associating positive- and negative-ionization CID spectra, a linear regression approach was performed as both chromatograms were recorded on the same instrument, in the same time period and using the same chromatographic conditions, and is necessary as the CID spectral matching is not stringent. When associating subDB experiments between MS^n and MS/MS-based DynLib sub-databases, a hyperbolic retention time relationship was often evident, hence supporting a model based on piecewise regression including at most two knots. A more elaborate modeling approach could have been performed but was not necessary for the structural characterization-based purpose of the current version of the DynLib database. This is because all CID spectra belonging to a particular compound are being visualized upon spectral elucidation, which readily allows the detection of possible mismatches. Piecewise regression is also implemented when associating subDB experiments within the same DynLib sub-database. Although the same chromatographic conditions were applied in this study, including piecewise regression would allow retention time alignment between samples analyzed under slightly different chromatographic conditions in the future. This is possible as an identical rather than a similar CID spectral matching is requested, enabling relaxing regression from a linear to a piecewise linear model. Once obtained, the regression model is then used for retention time correction. In the case of aligning subDB experiments belonging to the same sub-database, this retention time correction is employed to eliminate false-positive RFPs (as generated by, e.g., closely eluting *cis/trans* isomers or diastereomers); the remaining RFPs are subsequently stored. However, in the case of aligning subDB experiments belonging to different sub-databases, filtering the RFP list based on the maximum retention time difference threshold (see above) might have eliminated too many true-positive RFPs. Therefore, a new alignment of the features between both experiments is necessary, which is based on their m/z values, corrected retention times and CID spectral similarity. Three full alignment procedures were programmed in RDynLib, i.e., to associate experiments belonging to the same DynLib sub-database [*Aligning_Exp()*], to associate MS^n-based versus MS/MS-based experiments [*Aligning_FT_QTOF()*], or to associate negative ion-based versus positive ion-based CID spectra [*AligningFT_neg_pos()*].

Structural characterization tools in RDynLib

In addition to performing alignment between different DynLib subDB experiments, various tools are included in RDynLib to facilitate structural characterization (the RDynLib functions are described in more detail in the file 'RDynLib tools' available at https://floppy.psb.ugent.be/index.php/s/O9z6mU8IiAlWGbT). Some tools are specific for either the MS^n or the MS/MS spectra. Starting from a feature of interest for which, e.g., the MS/MS spectrum can be displayed, the subDB IDs (referred to as COMPID) are given for associated CID spectra present in other DynLib subDBs. From here, different approaches can be taken

towards a hands-on structural characterization (Supplemental Figure 5). Important RDynLib functions for the various tools are indicated below in italic font.

QTOF-derived MS/MS spectra are plotted via the *MSMSspecplot()* function. The m/z values of the MS/MS product ions are sufficiently accurate to allow 'QTOF-based neutral loss analysis' (Supplemental Figure 6). Candidate chemical formulae and structures can be generated for the product ions and the corresponding neutral losses in the MS/MS spectrum via the functions *ProdIonMatch()* and *NeutLossMatch()*, respectively. These functions take lists of candidate product ions and candidate neutral losses, respectively, as input; both are present in the 'DynLib SubDB alignment' directory of the DynLib database and can be upgraded by the user. In subsequent steps, any product ion can be selected from which the mass differences to other product ions are computed, accompanied by candidate formulae and structures.

Complementary product ions, in which the sum of the masses of the corresponding neutral molecules equals the mass of the neutral molecule of the precursor ion, are returned via the *ComplemIons()* function. Such complementary product ions give insight into the chemical structure of the compounds, because they might, e.g., point to the presence of an ester linkage.

FT-derived MS^n spectra are plotted via the *MSnspecplot()* function (Supplemental Figure 7). Here, MS^3 data from the same precursor ion (same retention time, m/z value and MS^2 spectrum) that were recorded in different subDB experiments (e.g., when the subDB experiments contain different LC-MS chromatograms obtained from the same sample but using different settings for MS^3 spectrum recording) can be displayed together. This allows implementing a large number of MS^3 spectra in the structural characterization pipeline.

Via the *Find_isomers()* function, all isomers present in the QTOF and FT profiles are retrieved. Subsequently, their CID spectra can be visualized and compared to that of the selected ion via the *dynamCIDspecplot()* function (Supplemental Figure 8). This allows checking whether the correct features were aligned between both instrument platforms, but also enables taking the CID spectra and retention times of other isomers into account for structural characterization.

The FT-based MS^1 spectrum is useful for structural characterization because it provides information on the isotope pattern, adduct abundances, and in-source fragments. (i) The isotope pattern readily aids resolving the chemical formula because the high resolution of the FT allows a distinction between isotopes of the pseudo-molecular ion, e.g., between the $^{13}C_2$ (+2.0067 Da) and the ^{34}S (+1.9958 Da) isotopes. (ii) The relative abundance of an adduct versus that of the pseudo-molecular ion might hint at the absence of particular functional groups. For example, if the formic acid adduct is much more abundant than the pseudo-molecular ion in negative ionization mode, phenolic or carboxylic acid functions are less likely to be present. (iii) The detection of in-source fragments suggests the presence of labile bonds such as acetal or ester bonds. The MS^1 spectrum of a particular COMPID can be plotted via the *MS1specplot()* function (Supplemental Figure 9). For this purpose, the full FT spectrum is retrieved from the processed LC-MS data, which is stored within the 'Experimenten' directory in either the FTMS_neg or FTMS_pos subDB. The MS^1 spectrum is constructed by features in the full FT spectrum with the same retention time as that of the COMPID-related feature. In addition, whenever multiple chromatograms are available in the subDB experiment, the Pearson correlation coefficient is computed between the intensities of each feature and those of the COMPID-related feature across the chromatograms. Only features of which the intensity correlates with that of the COMPID of interest (the default correlation cut-off is set at 0.8), are considered part of the MS^1 spectrum.

RDynLib allows for an in-depth 'CID spectral matching' (Supplemental Figure 10). Via the *MultMatchCID()* function, MS^2 or MS/MS spectra of interest can be matched against the DynLib database MS^2 or MS/MS spectra, respectively. In addition, the MS^3 spectra can be compared to either the DynLib database MS^2 [via *MultMatchMS3MS2()*] or MS^3 spectra [via *MultMatchMS3()*]. The inclusion of MS^3 spectral matching has the potential to identify a structural moiety of the feature of interest whenever the MS^3 spectrum is

identical to a known database MS^2 spectrum or it might hint to a common candidate substructure if it matches the MS^3 spectrum of a known database entry.
The CID spectra in a subDB experiment that contain a requested CID spectral motif can be displayed via the *SearchFingerprint()* function. Such a CID spectral motif consists of a restricted number of product ions that are considered to represent a particular structural moiety (Ye et al., 2016; van der Hooft et al., 2016). Based on a set of CID spectra, a CID spectral motif from the common product ions can be generated via the *CIDfingerprint()* function.
In addition to MS spectral metadata analysis tools, RDynLib also allows structural characterization via propagation through mass-difference networks via the *Overall.net()* function. Two complementary approaches are applied in this mass-difference analysis (Supplemental Figure 11). The first is based on candidate substrate–product pairs (CSPPs; Morreel et al., 2014) and the second on a global natural product social molecular networking (GNPS)-like strategy (Watrous et al., 2012). The CSPP algorithm first annotates a pair of features as a CSPP whenever the mass difference and the elution order correspond to those expected for known biochemical reactions. Thirty-four different CSPP conversion types are taken into account (see cspp.txt file within the CSV_add directory of the considered subDB). Then, this algorithm computes the CID spectral similarity between both features. In contrast, the GNPS-like algorithm annotates pairs of features based on CID spectral similarity first and then computes the mass difference. The two approaches are complementary. Whereas the CSPP approach will miss biochemically related features whenever the biotransformation is not taken into account by the algorithm (e.g., in the case of infrequently occurring biotransformations), the GNPS-like approach will fail to annotate biochemically related features that have dissimilar CID spectra. Indeed, whereas CID spectral similarity indicates similar molecular structures, the reverse is not necessarily true (Rojas-Cherto et al., 2012; Rasche et al., 2012). All CID spectra associated with any of the two types of mass-difference networks can be plotted via the *MS2series()* function, designed to visualize MS/MS or MS^2 spectra from multiple COMPIDs.

Compound class-specific gas-phase fragmentations

For the gas-phase fragmentations described below, often pericyclic and stepwise reaction pathways can be simultaneously proposed. Moreover, a dissociation might not involve the charge site as the deposited internal energy during CID is spread across all vibrational energy levels of the ion in agreement with Quasi-Equilibrium Theory (QET). Nevertheless, all gas-phase fragmentations in the remainder of this text describe charge-driven reactions unless otherwise mentioned. All of the described compounds are among the compounds listed in Supplementary data - Supplemental Data Set 1; their CID spectra can be consulted via the DynLib database (using either RDynLib or the web-based version of the database).

Benzoxazinoids. CID spectra for benzoxazinoids obtained in either positive- (Walker et al., 2011) or negative-ionization (Hanhineva et al., 2011) mode have been published. In addition, tentative gas-phase fragmentation pathways for the aglycone anions were described by Bonnington et al. (2003), which was especially focused on the 46-Da loss (-CH_2O_2) from the anions of DIBOA and DIMBOA yielding the product ions at m/z 134 and 164, respectively. The fragmentation was explained to occur via oxygen loss from the hydroxylamine function and formaldehyde loss from the acetal function, leading to a benzoxazolinone anion. Here, the glucosides of DIBOA and DIMBOA were lost as either anhydroglucose or glucose (Supplemental Figure 16). Both the aglycone and the dehydrated aglycone product ions of DIBOA and DIMBOA glucoside were able to dissociate further (upon MS^3 fragmentation) to the ions at m/z 134 and 164. This indicates that the 46-Da loss observed during CID of benzoxazinoid hydroxamic acids is due to water loss from the acetal group combined with carbon monoxide loss with formation of the benzoxazolinone anion. The gas-phase fragmentation of benzoxazinoid lactams is illustrated for HBOA glucoside (Supplemental Figure 16 and 17). The HBOA glucoside anion (m/z 326) dissociation starts with

the loss of anhydrohexose, likely via a charge-remote process, yielding the product ion at m/z 164. A retro aldol cleavage of the acetal bond opens the ring (similar to the ring opening of a reducing sugar; Carroll et al., 1995; Mulroney et al., 1999), which is then followed by the loss of 46 Da yielding the ion at m/z 118. However, in contrast with the loss of 46 Da upon CID of benzoxazinoid hydroxamic acids, here, it is due to expelling a formic acid neutral via an ion-neutral complex intermediate. Competing with this fragmentation channel is a reaction sequence of two subsequent carbon monoxide (CO) losses leading to the product ions at m/z 136 and 108. The latter CO losses were also described by Bonnington et al. (2003). The first CO loss in the fragmentation process of the HBOA glucoside anion assumes that the acyl hydrogen is abstracted by the phenoxide anion (Supplemental Figure 17). A proton transfer involving the acyl hydrogen of an aldehyde anion has been previously described as a gas-phase fragmentation based on Density Functional Theory (DFT) computations (Kanawati et al., 2008), but has also been experimentally supported upon CID in the chemical ionization (CI)-MS analysis of *trans*-2-hexenal, in which acyl hydrogen abstraction was feasible owing to the presence of an adjacent double bond (Custer et al., 2003). Here, the presence of two neighboring carbonyl functions in HBOA might also facilitate acyl hydrogen abstraction. However, CO loss from the aldehyde via a Norrish type 1-like extrusion can also be envisaged.

The (2*R*) and (2*S*) epimers of the benzoxazinoids arise via oxo-cyclo tautomerism of the aglycone. They can be distinguished as the (2*S*) epimer elutes before the (2*R*) epimer on a reverse-phase column (Wouters et al., 2014). The product ion arising as a result of a 46-Da loss from the aglycone ion, is more prominent in the CID spectrum of the (2*R*) epimer than in that of the (2*S*) epimer.

Upon hydrolysis of the glycosidic bond in the benzoxazinoid, the resulting hemiacetal aglycone is an electrophilic species that degrades via different pathways (Wouters et al., 2016). In addition to benzoxazolinone formation, coupling might occur with amines and thiols. This leads to the structural characterization of, e.g., *N*-hydroxy-*N*-(2-hydroxyphenyl)-2-iminoethanimidamide.

Indolics. When an alkyl chain is present on the C-3 position, a β-cleavage with respect to the indole ring occurs; this is especially prominent for the indole-3-acetic acid anion for which the β-cleavage coincides with a decarboxylation, yielding the only fragment ion that is present in the CID spectrum (Revelou et al., 2019). The presence of a hydroxyl function on the benzene moiety of the indole ring will not affect this fragmentation mechanism. Indeed, in this study, the CID spectrum of the standard 5-hydroxyindole-3-acetic acid revealed only one fragment ion as a result of a decarboxylation. The aglycone structure of 5-hydroxyindole-3-acetic acid hexoside (anion at m/z 352.10229) was confirmed via MS^3 fragmentation of the aglycone-representing MS^2 ion (m/z 190). The MS^2 ion at m/z 308 resulted from a decarboxylation, indicating a free acidic function on the glycoside. The absence of MS^2 ions as a result of hexose cross-ring cleavages (Carroll et al., 1995; Mulroney et al., 1999) disproved the presence of a hexose ester. Therefore, the hexose is attached to the phenolic function. In the case of the 2-hydroxyindole-3-acetyl hexose structure, the hydroxyl function is vicinal to the alkyl chain, which might affect the predominance of the β-cleavage: the MS^3 spectrum of the MS^2 aglycone ion (m/z 190) was still dominated by a decarboxylation, yielding the ion at m/z 146, but was also indicative for the occurrence of a dehydration (MS^3 ion at m/z 172), and a combined decarboxylation and dehydration (MS^3 ion at m/z 128). The C-2 rather than the C-3 position is bearing the hydroxyl function, because the presence of a 3-hydroxyl group would have led to an acetic acid loss (Grossert et al., 2006). An acetic acid loss upon MS^3 fragmentation of the MS^2 aglycone ion enabled the characterization of zeanoside C because this indole bears a 2-hydroxyl function.

A dihydroxyindole-3-acetic acid (m/z 206.04519) was characterized of which the MS^2 spectrum was dominated by the ion at m/z 162 as a result of decarboxylation. A small peak was observed at m/z 188 resulting from dehydration. Both 5,6-dihydroxyindole-3-acetic acid and 2,7-dihydroxyindole-3-acetic acid are expected to expel water upon fragmentation because of the presence of an *ortho*-dihydroxyl and a 2-hydroxyl function, respectively. Because a combined decarboxylation and dehydration was absent, yet expected when a 2-hydroxyl function would have been present, this compound was putatively

characterized as 5,6-dihydroxyindole-3-acetic acid. A large number of dihydroxyindole-3-acetic acid hexosides were observed. In the MS^n spectra of many of them, characteristic product ions were present at m/z 350, 248, 206, 204, 188, 186, 176, 162, 160. As an example, the gas-phase fragmentation of the precursor ion from dihydroxyindole-3-acetic acid (phenylacetyl) hexoside that leads to this set of product ions is explained in Supplemental Figure 18.

Benzenoids. The CID spectra of benzenoid negative ions have been previously discussed (Morreel et al., 2014). Some dihydroxybenzoyl hexosides were also observed of which the CID spectra typically showed product ions resulting from an S_N2-like rearrangement between a phenoxide anion and the methoxy substituent of a neighboring benzene moiety. For example, in the case of fragmentation of the divanilloyl hexose ion, this rearrangement involved methyl transfer with the formation of the dihydroxybenzoyl dimethoxybenzoyl hexose ion. Fragmentation of the latter yielded the m/z 153 and 297 product ions that represented a dihydroxybenzoate and a dihydroxybenzoyl anhydrohexose ion.
Other CID spectra were less straightforward to elucidate, e.g., the CID spectrum of benzoyl glycerol hexuronate. This CID spectrum is dominated by the m/z 249 peak resulting from benzoic acid loss, the ion of the latter being present at m/z 121. The product ion at m/z 249 splitted further into the hexuronate ion at m/z 193 that subsequently yielded the ion at m/z 175 upon dehydration.

Phenylpropanoids. The CID spectra of phenylpropanoid negative ions have been previously discussed (Morreel et al., 2014) and are well-known for the hydroxycinnamoyl quinic acids (Clifford et al., 2003). Typical for maize are the large number of *p*-coumaroyl esters (Supplementary data - Supplemental Data Set 1). These derivatives readily cleave upon CID, likely via an acylglycerol-like charge-remote ester cleavage (Stroobant et al., 1995), yielding the *p*-coumarate ion (m/z 163) which further dissociate by decarboxylation (m/z 119; Bandu et al., 2006) as shown in the CID spectrum of, for example, *p*-coumaroyl ethanetriol (Supplemental Figure 19). Being a gem diol, *p*-coumaroyl ethanetriol likely result from the hydration of *p*-coumaroyl glycolaldehyde following the electrospray ionization process. In addition, a hydroxycinnamoyl ester ion often generates an ynolate ion (at m/z 145 in the case of the CID spectrum of a *p*-coumaroyl ester) upon CID (Debrauwer et al., 1992; Fournier et al., 1993; 1995), e.g., in the CID spectra of *p*-coumaroyl glycerol. Glycerol esters containing two hydroxycinnamoyl moieties were also detected, i.e., *p*-coumaroyl feruloyl glycerol and diferuloyl glycerol. The main gas-phase fragmentations of the diferuloyl glycerol anion are shown in Supplemental Figure 15. The methyl transfer from one feruloyl moiety to the phenolic function of the other feruloyl moiety with formation of a dimethoxycinnamoyl moiety was confirmed by matching the MS^3 spectrum of the m/z 207 product ion with the MS^2 spectrum of a dimethoxycinnamic acid standard.
However, when *p*-coumarate is esterified to an alcohol function from an organic acid, the product ions representing the *p*-coumaric acid moiety can be low abundant, e.g., see the gas-phase fragmentations of the ions from the *p*-coumarate esters of shikimic acid, 2-hydroxyglutaric acid and 2-hydroxyadipic acid among others. To exemplify the structural elucidation process, the fragmentations of the latter ions are further detailed below.
p-Coumaroyl shikimate was characterized because its CID spectrum contained, in addition to product ions representing the *p*-coumaric acid moiety, product ions at m/z 155, 137, 111 and 93 that were also present in the CID spectrum of the shikimate anion (based on MS/MS spectra available in the FooDB, primary ID FDB003991).
p-Coumaroyl 2-hydroxyglutarate has a CID spectrum dominated by the m/z 129 ion that represents the dehydrated dicarboxylic acid moiety. Decarboxylation from this ion yielded the product ion at m/z 85. Both 2-hydroxyglutarate and citramalate might represent the dicarboxylic acid moiety, yet the latter compound is hardly observed, whereas the former is widely present in the plant kingdom upon browsing the FooDB. Further proof was obtained by matching the 2-hydroxyglutarate-based MS^3 spectrum to the

MS^2 spectrum of a purchased 2-hydroxyglutarate standard (procedure similar to that displayed for *p*-coumaroyl 2-hydroxyadipic acid in Supplemental Figure 10).

The gas-phase fragmentation of *p*-coumaroyl 2-hydroxyadipic acid yielded either the *p*-coumarate ion with m/z 163 upon cleavage of the ester bond or the 2-hydroxyadipate ion with m/z 161 upon expelling the *p*-coumaric acid moiety as a ketene (Debrauwer et al., 1992; Fournier et al., 1993). The m/z 161 ion dissociated further by water loss, carbon dioxide loss, or a combined loss of water and carbon dioxide, yielding ions with m/z 143, 117 and 99, respectively. The latter reactions are typical for a dicarboxylic acid (Grossert et al., 2005). Further support was obtained by matching the 2-hydroxyadipate-based MS^3 spectrum to the MS^2 spectrum of a purchased 2-hydroxyadipate standard (see also Supplemental Figure 10).

Sinapyl(7-*O*-9)*p*-coumarate ester might be formed by a 1,4-nucleophilic addition of *p*-coumaric acid onto the 7-position of sinapaldehyde. Cleavages characteristic for an ester (see above) yielded the ions at m/z 163 (*p*-coumarate ion), m/z 145 (ynolate ion), m/z 207 (sinapaldehyde ion) and m/z 225 (7-hydroxysinapaldehyde ion). MS^3 fragmentations from the m/z 163 and m/z 207 product ions were identical to those of the ions of *p*-coumaric acid and sinapaldehyde.

Flavonoids. The gas-phase fragmentation channels of flavonoid aglycone anions are well-known (Hughes et al., 2001; Fabre et al., 2001; Cuyckens and Claeys, 2004; Morreel et al., 2006) as well as the effect of *O*-glycosylation on gas-phase fragmentation (Hvattum and Ekeberg, 2003; Ferreres et al., 2004; Yan et al., 2007). In addition, (bio)chemical knowledge and separation characteristics were used in structural elucidation (Cuyckens and Claeys, 2004). Biochemically, glucose and galactose are the main hexosylations occurring on flavonoids, whereas rhamnosylation, and xylosylation and arabinosylation are the principal deoxyhexosylation and pentosylation reactions, respectively. On a reverse-phase column, the galactosylated flavonoid will elute before the corresponding glucosylated flavonoid. The main disaccharides that are linked to flavonoids are rutinose [rhamnosyl(1→6)glucose] and neohesperidose [rhamnosyl(1→2)glucose], and the rutinosylated flavonoid elutes before the corresponding neohesperidosylated form. Glycosylation mainly occurs on the 7-position in the case of flavones and on the 3- and 7-positions in the case of flavanones and flavonols. Luteolin- and chrysoeriol-C-rhamnosyl(1→2)fucoside have been detected in maize (http://foodb.ca/).

Most tricin glycosyl-hexuronide ions showed a loss of 134 Da upon CID as a result of a cross-ring cleavage of the hexuronide moiety. In the case of, e.g., the tricin glycoside shown in Supplemental Figure 20, the resulting $^{0,2}X^-$ product ion (following the nomenclature of Domon and Costello, 1988) was observed at m/z 503. Together with the $^{1,5}X^-$ product ion formation, leading to the product ion at m/z 357, this indicates that tricin and a pentose moiety are linked to the 1- and 2-position of the hexuronic acid moiety, respectively. Because the flavone 5-*O*-position is involved in a hydrogen bond to the 4-*O* acyl group, and the 4'-*O*-position is sterically hindered in the case of tricin (Wojakowska et al., 2013), the hexuronic moiety is likely linked to the 7-*O*-position of tricin. Therefore, the CID spectrum represents the fragmentations of the tricin-7-*O*-pentosyl-(1→2)-hexuronide anion (Supplemental Figure 20).

Dependent on whether flavonoids are *O*- or *C*-glycosides, glycosidic bond cleavages (e.g., -162 Da loss in the case of a hexose) or cross-ring cleavages (-60, -90 and -120 Da losses) are favored (Ferreres et al., 2003; Wojakowska et al., 2013). The MS^n spectra of 6-*C*-hexosyl-8-*C*-pentosyl-apigenin is shown in Supplemental Figure 21. *C*-glycosylation occurs on the 6- and/or 8-position. Two rules aid annotating the linkage position on the aglycone in the case of two different glycosyl groups (Ferreres et al., 2003): (i) fragmentation occurs mainly from the 6-*C*-glycosylated position, and (ii) losses of -120 and -60 Da occur predominantly from hexosyl and pentosyl groups, respectively. The *C*-glycosylated flavonoids maysin and apimaysin, contain a rare sugar residue and the dissociation pathways of the maysin anion upon CID are shown in Supplemental Figure 22.

Oligolignols/(neo)lignans. Oligolignols are formed as intermediates during lignin polymerization, yet some of them are also synthesized as specialized, formerly called secondary, metabolites. Indeed, intracellular coupling between the oligolignol monomers in poplar xylem and Arabidopsis leaves and vacuolar storage as glycosides in Arabidopsis leaves have recently been observed (Niculaes et al., 2014; Dima et al., 2015). The CID spectra of anions from "traditional" oligolignols derived from 8–*O*–4-, 8–8- and 8–5-coupling of the monolignols *p*-coumaryl alcohol (H unit), coniferyl alcohol (G), and sinapyl alcohol (S) have been unraveled by Morreel et al. (2004a; 2004b; 2010a; 2010b) and Eklund et al. (2008). In addition, the fragmentations of oligolignol anions bearing "non-traditional" units derived from the incorporation of, e.g., coniferaldehyde (G'), sinapaldehyde (S'), and vanillin (V'), have been described in Morreel et al. (2004a; 2004b; 2010a; 2010b), Dauwe et al. (2007), Vanholme et al. (2010), and Lan et al. (2016). Morreel et al. (2010a; 2010b) has described two types of product ions in the CID spectra of oligolignols: type I fragmentations are small neutral losses that hint at the linkage type between the oligolignol units, whereas type II fragmentations cleave the linkage itself, hence, yielding information on the moieties that were connected by the linkage.

In maize, some oligolignols were detected that have not been previously found in LC-MS profiles. Guaiacylglycerol-(8–*O*–4)-syringylglycerol-(8–*O*–4)-*p*-coumaric acid, simply named G(8–*O*–4)S(8–*O*–4)*p*CA, was characterized as a result of the presence of β-aryl ether, i.e., 8–*O*–4-, linkage-associated type I product ions at m/z 537 and 507, and type II product ions representing the guaiacylglycerol and S(8–*O*–4)*p*CA moieties at m/z 195 and 389. The MS^3 spectrum of the latter ion was indicative for a second 8–*O*–4-linkage (type I ions at m/z 371 and 341; type II ions at m/z 225, and 163 representing the syringylglycerol and p-coumaric acid moieties; m/z 119 arises from decarboxylation of m/z 163).

G(8–*O*–4)Sox(8–*O*–4)S showed neutral losses of 18, 30 and 48 Da indicative for the presence of a 8–*O*–4-linkage. Cleavage of the 8–*O*–4-linkage in the G(8–*O*–4)Sox moiety yielded the type II product ions at m/z 433 and 195, whereas cleavage of the 8–*O*–4-linkage in the Sox(8–*O*–4)S moiety formed the base peak at m/z 419, representing the G(8–*O*–4)Sox anion. Indeed, cleavage of the same linkage type in the Sox(8–*O*–4)S heterodimer by Tsuji et al. (2015) has also been shown to result in a base peak representing the 8-linked unit of the β-aryl ether, i.e., the Sox moiety.

Oxylipins. The low energy CID pathways from negative ions of saturated fatty acids obtained via ESI have initially been described by Kerwin et al. (1996), while other studies focused on hydroxylated saturated and unsaturated fatty acids (Kerwin and Torvik, 1996; Wheelan et al., 1993; 1996; 1996b; Moe et al., 2004). These studies have shown that losses of H_2O and CO_2 either alone or in combination readily happen. Furthermore, double bond rearrangements might occur substantially upon CID of unsaturated fatty acid ions; they do not bias determining the positions of hydroxyl groups via CID spectral elucidation. Hydroxyl substituents initiate α-eliminations through bonds that are allylic to a double bond via either a charge-driven or charge-remote mechanism, called type 3 or type 1 fragmentation by Wheelan et al. (1993). In the case the cleaved bond is apparently vinylic to a double bond, the α-elimination occurs following a double bond rearrangement. Again, a charge-driven or charge-remote mechanism can be considered that were referred to as type 4 or type 2 fragmentations. A type 5 fragmentation, representing an oxy-Cope rearrangement followed by a homolytic cleavage, occurs whenever a 3-hydroxy-1,5-hexadiene structure is present in the hydroxyfatty acid. CID of the negative ion of 13-hydroxy-9,11,15-octadecatrienoic acid resulted in type 3 and type 2 fragmentations of the C_{13}–C_{14} and C_{12}–C_{13} bonds, yielding the product ions at m/z 223 and 195. Further decarboxylation from the m/z 223 ion explained the ion at m/z 179. A type 5 fragmentation was responsible for the ion at m/z 224. In the case of 10,12-dihydroxy-8-dodecenoic acid (Supplemental Figure 23), product ions at m/z 155 and 183 were formed by α-eliminations: the former resulting from a type 2 fragmentation, the latter likely via a charge-remote fragmentation that involved a 6-membered cyclic transition state, yielding a water loss, an ethylene loss and an aldehyde-bearing carboxylate ion. Concerning 9,10-dihydroxystearic acid, α-hydroxy fragmentations of the corresponding

anion explained the ions at m/z 141, 171 and 201. The m/z 155 ion likely originated from the precursor ion in two steps: an ynolate ion was formed by water loss from the carboxylate group, which was then shortened to an aldehyde-containing ynolate ion via a C_9–C_{10} bond cleavage (Moe et al., 2004).

Organic acids. Bandu et al. (2004; 2006) and Grossert et al. (2005) studied the low energy CID fragmentations of ESI-generated negative ions from carboxylic acids. Further insight was gained by Kanawati et al. (2008b) and Kanawati and Schmitt-Kopplin (2010) via density functional theory (DFT) studies. As in the case of oxylipins, H_2O and CO_2 losses either alone or in combination readily happen. In the case of aconitic acid, these losses yielded the product ions at m/z 129 (CO_2 loss) and 111 (H_2O+CO_2 loss). Because of the proximity of both vinylic carboxylic acid functions in the *cis* form, a dehydration can readily occur with the charge remaining on the allylic carboxylic acid function. The resulting m/z 155 product ion was very small as it readily decarboxylated to the m/z 111 allylic carbanion. MS^3 fragmentation of the m/z 129 ion did not result in H_2O loss, supporting a pathway towards the m/z 111 ion in which dehydration occurs first. In the case of *trans*-aconitic acid, where both vinylic carboxylic acid functions are rather distant, a combined H_2O/CO_2 loss to the m/z 111 ion is less feasible. A methyl ester of *trans*-aconitic acid, i.e., (2Z)-2-(2-methoxy-2-oxoethylidene)butanedioic acid, was also observed. Decarboxylation from the allylic acid function yielded the ion at m/z 143. Charge delocalization from the resulting allylic carbanion expelled a methoxyl anion that remained in an ion-neutral complex. Proton transfer yielded a methanol loss and the ion at m/z 111.

Apocarotenoids. Insight into the gas-phase fragmentation of ESI-generated negative ions from apocarotenoids is mainly based on CID spectral studies of abscissic acid (m/z 263) and of a deuterated analogue (Chiwocha et al., 2003; Zhao et al., 2013). The two most abundant product ions were the result of decarboxylation (m/z 219) and loss of the hexanoate side chain, yielding a cyclohexenedione ion (m/z 153). However, the latter ion can be absent in the CID spectra of some apocarotenoids such as dihydrophaseic acid [(2*Z*,4*E*)-5-[(1*R*,3*S*,5*R*,8*S*)-3,8-dihydroxy-1,5-dimethyl-6-oxabicyclo[3.2.1]octan-8-yl]-3-methylpenta-2,4-dienoic acid]; here, the oxabicyclo[3.2.1]octanediol fused ring system yields a product ion at m/z 171 (Chiwocha et al., 2003). Spectral data for dihydrophaseic acid was recorded in this study (Supplemental Data Set 1). Further authentication was based on the comparison of its MS^2 spectrum (MS^2: m/z 237, 219, 171, 123, 207, 201, 189, 163, 263) to that of a purchased stereomer [(2*Z*,4*E*)-5-[(1*S*,3*R*,5*S*,8*S*)-3,8-dihydroxy-1,5-dimethyl-6-oxabicyclo[3.2.1]octan-8-yl]-3-methylpenta-2,4-dienoic acid; Analyticon; MS^2: m/z 237, 219, 171, 123, 207, 201, 189, 153, 263]. Relative abundances of the product ions were the same in both CID spectra except for the low abundant product ions at m/z 163 and 153 in the spectra of dihydrophaseic acid and of its stereomer standard, respectively.

Compound class distribution across maize organs.

Most compound classes were more abundant in a particular organ except for the phenylpropanoids which were abundant in all organs (Table 2). Phenylpropanoids serve as precursors for a variety of other specialized aromatic pathways (Figure 3). Among the phenylpropanoids, many *p*-coumarate esters/amides, and to a lesser extent caffeate and ferulate esters/amides, were observed, bearing moieties derived from glycolaldehyde (in its hydrate form, ethanetriol), 2-hydroxyglutaric acid, 2-hydroxyadipic acid, isocitric acid, putrescine, hexaric acid, threonic/erythronic acid, shikimic acid, hexose, quinic acid, glycerol, tyramine, and hydroxycitric acid. Many of these acids and amines are chiral and are of interest for the chemical and pharmaceutical industries. For example, hydroxycitric acid has received a lot of attention owing to its anti-obesity effect (Rao and Sakariah, 1988) and, recently, also as a promising agent for the treatment of kidney stones (Kyada et al., 2017; Kelland et al., 2018). High concentrations of hydroxycitric acid have been found in a few tropical plant species, e.g., in *Hibiscus sabdariffa* (Da-Costa-

Rocha et al., 2014) and *Garcinia* species (Jena et al., 2002), and, mainly because of the fat-burning property of hydroxycitric acid, extracts from the latter serve as dietary supplements or as food ingredients. Because of these applications, a combined DNA barcoding and NMR approach has recently been established to authenticate *Garcinia* food supplements (Seethapathy et al., 2018). The concatenation of hydroxycinnamic acids to such a variety of acids and amines allowed their efficient separation via reverse-phase LC. As the acids are readily hydrolyzed from their corresponding esters, this offers a potential future platform for the preparative isolation of any acid or amine of interest. Such a platform would benefit from the genome sequence (Schnable et al., 2009) and SNP data that are available for maize (Xu et al., 2017), allowing a potential production increase via, e.g., marker-assisted breeding or pathway engineering.

Our constructed CSPP networks show that stems are rich in oligolignols, flavonolignans, and (neo)lignans (Table 2; Figure 5). Oligolignols are intermediates during lignification of the cell walls of vessels and fibers. Lignification, the oxidative coupling of lignin monomer radicals in a combinatorial process, is essential to allow the primary mechanical support and water and nutrient transport functions of the stem. All characterized flavonolignans are coupling products between the classical lignin monomers (the monolignols) and tricin, a flavone that is abundantly present in maize and other commelinid monocots (Lan et al., 2016b). These tricin-based flavonolignans are similar to oligolignols in that they are small oxidative coupling products of lignin monomers. Indeed, tricin was recently shown to be a lignin monomer in grasses (del Rio et al., 2012; Lan et al., 2015; 2016). Being glycosylated forms of di- and trilignols, the (neo)lignans are likely formed when the lignin monomers are oxidatively coupled in the cytoplasm rather than being transported to the cell wall of vessels or fibers for lignification. Upon glycosylation, these (neo)lignans might be stored in the vacuoles of, e.g., parenchymous cells (Dima et al., 2015). Some of these (neo)lignans might serve to protect the cell against the oxidative stress that accompanies the lignification process (Niculaes et al., 2014).

As with stems, leaves are also rich in (neo)lignans and flavonolignans. Leaves are also enriched in *O*- and *C*-glycosylated flavonoids and benzoxazinoids (Table 2). *In planta*, flavonoids have diverse functions (Falcone Ferreyra et al., 2012). The biosynthesis pathways of *O*- and *C*-glycosylated flavonoids follow rather different paths. *O*-glycosylation occurs after formation of the flavonoid backbone structure via the so-called "backbone first" pathway (Jiang et al., 2016). In contrast, the inner ring (C-ring) of the flavanone has to be opened to allow *C*-glycosylation in the so-called "decoration first" pathway (Figure 3). The characterized flavonoid aglycones belonged to the flavanones, flavones, or flavonols; their glycosides were often connected to other specialized metabolic classes, mainly benzenoids, phenylpropanoids, and indolics.

In addition to their presence in leaves, benzoxazinoids are also enriched in the maize ear; after oligolignols, benzoxazinoids are the most prominent aromatic class of compounds in this organ (Table 2). Benzoxazinoids function primarily as phytoanticipins, constitutively produced plant defensive compounds; they are synthesized from indole (Figure 3) and accumulate in the vacuole as their (2*R*)-2-β-D-glucosides (Niculaes et al., 2018; Zhou et al., 2018). Deglucosylation only occurs upon cell disruption when the vacuole-localized (2*R*)-2-β-D-glucoside gets into contact with plastid- or cell-wall-localized glucosidases (Niculaes et al., 2018). The benzoxazinoid composition in the field-grown maize plants showed clear evidence for cell disruption during plant development due presumably to infection or herbivory (Wouters et al., 2016). The detection of both the (2*R*)- and (2*S*)-2-β-D-glucosides of the more prominent benzoxazinoids indicated partial hydrolysis of the (2*R*)-2-β-D-glucosidic forms to racemic aglycone mixtures that were subsequently re-glucosylated. Furthermore, the cell disruption-induced deglucosylation yields an electrophilic benzoxazinoid aglycone that readily reacts with many chemical functions: an intramolecular reaction explained the observation of the benzoxazolinone derivates, whereas other derivatives were clearly due to the reactions of benzoxazinoids with nucleophilic species. Presumably, many of the higher molecular weight (> 700 Da) metabolites represent reaction products of benzoxazinoids with nucleophilic species.

Among the profiled organs, late cobs are relatively poor in aromatic compounds, except for the indolics. The latter compound class, comprising various glycosides of hydroxyindole- and dihydroxyindole-3-acetic acid, is even much prevalent in the tassel. The tassel also contains oligolignols, *O*- and *C*-glycosylated flavonoids, flavonolignans, benzenoids, and phenylethanoids. Among the phenylethanoids were hydroxyphenylacetic (HPAA) and phenylacetic acid (PAA), which are present in a wide variety of glycosides (Table 2).

SUPPLEMENTAL TABLES

Supplemental Table 1. Number of DynLib subDB entries for each organ and genotype.

Genotype	Organ	QTOF_neg	QTOF_pos	FTMS_neg	FTMS_pos
CML91	Ear	1094	894	515	252
	Late cob	1133	940	606	326
	Leaf	1474	1107	616	370
	Stem	901	832	595	269
	Tassel	1379	1022	548	473
H99	Ear	746	735	488	198
	Late cob	830	788	603	198
	Leaf	1474	1128	570	339
	Stem	760	793	518	226
	Tassel	1439	1148	598	458
W153R	Ear	930	825	590	225
	Late cob	1192	900	646	317
	Leaf	1458	1060	622	360
	Stem	728	727	538	204
	Tassel	1550	1105	599	489
OH43	Ear	976	793	618	237
	Late cob	1137	978	649	275
	Leaf	1552	115	629	365
	Stem	418	808	600	271
	Tassel	1537	1147	637	479

For QTOF or FTMS subDBs, each entry is an MS/MS spectrum or an MS^n spectrum, respectively. An MS^n spectrum represents an MS^2 spectrum and, optionally, one or more MS^3 spectra. In general, more MS/MS spectra than MS^n spectra are available per organ and genotype. As many CID spectra are present in multiple organs or genotypes, the column totals for each subDB are much larger than the number of unique CID spectra. Numbers in the manuscript refer to the number of unique CID spectra.

SUPPLEMENTAL FIGURES

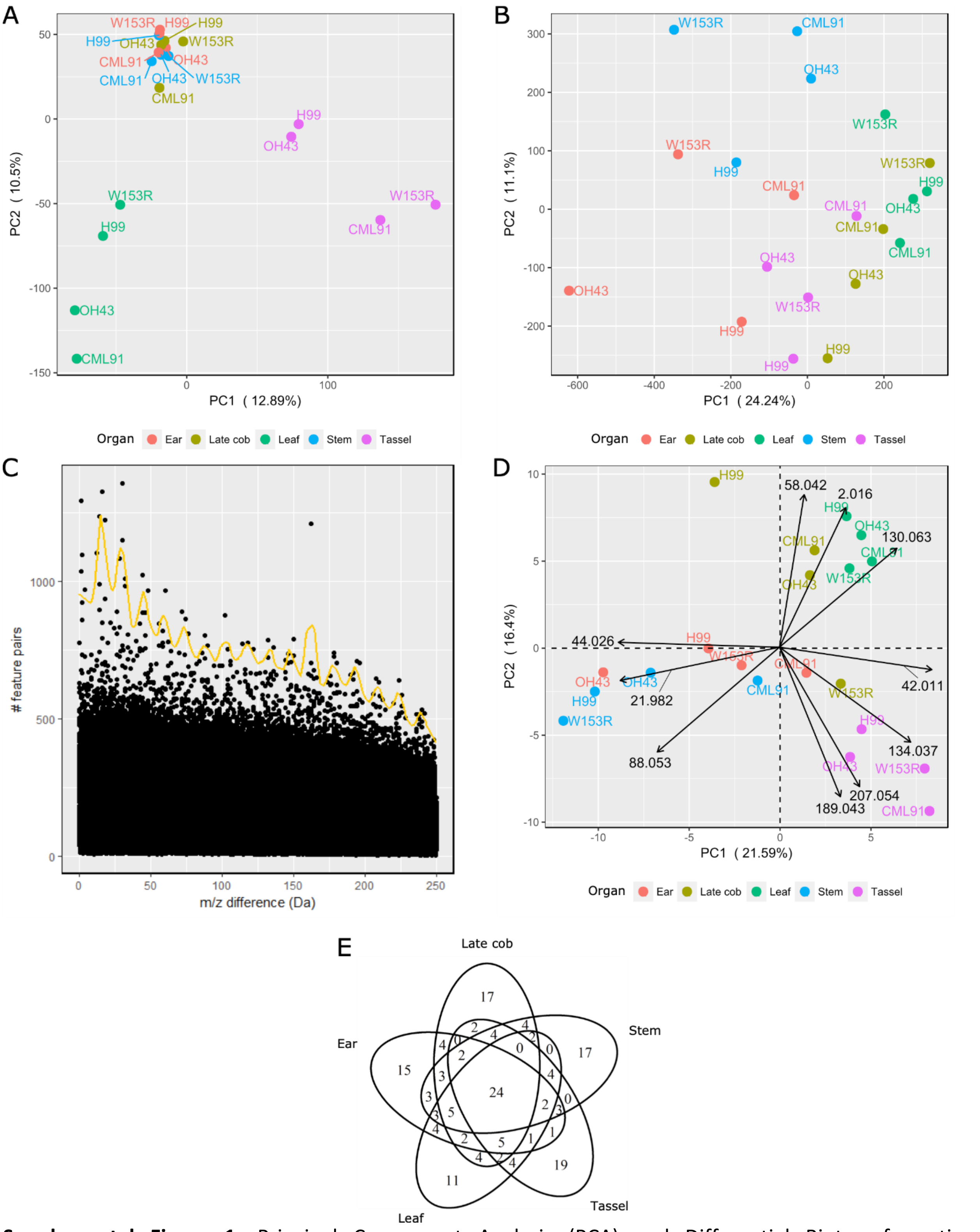

Supplemental Figure 1. Principal Component Analysis (PCA) and Differential Biotransformation Enrichment. All plots are based on positive ionization UHPLC-FT profiling. **A**. PCA plot based on the feature

abundances. Data were centered and unit variance-scaled. **B**. PCA plot of the number of feature pairs for all 250,000 mass differences (between 0 and 250 Da, with a precision of 0.001 Da). Mass differences were divided by their local frequency threshold following the approach in Morreel et al. (2014), and subsequently centered and unit variance-scaled. **C.** Manhattan plot showing the number of feature pairs for each mass difference between 0 and 250 Da (0.001 Da interval) based on the stem data from all genotypes. Filtering of frequently occurring mass differences is based on the threshold line shown in yellow (see Methods). **D**. Biplot (PCA plot + loading plot) based on the number of feature pairs for the selected mass differences, i.e., biotransformations. The loadings represent the top ten variables, i.e., mass differences, contributing to the principal components. **E**. Venn diagram of the tissue distribution of the biotransformations. Presence/absence of a biotransformation in a particular tissue is based on the threshold line displayed in the Manhattan plots (see C). PCA plots display the principal component 1 (PC1) and 2 (PC2) values. The variances explained by PC1 and PC2 are indicated between parentheses.

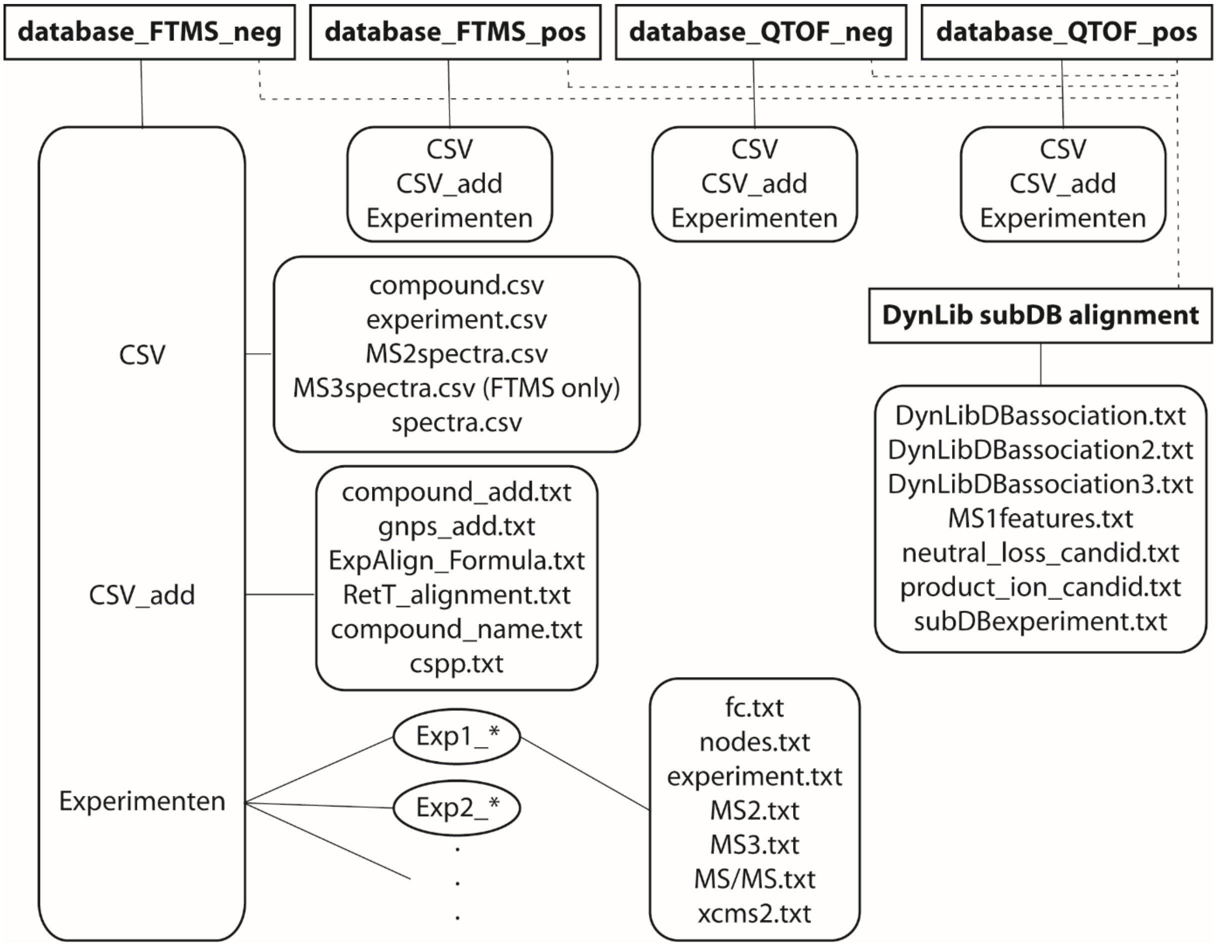

Supplemental Figure 2. Structure of the DynLib Database. The DynLib database comprises five directories, of which four represent the different subDBs (FTMS_neg, FTMS_pos, QTOF_neg and QTOF_pos). The fifth directory (DynLib subDB alignment) contains information to connect the four subDBs (as this directory is shared by the four subDBs, connections are indicated as dashed lines). Each subDB contains three directories (CSV, CSV_add and Experimenten). The CSV and CSV_add directories contain archived information of different LC-MS experiments. The Experimenten directory is subdivided in several directories that each contain the files of an individual LC-MS experiment (the processed data, CID spectra and other information) that serve as input for archiving into the DynLib database.

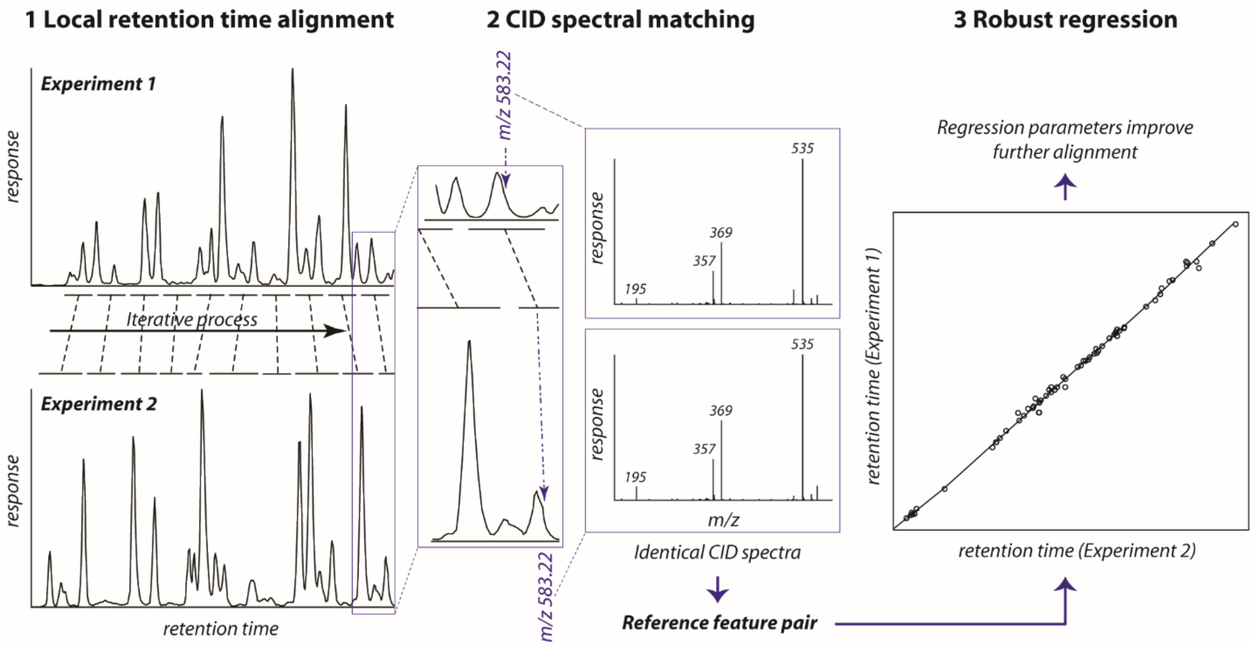

Supplemental Figure 3. Illustration of the Overall Alignment Procedure Between DynLib subDB Experiments (Referred to as Experiment 1 and 2). Iteratively throughout the chromatogram of Experiment 1, local regions of adjacent features are aligned to the corresponding regions in the chromatogram of Experiment 2. This yields a list of so-called reference feature pairs or RFPs. Next, this list is filtered by removing RFPs of which the features have dissimilar CID spectra. Subsequently, the retention times of Experiment 2 are regressed on those of Experiment 1 based on the filtered list of RFPs. This regression model enables then to compute corrected retention times for all features in Experiment 2. Finally, a new alignment is executed between features of both experiments. At this final step, alignment between a feature in Experiment 1 with another feature in Experiment 2 implies that: (i) the m/z values of both features are the same, (ii) the retention time of the feature in Experiment 1 is present within a pre-set window around the corrected retention time for the feature in Experiment 2, and (iii) the CID spectra of both features are similar.

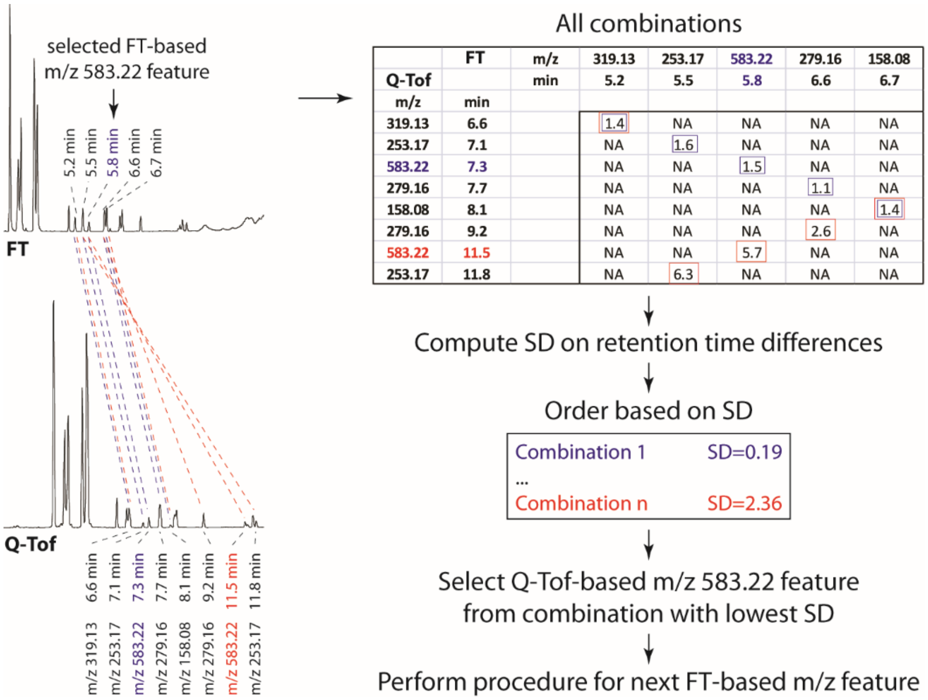

Supplemental Figure 4. Local Retention Time Alignment Procedure. See text (*Local retention time alignment*) for a description of the procedure.

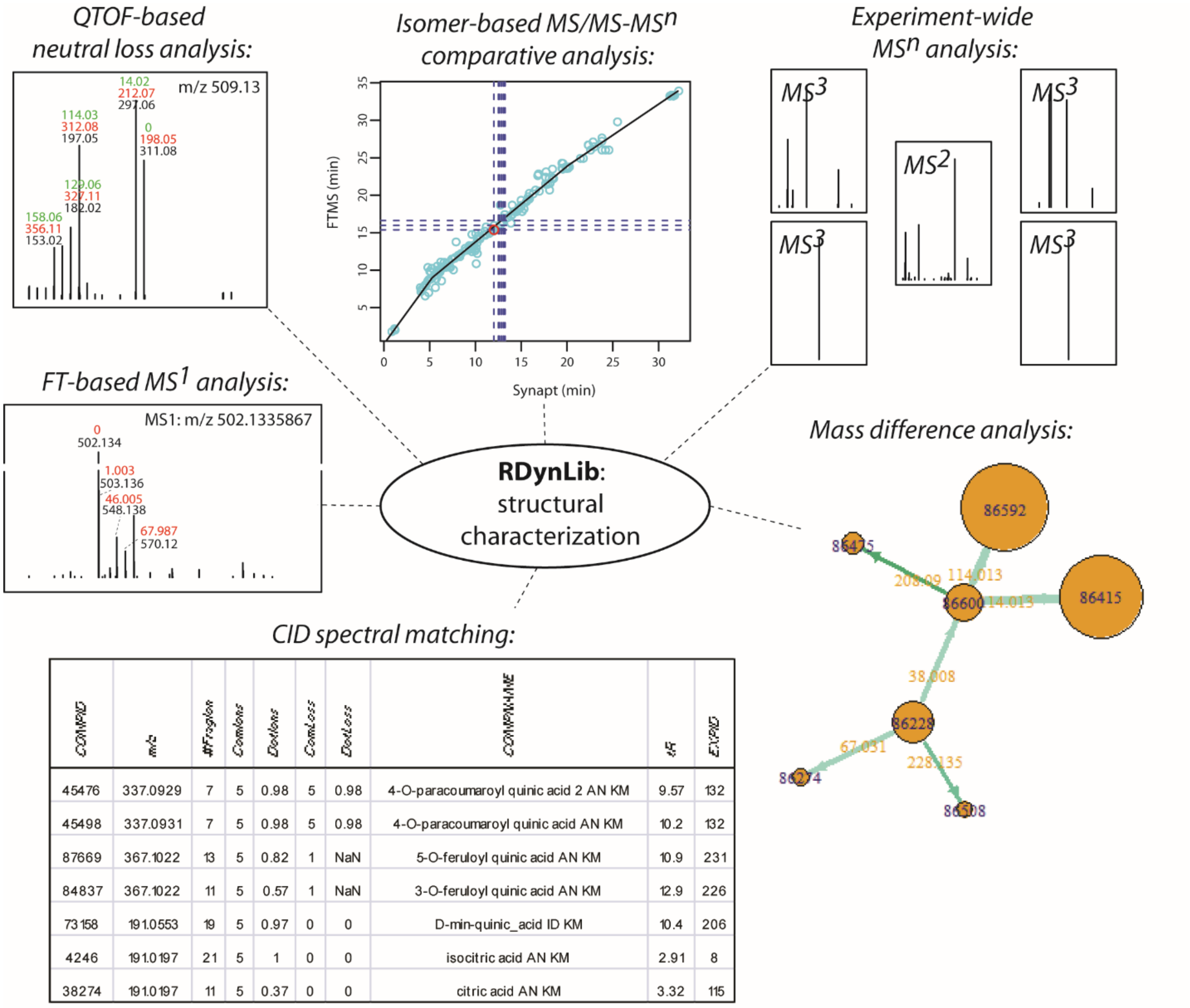

COMPID	m/z	#FragIon	ComIons	DotIons	ComLoss	DotLoss	COMPNAME	tR	EXPID
45476	337.0929	7	5	0.98	5	0.98	4-O-paracoumaroyl quinic acid 2 AN KM	9.57	132
45498	337.0931	7	5	0.98	5	0.98	4-O-paracoumaroyl quinic acid AN KM	10.2	132
87669	367.1022	13	5	0.82	1	NaN	5-O-feruloyl quinic acid AN KM	10.9	231
84837	367.1022	11	5	0.57	1	NaN	3-O-feruloyl quinic acid AN KM	12.9	226
73158	191.0553	19	5	0.97	0	0	D-min-quinic_acid ID KM	10.4	206
4246	191.0197	21	5	1	0	0	isocitric acid AN KM	2.91	8
38274	191.0197	11	5	0.37	0	0	citric acid AN KM	3.32	115

Supplemental Figure 5. Overview of RDynLib Hands-On Structural Characterization Approaches. See text (*Structural characterization tools in RDynLib*) for explanation.

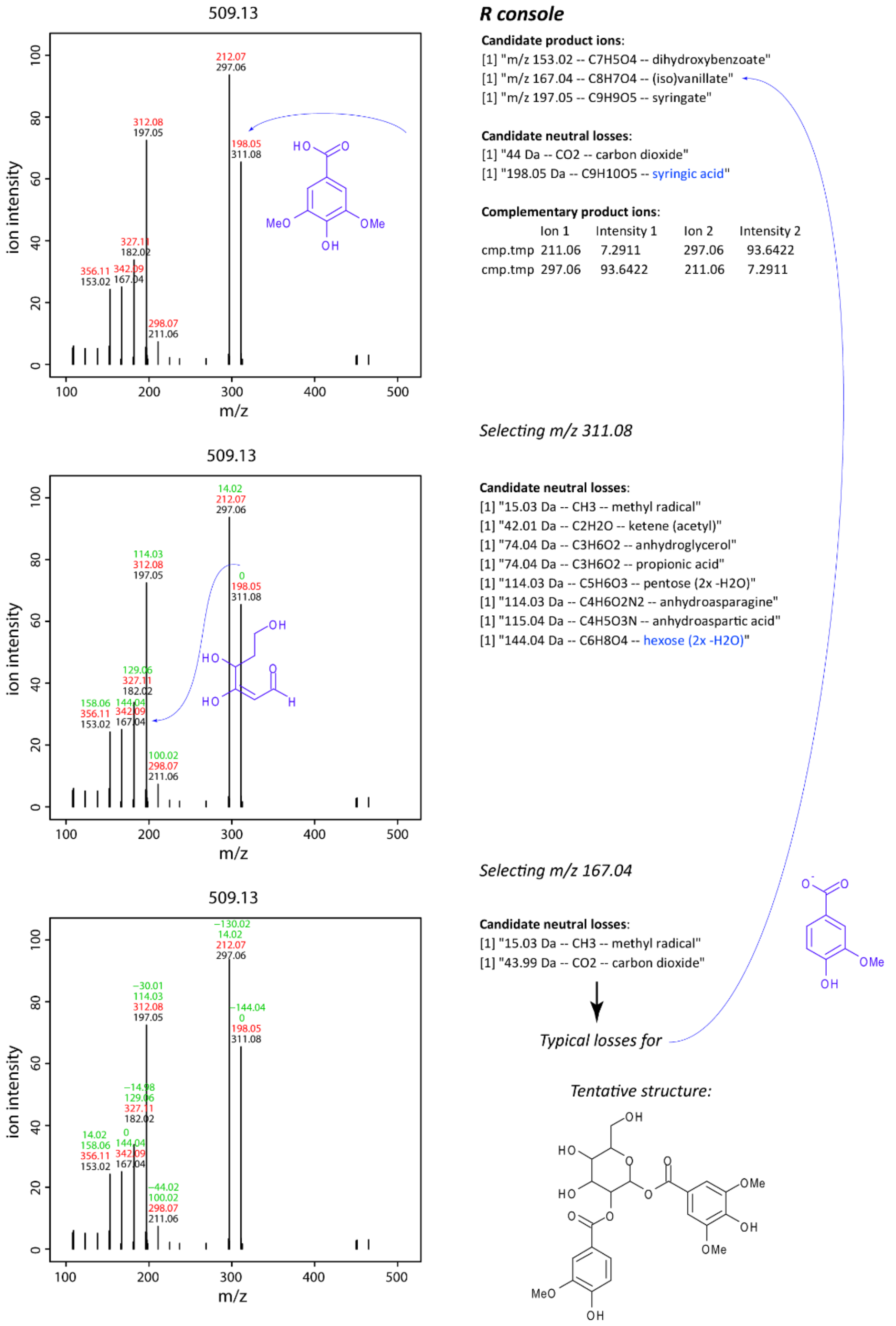

Supplemental Figure 6. QTOF-Based Neutral Loss Analysis. For each product ion (black label) in the MS/MS spectrum, the R console displays the m/z value, the candidate chemical formulae, and names of candidate structures. This information is also given for the candidate neutral losses (i.e., the mass differences between the precursor ion and the product ions; red label). Complementary product ions, i.e., ions of which the sum of the masses of their neutral molecules equals the mass of the precursor molecule, are also mentioned in the R console. In a first step, the neutral loss of 198.05 Da suggests a syringic acid moiety. From the corresponding product ion at m/z 311.08, the mass differences with the other product ions are requested (green label). A neutral loss of 144.04 Da, suggesting a dianhydrohexose moiety, yields the product ion at m/z 167.04 that was initially annotated as a vanillate ion. Further neutral losses from

this ion (green label) further support the latter moiety. Therefore, the precursor ion was characterized as vanilloyl syringoyl hexose.

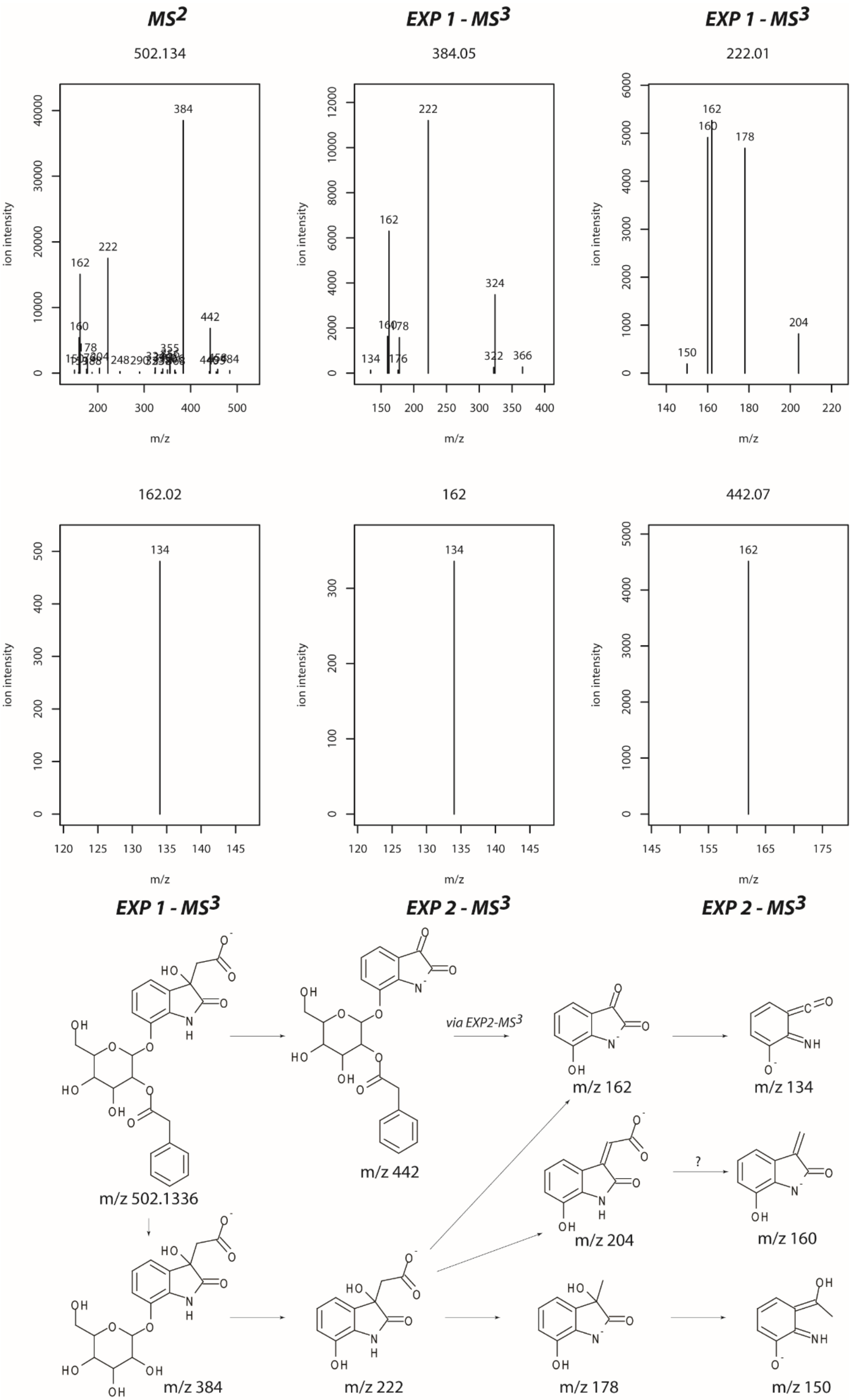
MS2
502.134
EXP 1 - MS3
384.05
EXP 1 - MS3
222.01
162.02
162
442.07
ion intensity
m/z
EXP 1 - MS3
EXP 2 - MS3
EXP 2 - MS3
via EXP2-MS3
m/z 502.1336
m/z 442
m/z 162
m/z 134
m/z 204
m/z 160
m/z 384
m/z 222
m/z 178
m/z 150

Supplemental Figure 7. Experiment-Wide MS^n Analysis. The structural characterization of 3,7-dihydroxy-2-indolinone-3-acetic acid-(phenylacetyl)-hexoside is displayed, for which additional support was obtained by analyzing multiple MS^3 spectra. Supplementary MS^3 spectra were recorded during a second LC-MS run, in which the MS^n data-dependent analysis settings were changed. Both chromatograms were entered into separate subDB experiments (indicated as EXP 1 and EXP 2) within the same DynLib subDB but are displayed together upon subDB experiment alignment. The displayed putative gas-phase fragmentations are mainly based on the MS^3 data from EXP 1; fragmentations based on MS^3 data of EXP 2 are explicitly mentioned. Excluding the 3-OH function as the site of hexosylation was only possible upon considering the MS^3 spectra of EXP 1 and 2 together. The m/z 178 was shown to fragment to the ion at m/z 150 (MS^3 spectrum not shown). Consequently, the m/z 160 ion is likely produced by carbondioxide loss from the m/z 204 ion rather than via dehydration of the m/z 178 ion. The m/z 442 ion in the MS^2 spectrum is due to acetate loss and was also observed as an in-source fragment in the MS^1 spectrum (see Supplemental Figure 9). CID spectra are entitled by the m/z value of the precursor ion.

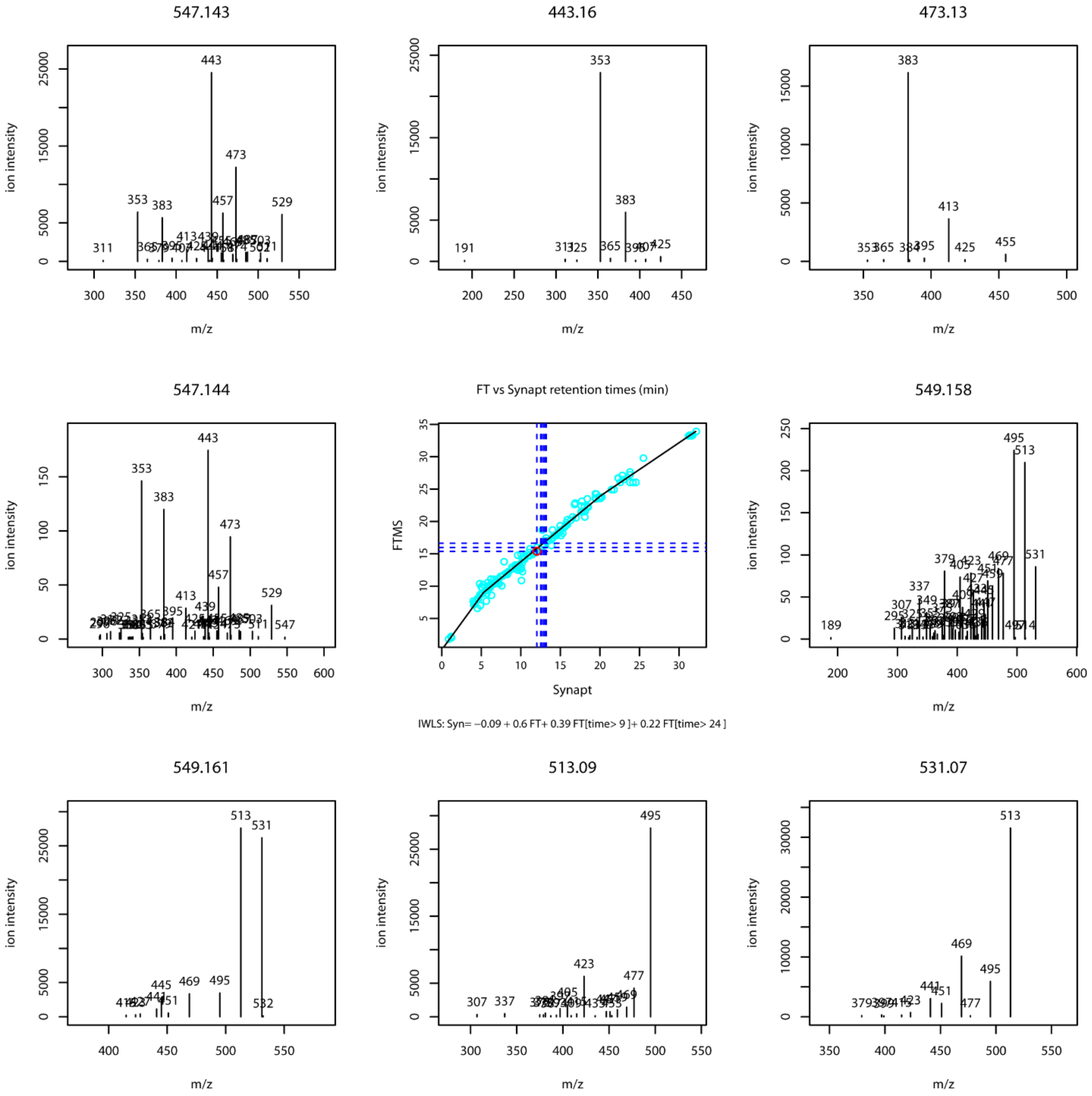

Supplemental Figure 8. Isomer-Based MS/MS-MS^n Comparative Analysis. All isomers of a selected ion and their CID spectra that are present in the QTOF- and/or FT-MS profiles are displayed via the *dynamCIDspecplot()* function. CID spectra are labeled by the m/z value of the precursor ion. Upper and lower rows represent the MS^2 and MS^3 spectra obtained in negative and positive ionization mode, respectively. Left and right plots of the middle row are the MS/MS spectra recorded in negative and positive ionization mode, respectively. The plot in the center of the figure represents the piecewise regression function between the retention times obtained on the FT and on the QTOF platforms. Circles are reference feature pairs (RFPs) derived from matching features among both platforms via the local retention time alignment procedure (Supplemental Figure 4). Dashed lines represent the retention times of the isomers. In R, it is possible to switch between the different isomers with the concomitant plotting of the corresponding CID spectra. The position on the piecewise regression plot is indicated by a red circle.

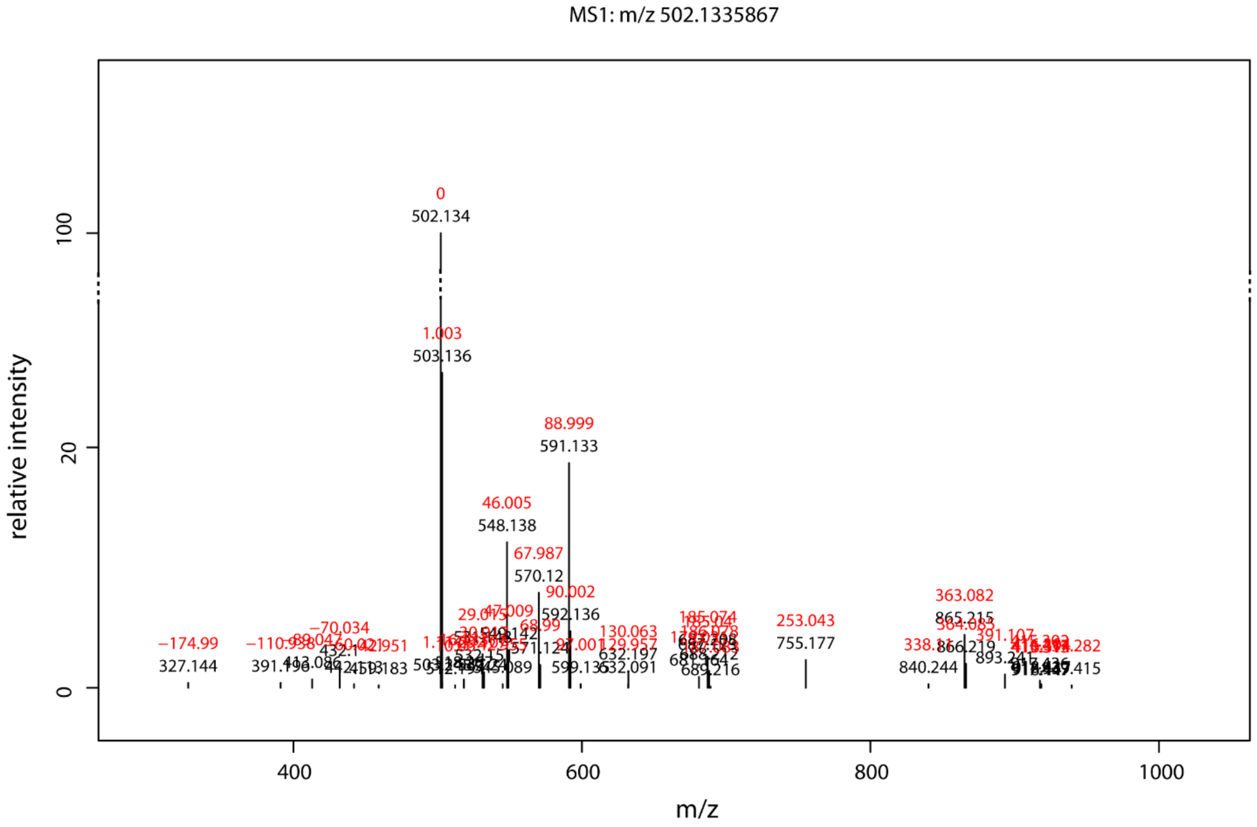

R console

	m/z	m/z diff	Rel.Int.	Type	COMPID	Combin	R2
1	502.1336	0	100	<NA>	74954	<NA>	1
…							
6	549.1422	47.0087	3.1	13C1 Formate	NA	<NA>	1
…							
21	503.1365	1.0029	26.2	13C1	74865	<NA>	1
22	548.1381	46.0045	12.1	Formate	NA	<NA>	1
23	442.1126	-60.021	0.3	Acetate	NA	<NA>	0.97
24	571.1241	68.9905	1.9	13C1 Sodium formate	NA	<NA>	0.96
…							
27	570.1203	67.9867	7.9	Sodium formate	NA	<NA>	0.97
…							
35	865.2151	363.0815	4.4	<NA>	NA	432.1 / 432.1	0.74

Supplemental Figure 9. FT-Based MS^1 Analysis. The m/z values of the ions and the corresponding mass differences versus the selected feature are labeled in black and red, respectively. The R console displays each feature (column m/z) with its corresponding mass difference with the selected feature (column m/z diff). It also displays the relative intensity versus the selected feature (the latter set at 100%; column Rel.Int.), the type (isotope, adduct, in-source fragment; column Type) of ion based on the mass difference, whether a CID spectrum is available for the ion (all CID spectra have a unique key called COMPID), and whether the ion is a combination of two other features in the MS^1 spectrum (one being present as a neutral in the complex; column Combin). The last column (column R2) represents the Pearson correlation coefficient computed between the abundance of the ion and that of the selected feature across multiple

chromatograms. In this example, the selected feature is the ion at m/z 502.1336. Isotopes (13C1) and buffer adducts (e.g., Formate) are indicated. The ion at m/z 442.1126 results from a loss of acetate owing to in-source rearrangement, indicating the presence of an acetyl function in the structure of the selected feature. Not all MS^1 features are shown. Rel.Int. Relative Intensity.

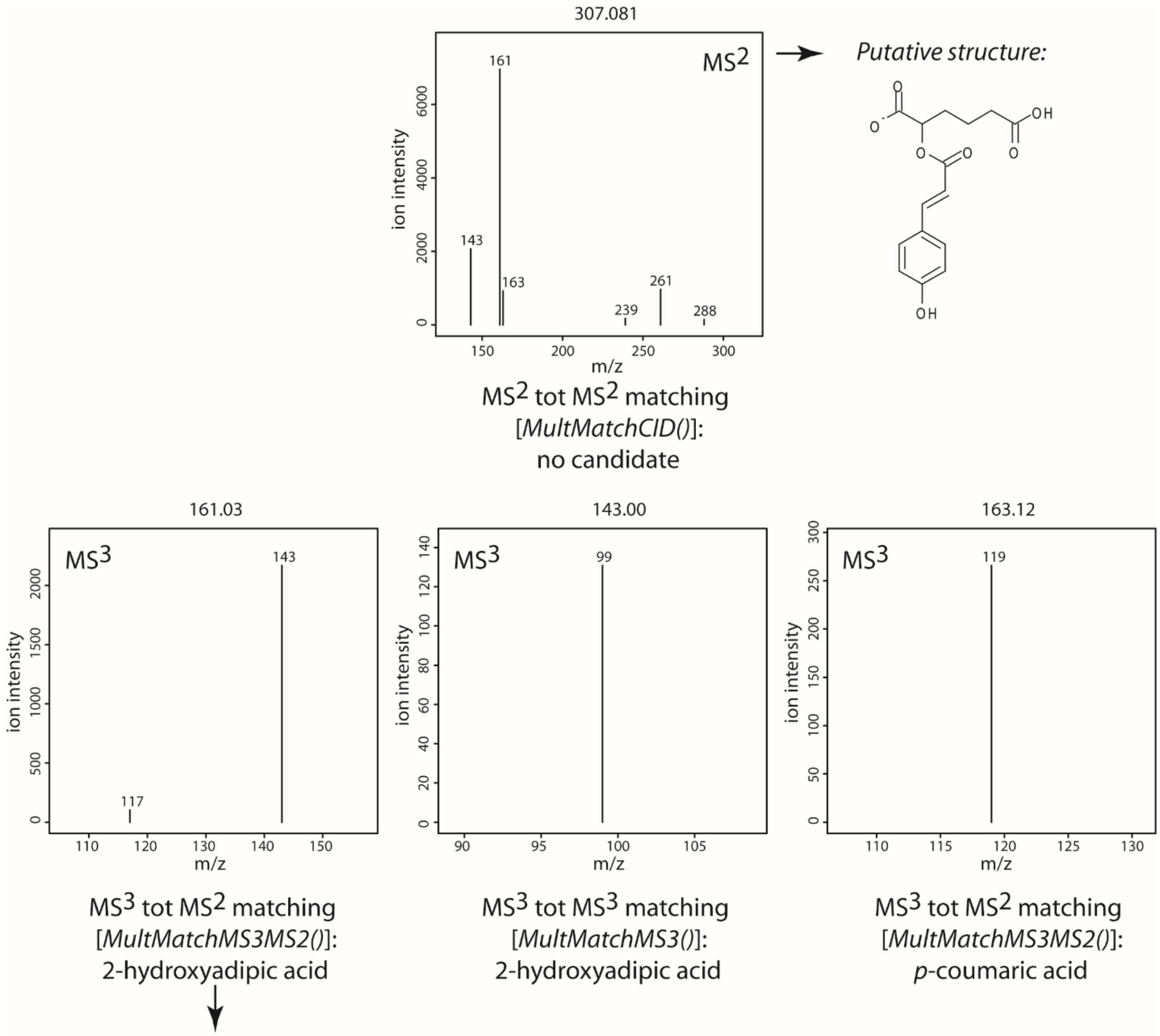

	COMPID	m/z	#FragIon	ComIons	DotIons	ComLoss	DotLoss	COMPNAME	tR	EXPID
28	32501	161.0456	3	2	1	2	1	2-hydroxyadipic acid ID KM	5.25	99
27	20789	161.0456	4	2	1	2	1	2-hydroxy-3-methylglutaric acid PU KM	3.19	59

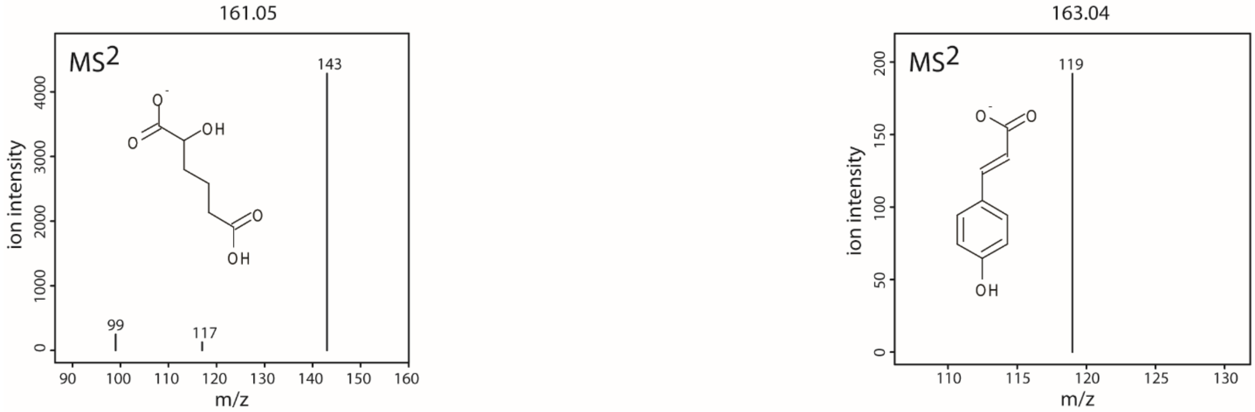

Supplemental Figure 10. CID Spectral Matching. MS^n spectra of an unknown feature and DynLib database MS^3 spectra of standard compounds are shown above and below the inserted table, respectively. CID spectra are entitled by the m/z value of the precursor ion. Matching of the unknown MS^2 spectrum to the DynLib database MS^2 spectra did not lead to any candidate structure. Matching of the unknown MS^3 spectra to the DynLib database MS^2 spectra showed that the MS^3 spectrum of the MS^2 product ion at m/z 163 matched with the MS^2 spectrum of *p*-coumaric acid. Furthermore, the base peak at m/z 161 in the unknown MS^2 spectrum could tentatively correspond with 2-hydroxyadipic acid or 3-hydroxy-3-methylglutaric acid, yet matching the MS^3 spectrum of m/z 161 to the DynLib database MS^2 spectra resulted in an identical match with the MS^2 spectrum of 2-hydroxyadipic acid. Thus, the unknown compound was characterized as *p*-coumaroyl 2-hydroxyadipic acid. The inserted table shows the output in the R console resulting from CID spectral matching. COMPID represents the unique key for a particular CID spectrum. Columns in the table are referring to this database spectrum: #FragIons, number of product ions in the database spectrum; ComIons, number of product ions in common between the database and the unknown spectra; DotIons, dot product computed for the common ions; ComLoss, number of neutral losses (mass differences between each product ion and the precursor ion) in common; DotLoss, dot product computed for the common neutral losses; COMPNAME, trivial name associated with the database spectrum; tR, retention time associated with the database spectrum; EXPID, subDB experiment to which the database spectrum belongs.

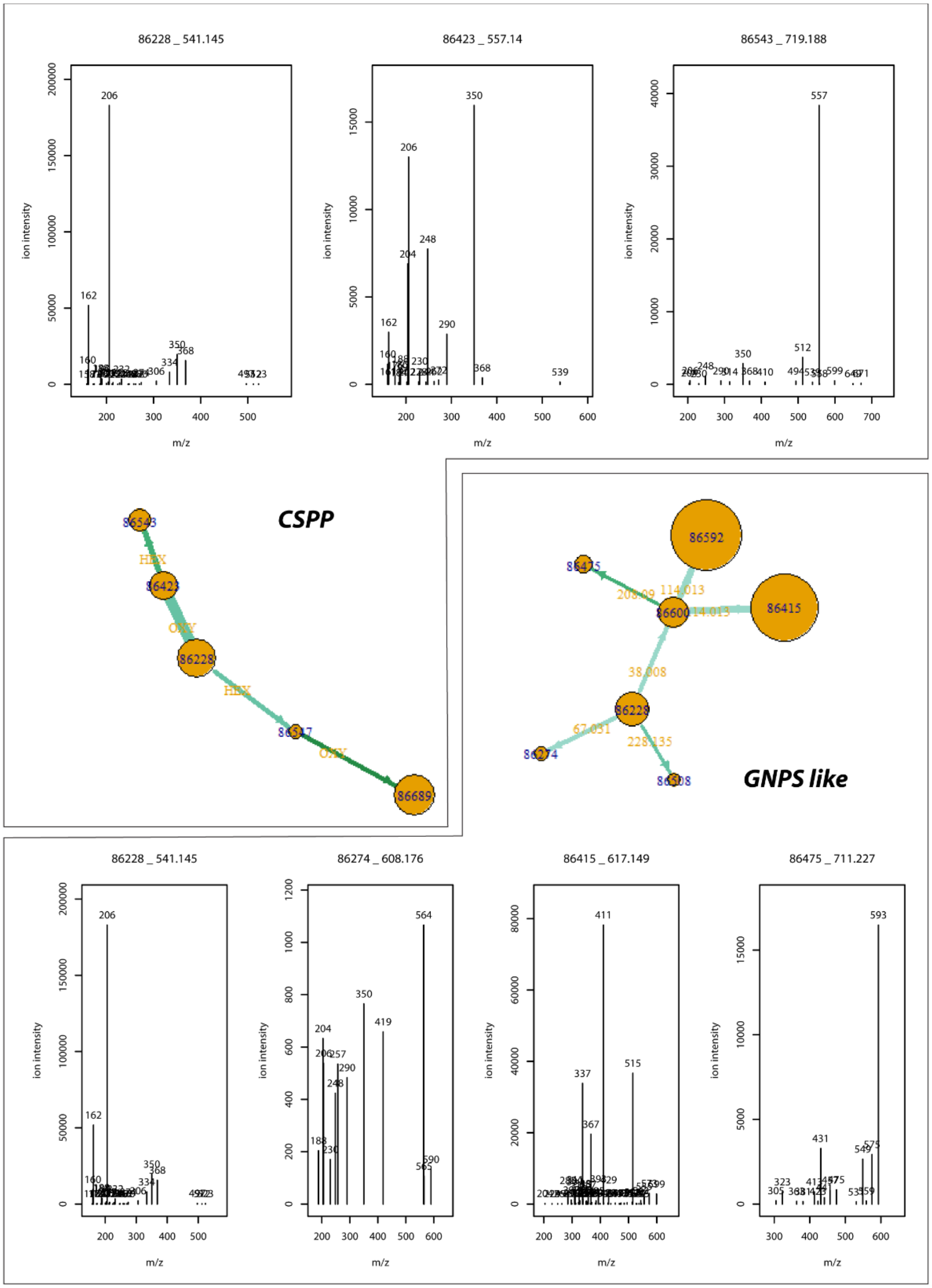

Supplemental Figure 11. Mass Difference Analysis. Networks (nodes and edges representing features and mass differences) comprising a restricted number of features neighboring a feature of interest are plotted based on the candidate substrate-product pairs (CSPP; Morreel et al., 2014) and on the Global Natural Product Social Molecular Networking (GNPS; Watrous et al., 2012) approaches. Each node is labeled with the COMPID, i.e., the unique identifier of the CID spectrum in the DynLib database. The edge is labeled with the mass difference in the case of the GNPS-like network and with the biotransformation (HEX, hexosylation; OXY, oxygenation) responsible for the mass difference in the case of the CSPP network. Node size represents the number of product ions in the CID spectrum. Edge width and edge color intensity

reflect the number of ions in common and the dot product, respectively. For a subset of nodes, the associated CID spectra are displayed. Each CID spectrum is entitled by a combination of the COMPID and the m/z value of the precursor ion.

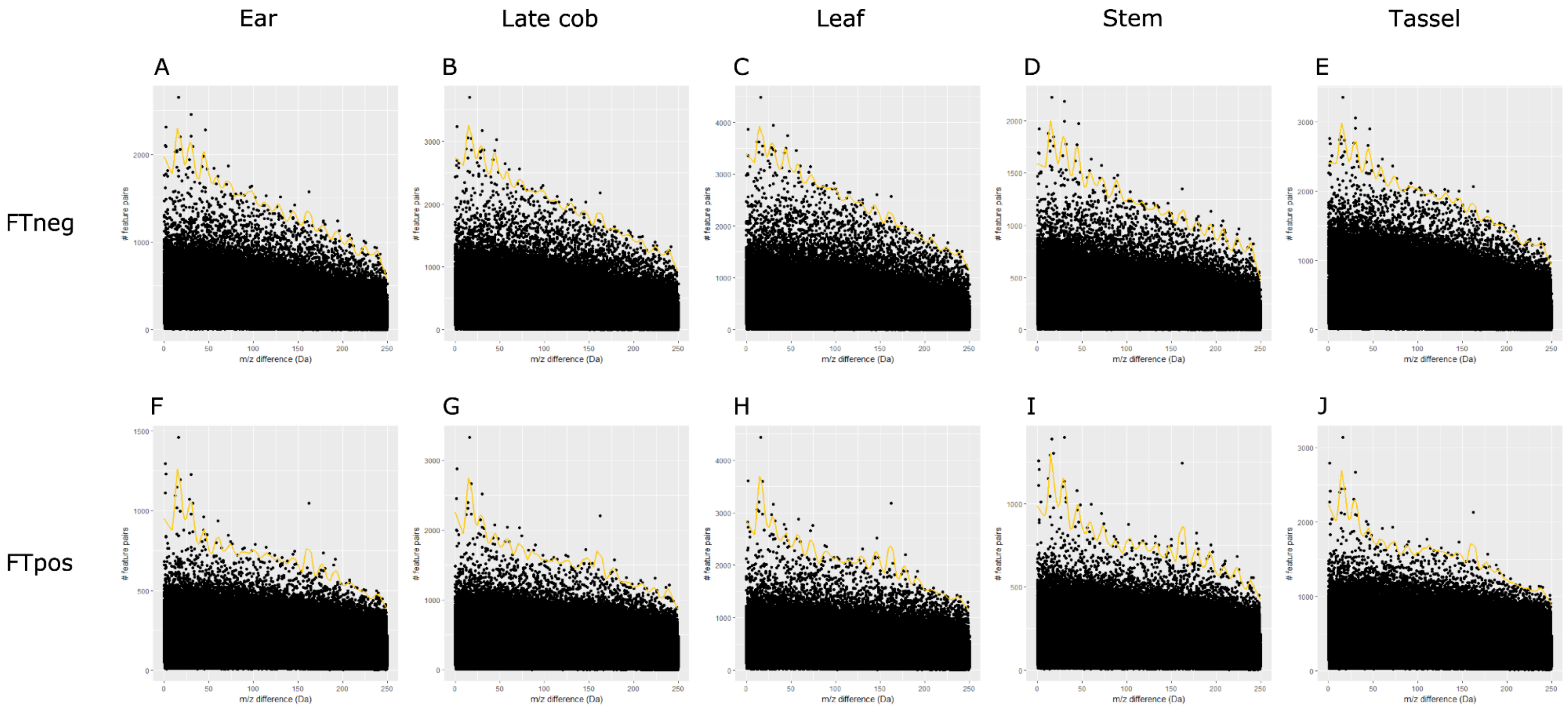

Supplemental Figure 12. Manhattan Plots Revealing Biotransformation Enrichment. Number of feature pairs are plotted for each mass difference between 0 and 250 Da (0.001 Da interval). Plots A-E and F-J are based on FTneg and FTpos data sets, respectively. Plots A and F, B and G, C and H, D and I, and E and J are based on profiles from stem, ear, late cob, leaf and tassel, respectively. Filtering of frequently occurring mass differences is based on the threshold line shown in yellow (Morreel et al., 2104).

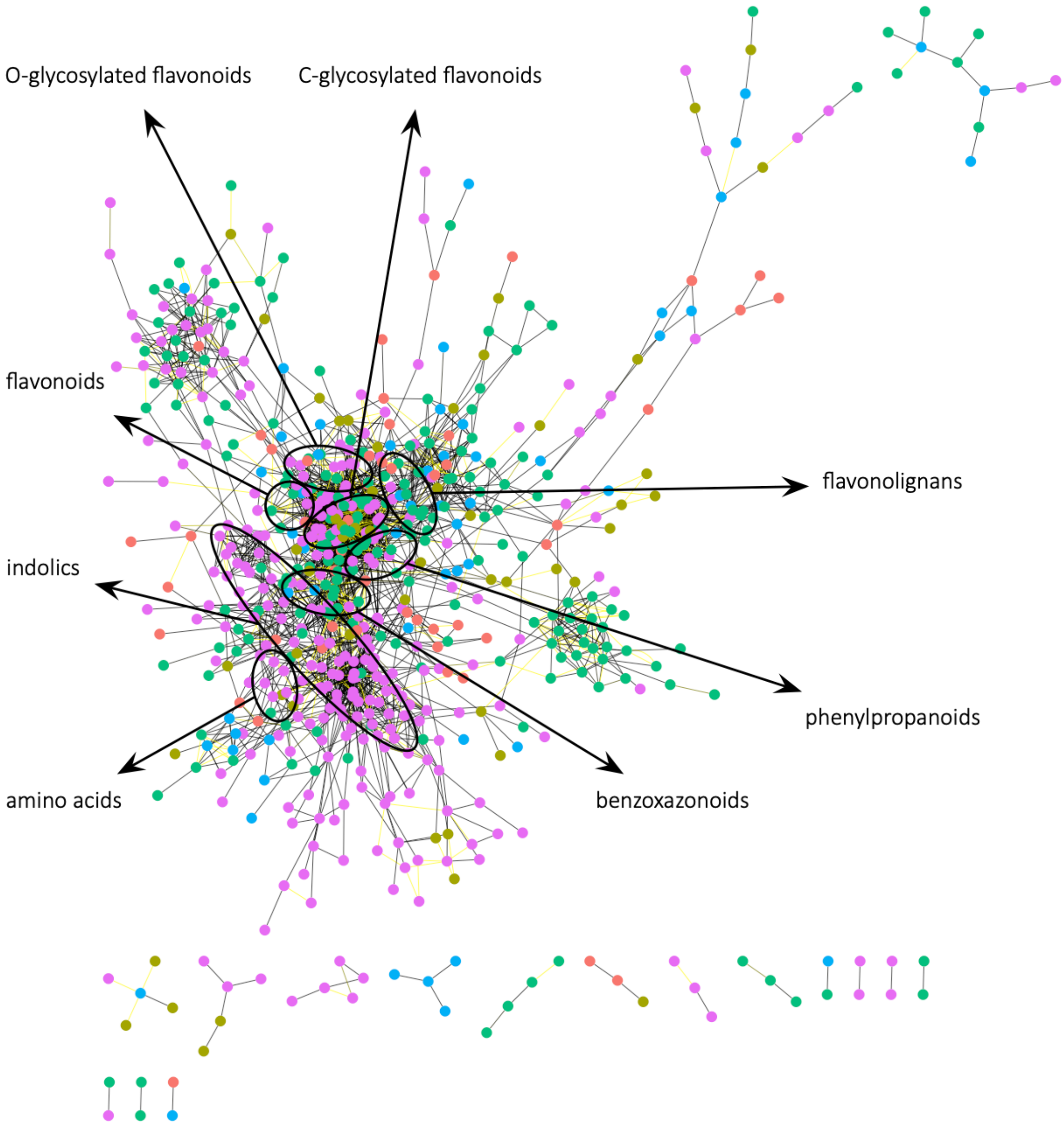

Supplemental Figure 13. CSPP Network Based on the FT Chromatograms Using Positive Ionization Mode. The node color, i.e., red, greenish brown, green, blue and purple, represents the tissue in which the feature was the most abundant (the ear, late cob, leaf, stem or tassel, respectively). The color of the network edges shows the MS^2 similarity of the substrate and the product.

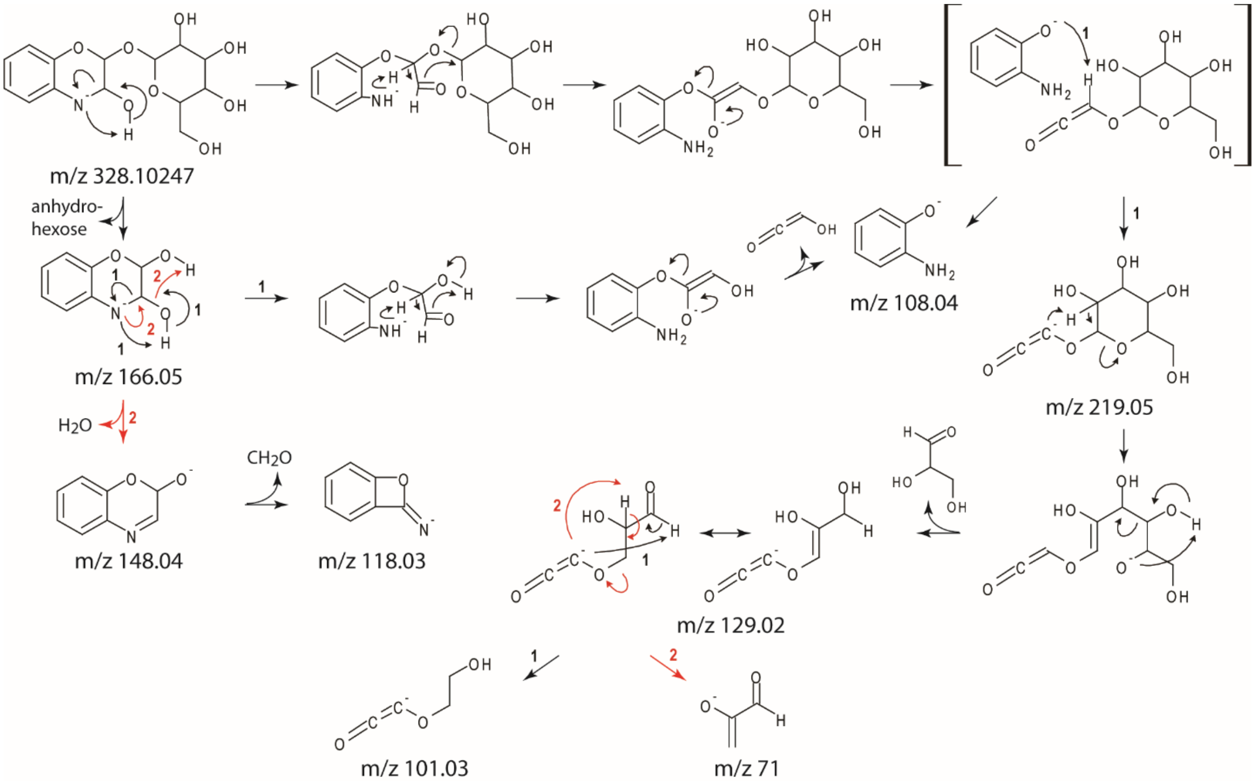

Supplemental Figure 14. Tentative Gas-Phase Fragmentation Pathway of Benzomorpholine-2,3-Diol Hexoside

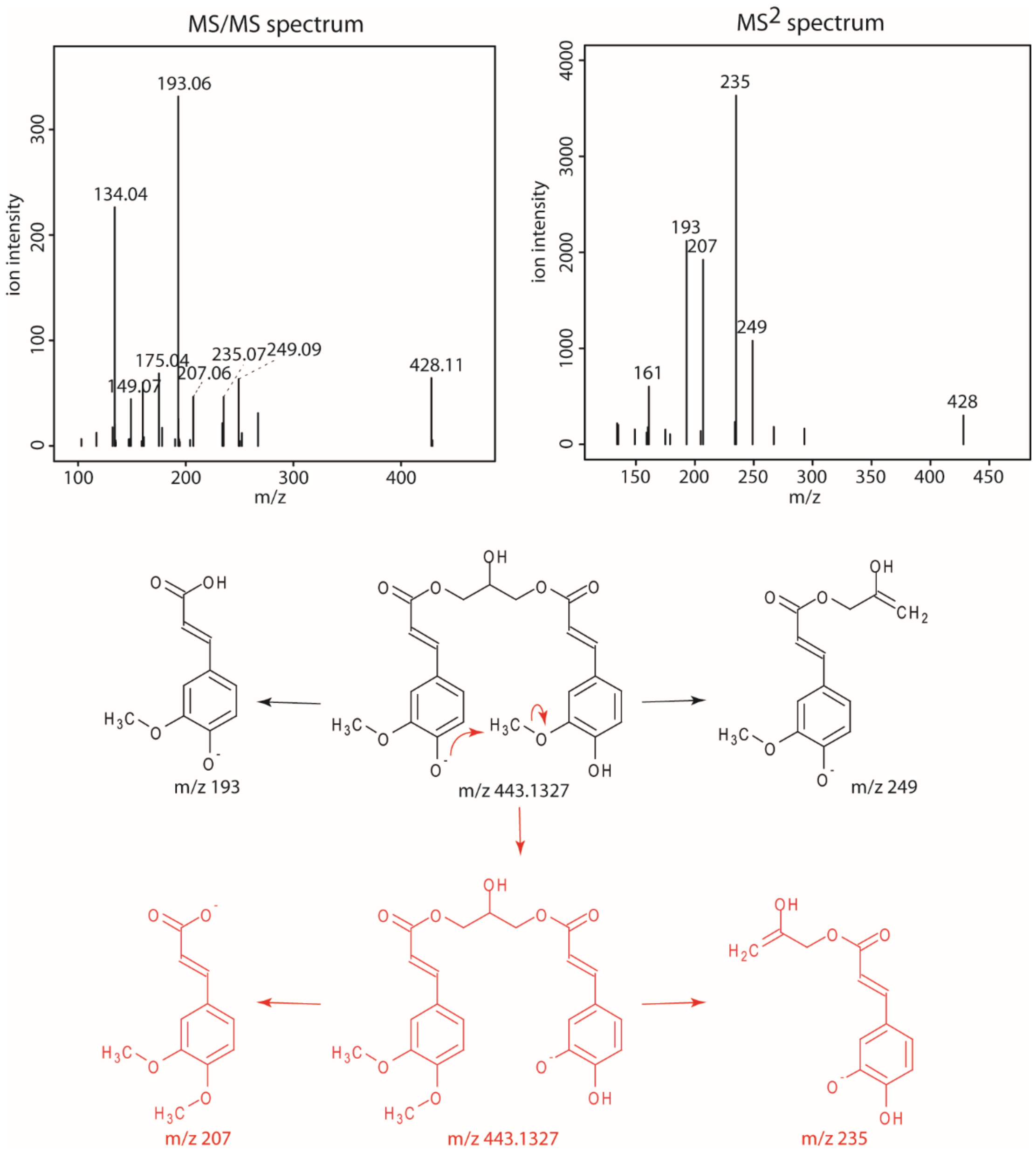

Supplemental Figure 15. CID Spectra and Tentative Gas-Phase Fragmentations of Diferuloyl Glycerol. Upon CID, the diferuloyl glycerol anion isomerizes to the dimethoxycinnamoyl caffeoyl glycerol anion; methyl transfer likely occurs via attack of a phenolate anion on the methoxy substituent of the other feruloyl moiety via a S_N2 reaction. Both isomers are then subjected to a charge-remote ester cleavage yielding ferulate (m/z 193) and a feruloyl hydroxyacetone ion (m/z 249), and dimethoxycinnamate (m/z 207) and a caffeoyl hydroxyacetone ion in the case of diferuloyl glycerol and dimethoxycinnamoyl caffeoyl glycerol, respectively. The ions at m/z 193 and 249 represent the complemental parts of diferuloyl glycerol (the sum of their neutral masses, i.e., 194 Da and 250 Da, equals the mass of diferuloyl glycerol, i.e., 444 Da). Similarly, the ions at m/z 207 and 235 are also complementary. These two pairs of complementary ions are immediately clear from the MS/MS spectrum owing to the m/z value accuracy of the product ions, yet more insight into their formation is gained from the MS^2 spectrum. As a result of the longer timeframe of CID reactions in an Ion Trap (IT)-MS as compared to those in a Quadrupole-Time-Of-Flight

(QTOF)-MS, fragmentations in the former MS analyzer are more thermodynamically controlled. Indeed, the rearrangement of the diferuloyl glycerol anion to dimethoxycinnamoyl caffeoyl glycerol anion occurs much less frequently in the QTOF-MS than in IT-MS, hence, fragmentations occur mainly from the diferuloyl glycerol anion in the QTOF-MS, explaining the high abundance of the m/z 193 ion in the MS/MS spectrum. In the case of IT-MS, as a result of the ortho-dihydroxybenzene moiety, the negative charge is better stabilized on dimethoxycinnamoyl caffeoyl glycerol than on diferuloyl glycerol. Consequently, fragmentations from the former anion will occur much more frequently. Indeed, the sum of the abundances of the m/z 207 and m/z 235 ions is larger than that of the m/z 193 and m/z 249 ions in the MS^2 spectrum. Furthermore, from the former pair of ions, the m/z 235 should be more abundant because the charge is well-stabilized on the ortho-dihydroxybenzene moiety and dimethoxycinnamate formation needs an additional proton transfer following ester cleavage. In the case of the latter pair of ions, the m/z 193 ion should be more stable, because the charge can reside on either the phenolic or the carboxylic acid function. These abundance differences between the four product ions are clearly visible in the MS^2 spectrum.

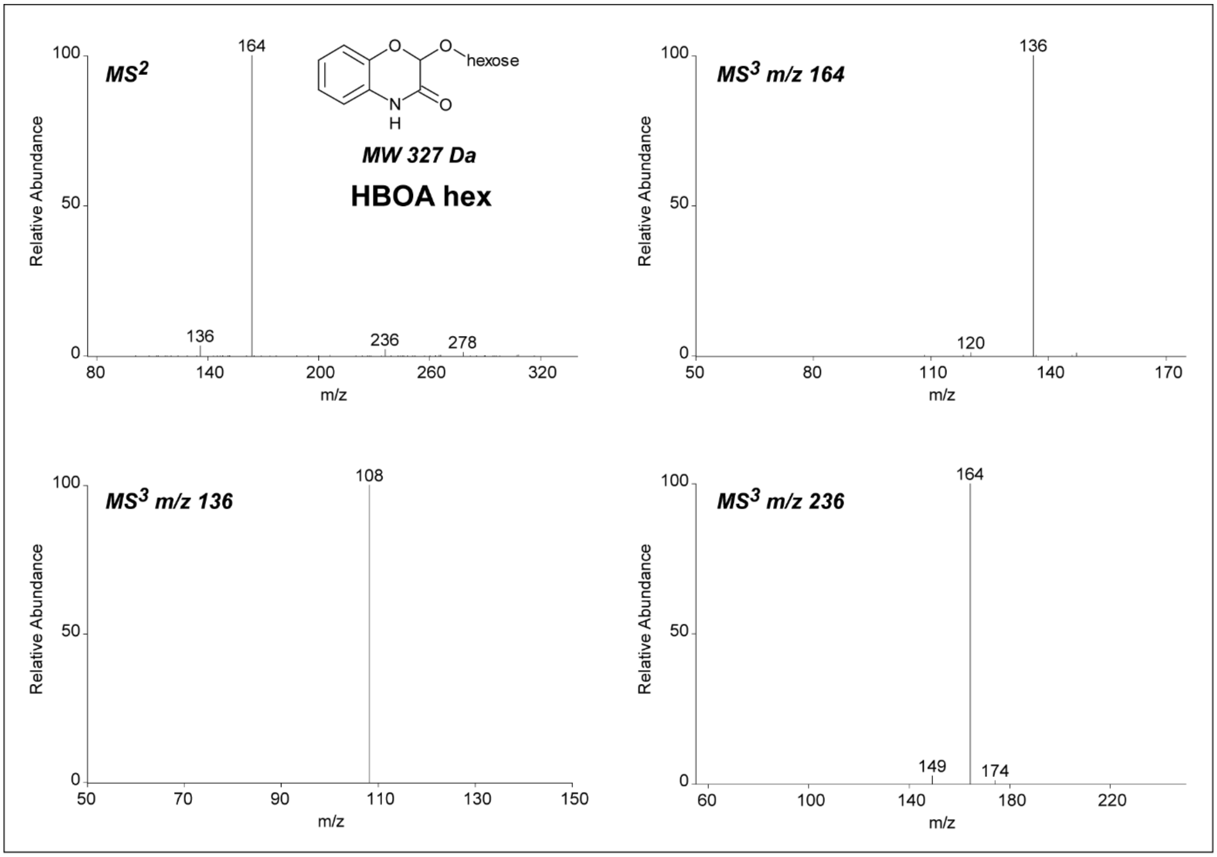
MS2
MW 327 Da
HBOA hex
hexose
Relative Abundance
m/z
164
136
236
278
MS3 m/z 164
136
120
MS3 m/z 136
108
MS3 m/z 236
164
149
174

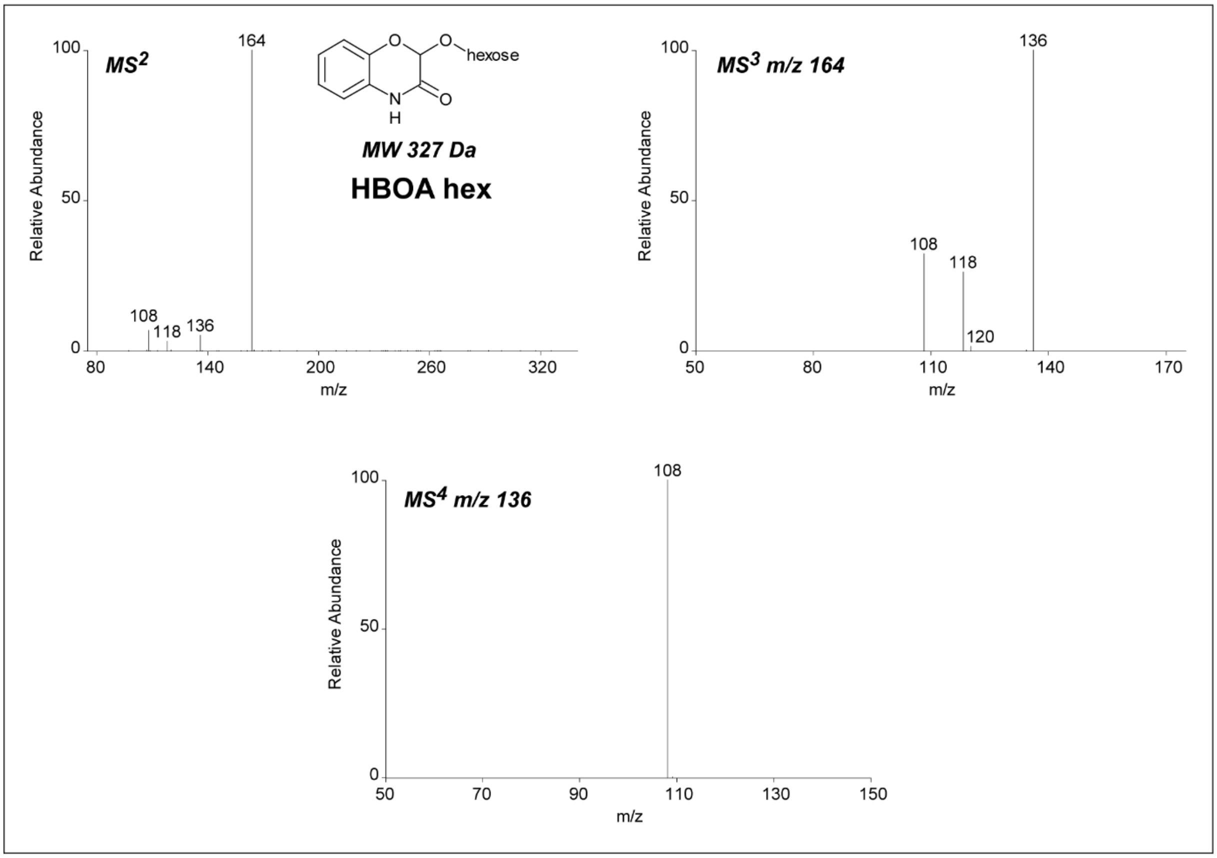
MS2
MW 327 Da
HBOA hex
hexose
Relative Abundance
m/z
164
108
118
136
MS3 m/z 164
136
108
118
120
MS4 m/z 136
108

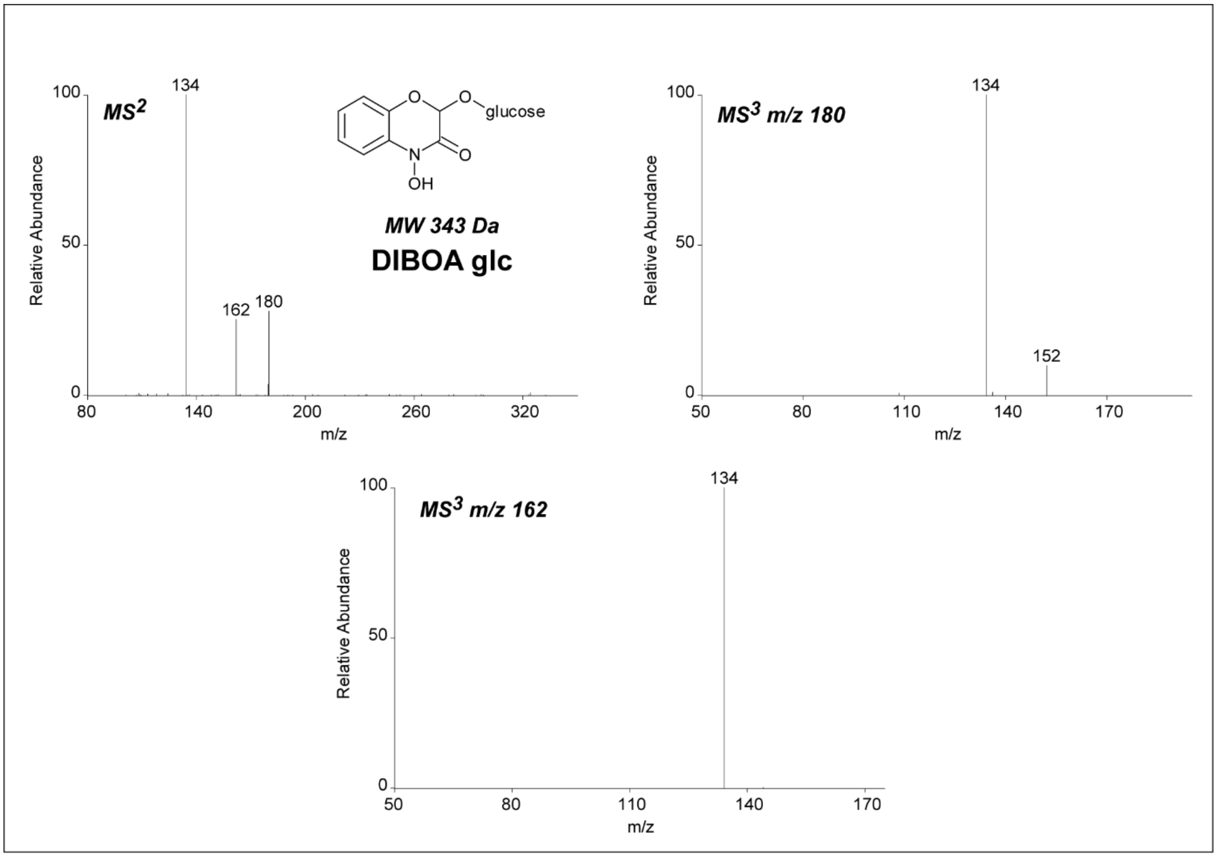
MS2
MW 343 Da
DIBOA glc
glucose
MS3 m/z 180
MS3 m/z 162
Relative Abundance
m/z
134
162
180
152

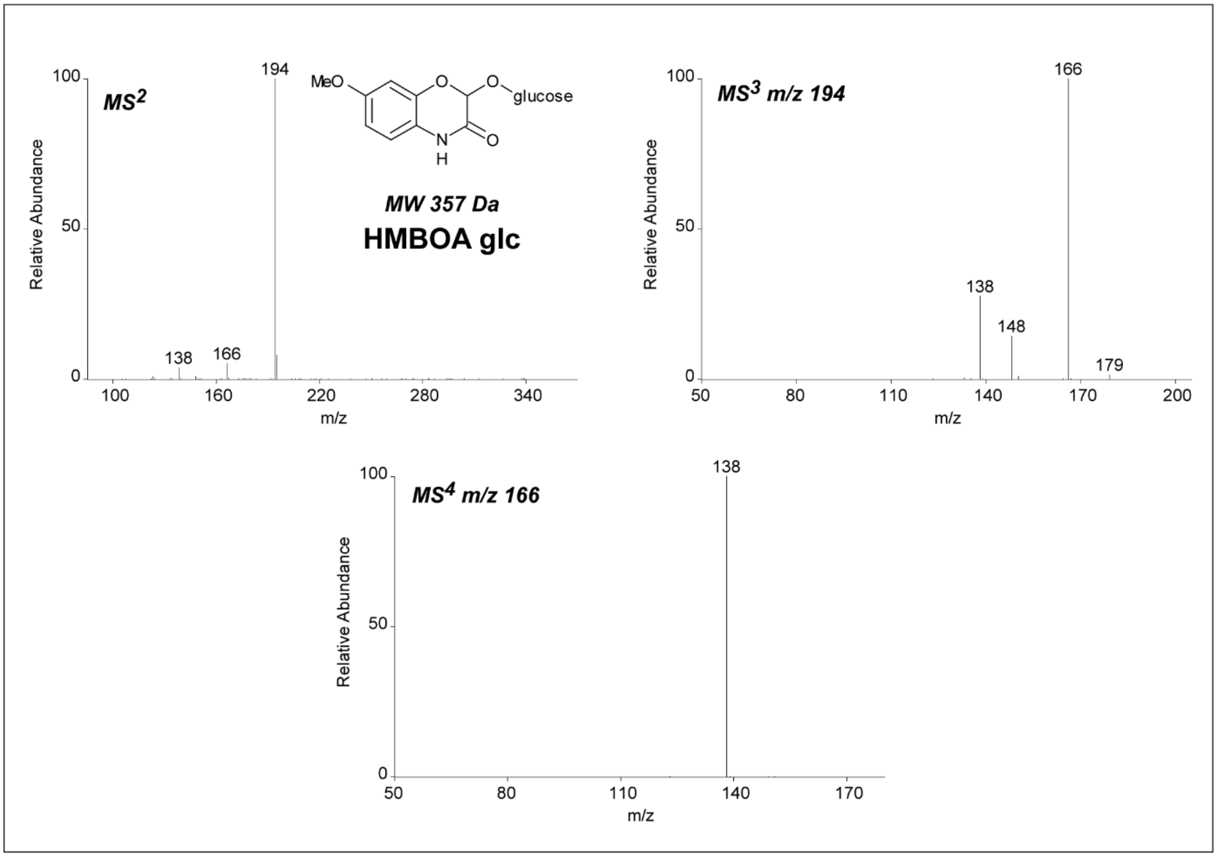
MS2
MW 357 Da
HMBOA glc
MeO
glucose
MS3 m/z 194
MS4 m/z 166
Relative Abundance
m/z
194
138
166
148
179

Supplemental Figure 16. Negative Ion MS^n Spectra of Some Benzoxazinoids.

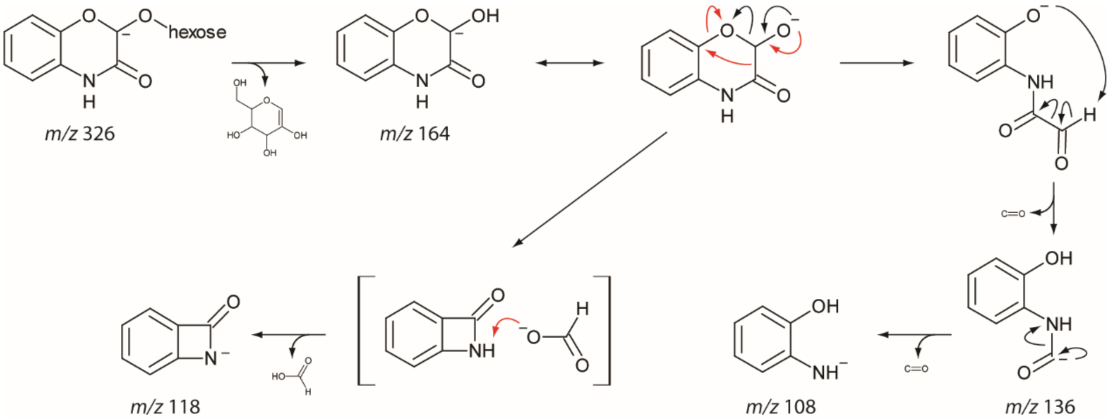

Supplemental Figure 17. Tentative Gas-Phase Fragmentation Pathway of the HBOA Glucoside Anion. The charge on HBOA glucoside is expected to mainly reside on the amide nitrogen and the carbonyl C_{α} position. See Supplemental Text for explanations.

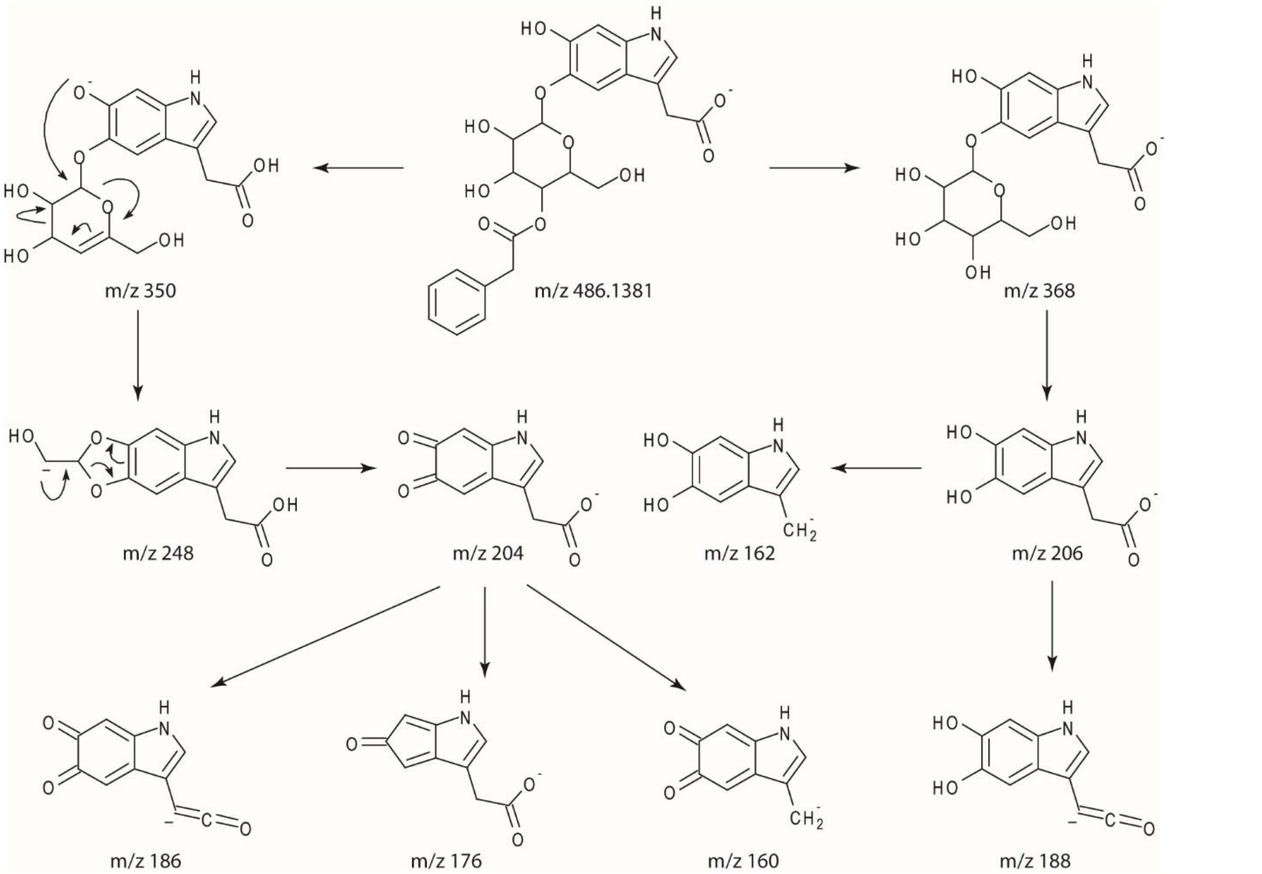

Supplemental Figure 18. Tentative Gas-Phase Fragmentation Pathway of the Anion from Dihydroxyindole-3-Acetic Acid (Phenylacetyl) Hexoside.

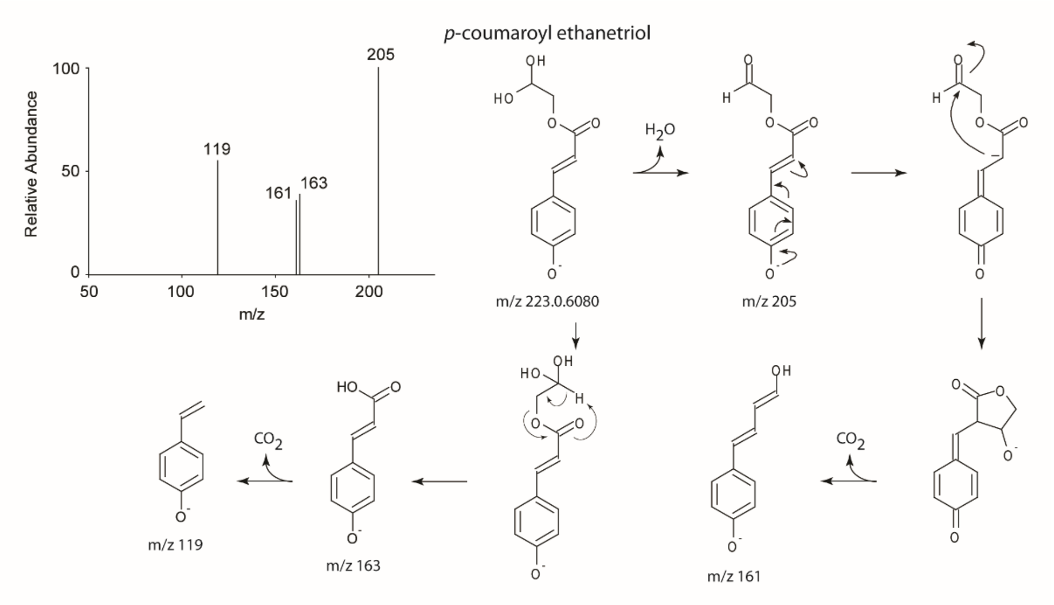

Supplemental Figure 19. Tentative Gas-Phase Fragmentation Pathway of *p*-Coumaroyl Ethanetriol. The proposed charge-remote ester cleavage via a 6-center intermediate with the formation of the m/z 163 carboxylate ion is similar to that occurring in acylglycerols (Stroobant et al., 1995). The m/z 161 ion is likely formed via a rearrangement: charge-remote water loss from the gem diol moiety results in an aldehyde. Subsequently, a charge-induced nucleophilic attack on the aldehyde yields a five-membered cyclic intermediate from which a decarboxylation leads to the m/z 161 ion.

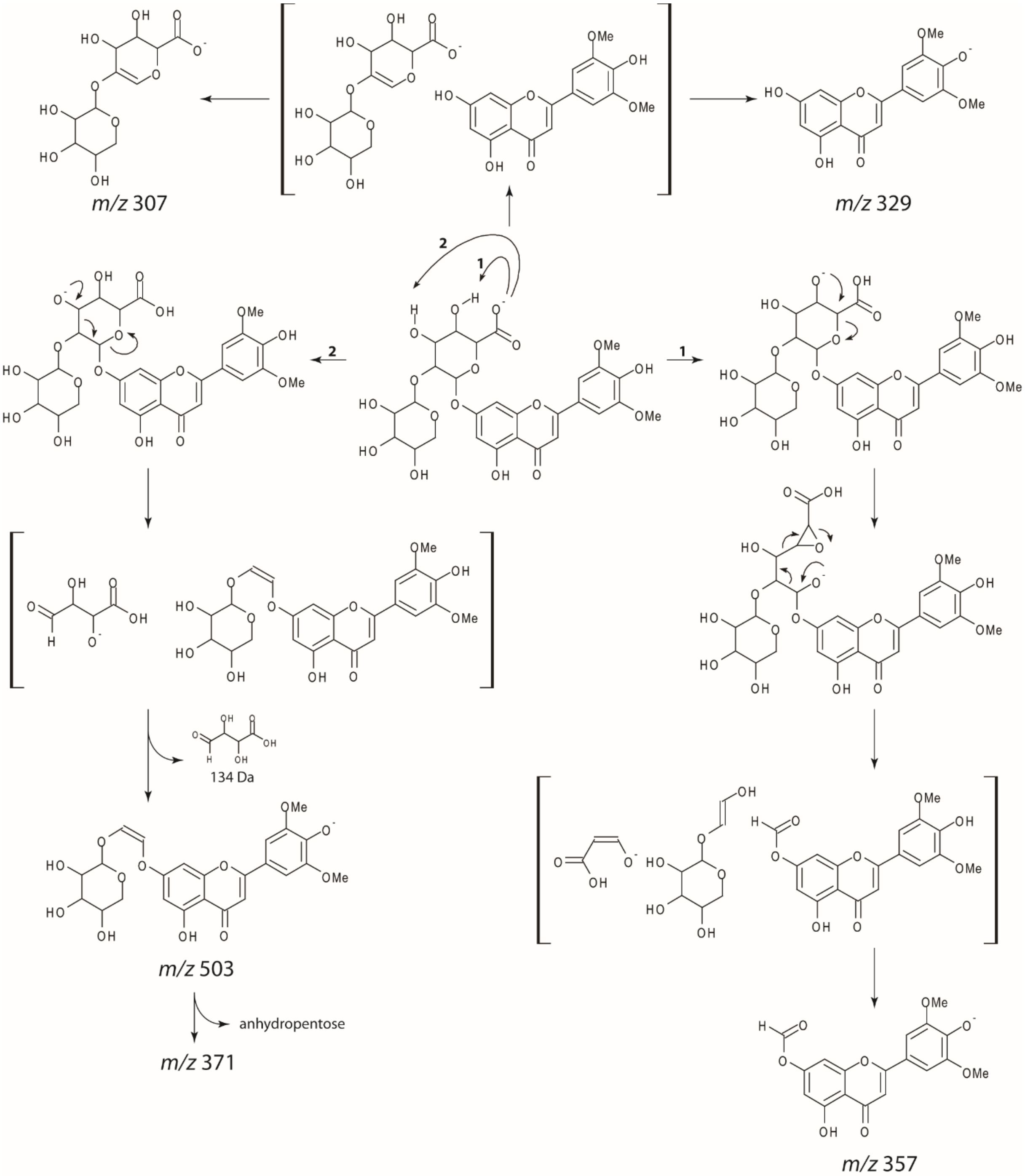

Supplemental Figure 20. Tentative Main Gas-Phase Fragmentation Pathways of Tricin-*O*-Pentosyl-(1→2)-Hexuronide.

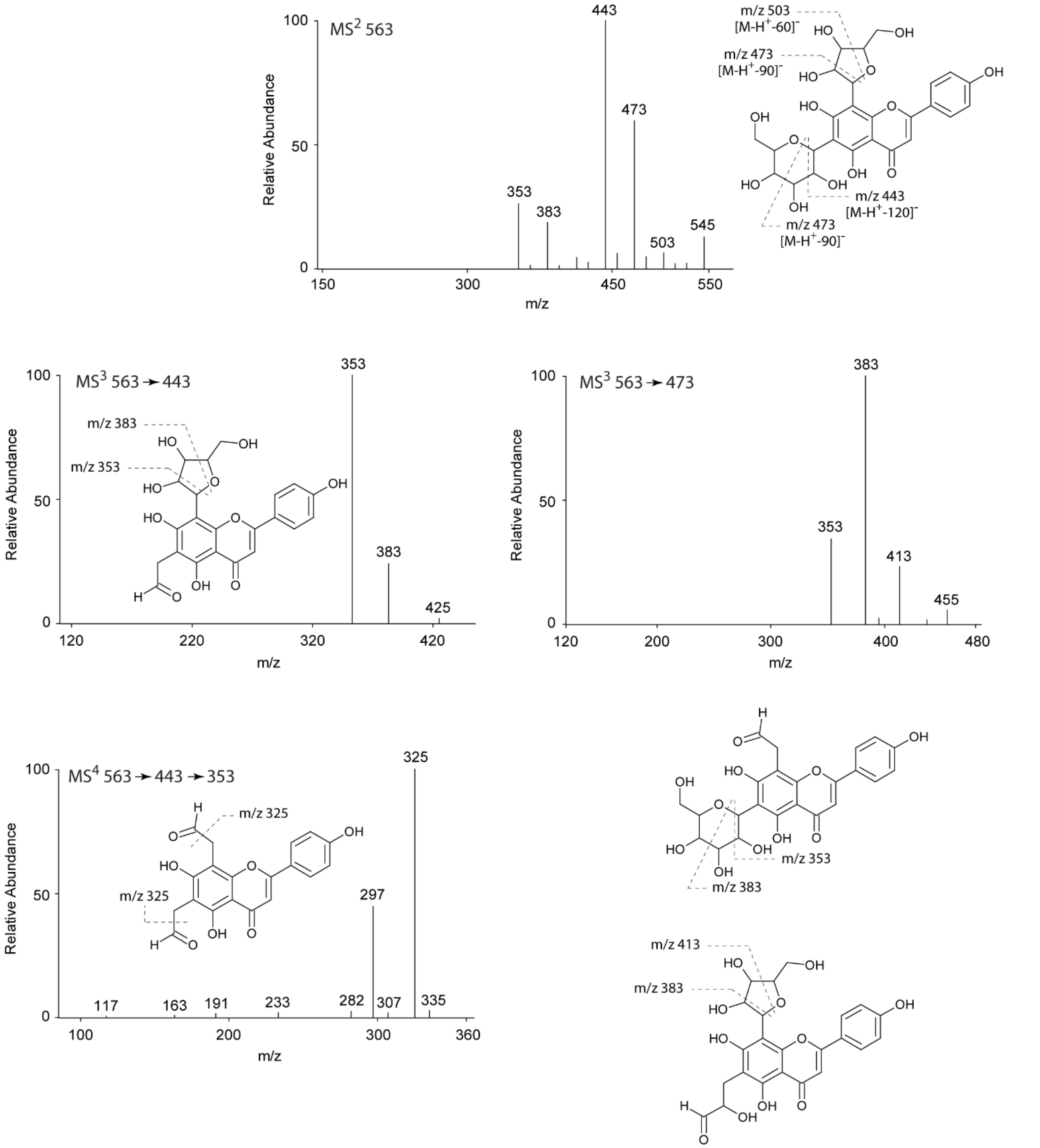

Supplemental Figure 21. MS^n Ion Tree and Gas-Phase Fragmentation Pathways of 6-*C*-Hexosyl-8-*C*-Pentosyl-Apigenin.

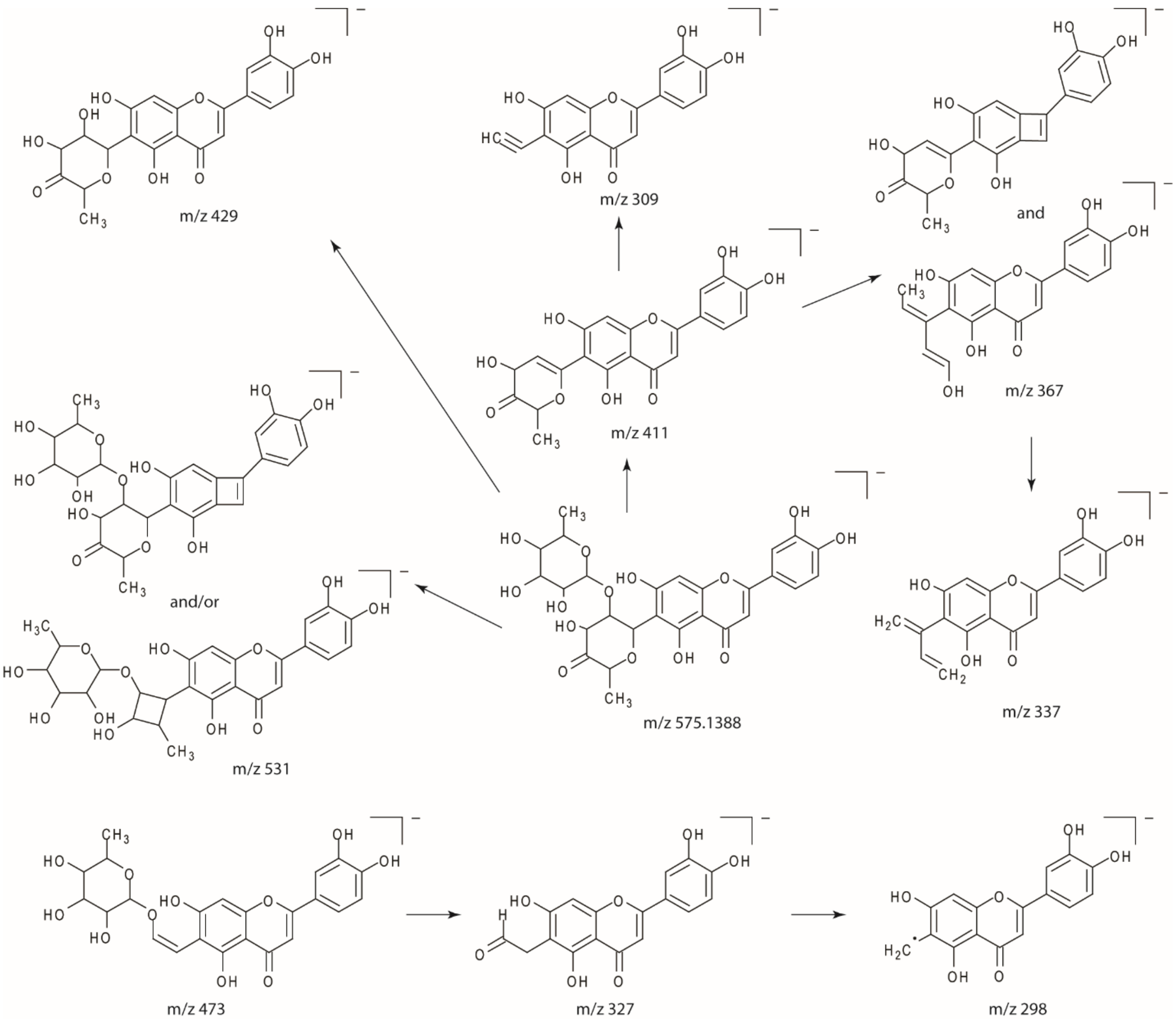

Supplemental Figure 22. Tentative Gas-Phase Fragmentations of the Maysin Anion upon CID. Multiple tautomers are present owing to the α-hydroxy-keto moiety.

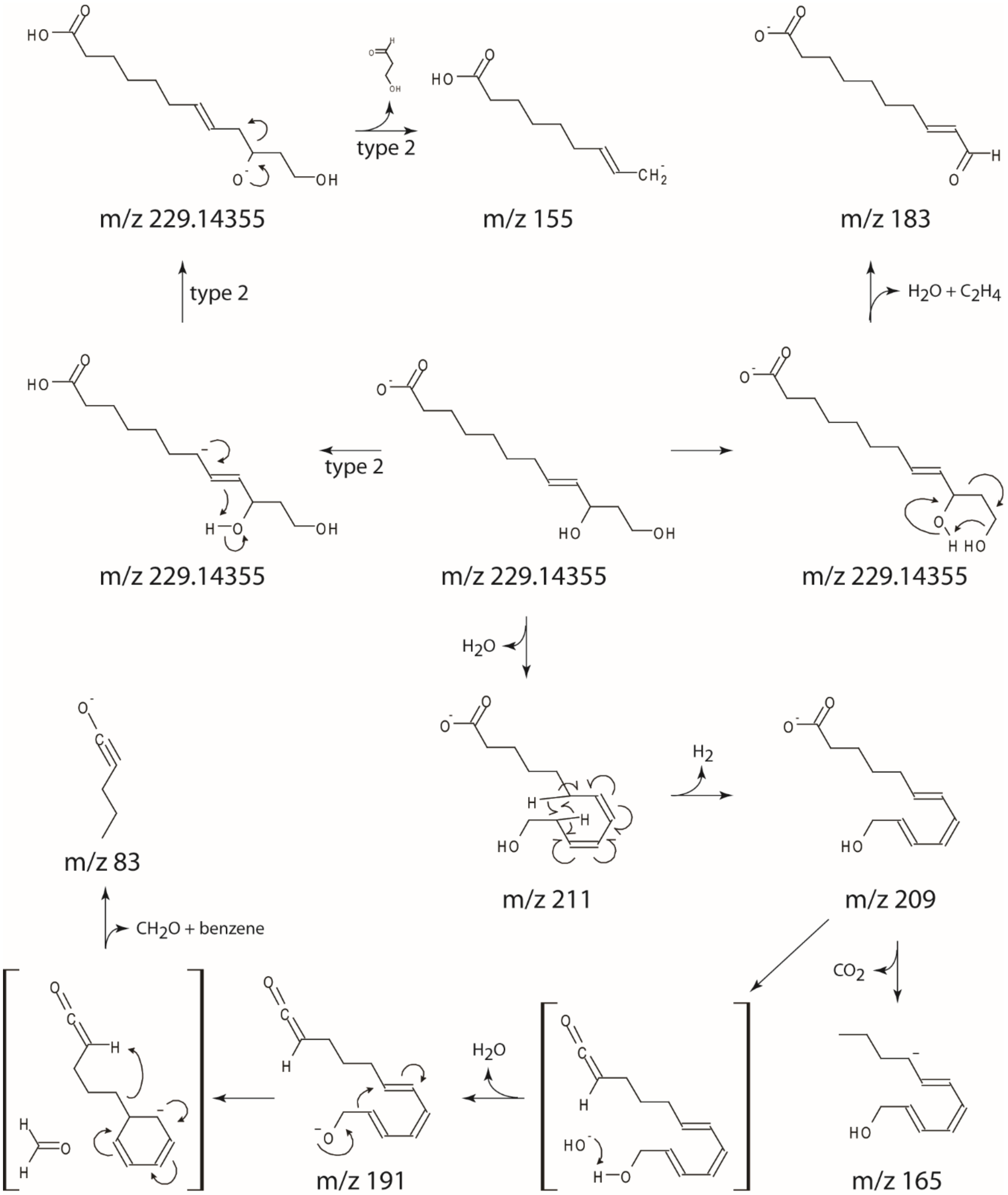

Supplemental Figure 23. Tentative Gas-Phase Fragmentations of the 10,12-Dihydroxy-9-Dodecenoic acid Anion upon CID.

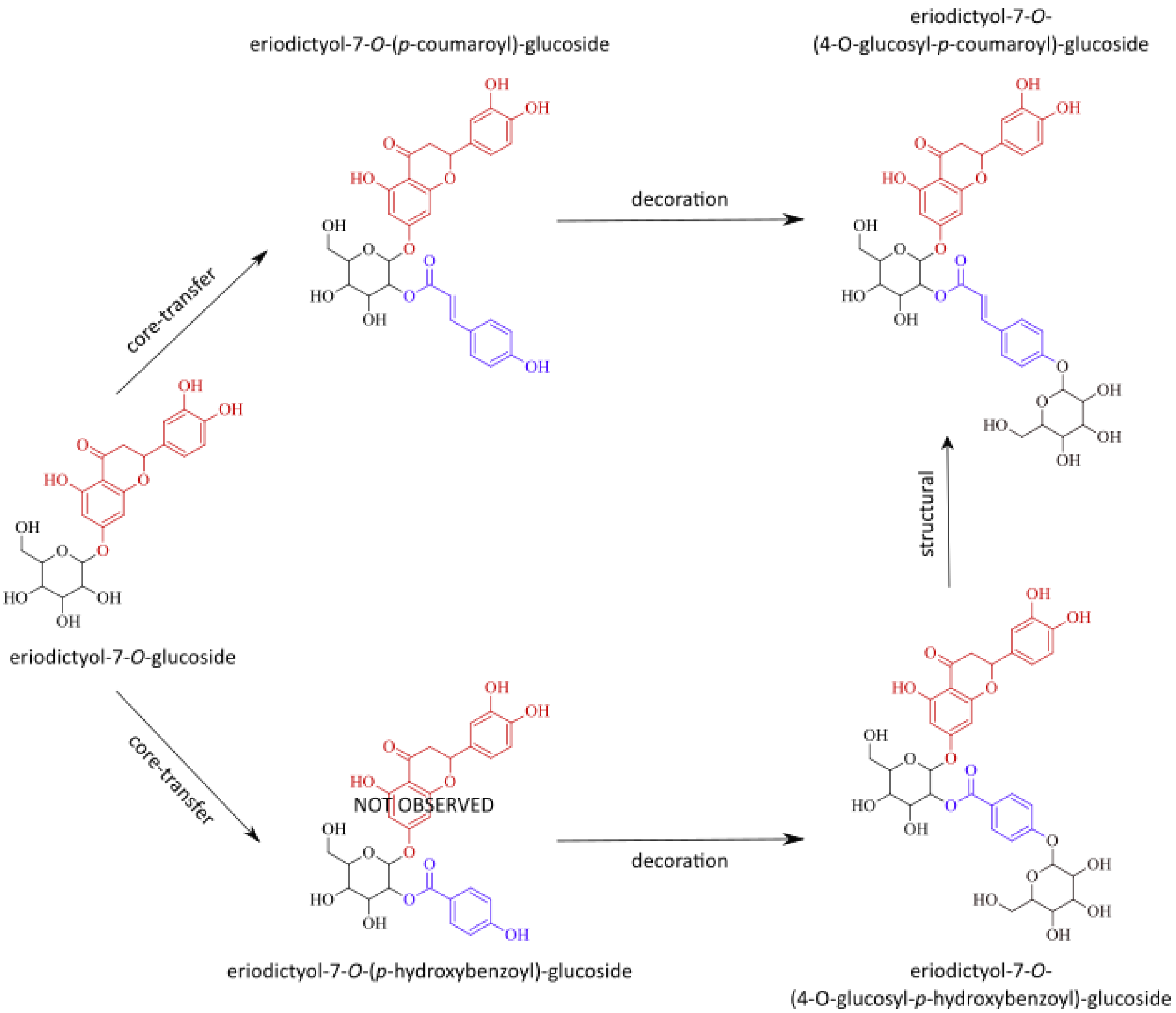

Supplemental Figure 24. Illustration of a local CSPP network with different biotransformation types.

SUPPLEMENTAL REFERENCES

Allen, F., Greiner, R., and Wishart, D. (2015) Competitive fragmentation modeling of ESI-MS/MS spectra for putative metabolite identification. Metabolomics **11**, 98-110

Bandu, M.L., Grubbs, T., Kater, M., and Desaire, H. (2006) Collision induced dissociation of alpha hydroxyl acids: evidence of an ion-neutral complex intermediate. Int. J. Mass Spectrom. **251**, 40-46

Bandu, M.L., Watkins, K.R., Bretthauer, M.L., Moore, C.A., and Desaire, H. (2004). Prediction of MS/MS Data. 1. A focus on pharmaceuticals containing carboxylic acids. Anal. Chem. **76:** 1746-1753.

Bonnington, L., Eljarrat, E., Guillamón, M., Eichhorn, P., Taberner, A., and Barceló, D. (2003) Development of liquid chromatography-electrospray-tandem mass spectrometry method for the quantitative determination of benzoxazinone derivatives in plants. Anal. Chem. **75**, 3128-3136

Cao, Y., Charisi, A., Cheng, L.-C., Jiang, T., and Girke, T. (2008). ChemmineR: a compound mining framework for R. Bioinformatics **24**, 1733–1734

Carroll, J.A., Willard, D., and Lebrilla, C.B. (1995) Energetics of cross-ring cleavages and their relevance to the linkage determination of oligosaccharides. Anal. Chim. Acta **307**, 431-447

Chiwocha, S.D.S., Abrams, S.R., Ambrose, S.J., Cutler, A.J., Loewen, M., Ross, A.R.S., and Kermode, A.R. (2003) A method for profiling classes of plant hormones and their metabolites using liquid chromatography-electrospray ionization tandem mass spectrometry: an analysis of hormone regulation of thermodormancy of lettuce (*Lactuca sativa* L.) seeds. Plant J. **35**, 405-417

Clifford, M.N., Johnston, K.L., Knight, S., and Kuhnert, N. (2003) Hierarchical scheme for LC-MS^n identification of chlorogenic acids. J. Agric. Food Chem. **51**, 2900-2911

Csárdi, G., and Nepusz, T. (2006). The igraph software package for complex network research. InterJournal, Complex Systems **1695,** 1-9

Custer, T.G., Kato, S., Fall, R., and Bierbaum, V.M. (2003) Negative ion CIMS: analysis of volatile leaf wound compounds including HCN. Int. J. Mass Spectrom. **223-224**, 427-446

Cuyckens, F., and Claeys, M. (2004) Mass spectrometry in the structural analysis of flavonoids. J. Mass Spectrom. **39**, 1-15

Da-Costa-Rocha, I., Bonnlaender, B., Sievers, H., Pischel, I., and Heinrich, M. (2014) *Hibiscus sabdariffa* L. – a phytochemical and pharmacological review. Food Chem. **165**, 424-443

Dauwe, R., Morreel, K., Goeminne, G., Gielen, B., Rohde, A., Van Beeumen, J., Ralph, J., Boudet, A.-M., Kopka, J., Rochange, S.F., Halpin, C., Messens, E., and Boerjan, W. (2007) Molecular phenotyping of lignin-modified tobacco reveals associated changes in cell-wall metabolism, primary metabolism, stress metabolism and photorespiration. Plant J. **52**, 263-285

Debrauwer, L., Paris, A., Rao, D., Fournier, F., and Tabet, J.-C. (1992) Mass spectrometric studies on 17β-estradiol-17-fatty acid esters: evidence for the formation of anion-dipole intermediates. Org. Mass Spectrom. **27**, 709-719

del Rio, J.C., Rencoret, J., Prinsen, P., Martínez, Á., Ralph, J., and Gutiérrez, A. (2012) Structural characterization of wheat straw lignin as revealed by analytical pyrolysis, 2D-NMR, and reductive cleavage methods. J. Agric. Food Chem. **60**, 5922-5935

Demarque, D.P., Crotti, A.E.M., Vessecchi, R., Lopes, J.L.C., and Lopes, N.P. (2016) Fragmentation reactions using electrospray ionization mass spectrometry: an important tool for the structural elucidation and characterization of synthetic and natural products. Nat. Prod. Rep. **33**, 432

Dima, O., Morreel, K., Vanholme, B., Kim, H., Ralph, J., and Boerjan, W. (2015) Small glycosylated lignin oligomers are stored in Arabidopsis leaf vacuoles. Plant Cell **27**, 695-710

Domon, B., and Costello, C.E. (1988) A systematic nomenclature for carbohydrate fragmentations in FAB-MS/MS spectra of glycoconjugates. Glycoconjugate J. **5**, 397-409

Dührkop, K., Shen, H., Meusel, M., Rousu, J., and Böcker, S. (2015) Searching molecular structure databases with tandem mass spectra using CSI:FingerID. Proc. Natl. Acad. Sci. U.S.A. **112**, 12580-12585

Eklund, P.C., Backman, M.J., Kronberg, L.Å., Smeds, A.I., and Sjöholm, R.E. (2008) Identification of lignans by liquid chromatography-electrospray ionization ion-trap mass spectrometry. J. Mass Spectrom. **43**, 97-107

Fabre, N., Rustan, I., de Hoffmann, E., and Quentin-Leclercq J. (2001) Determination of flavone, flavonol, and flavanone aglycones by negative ion liquid chromatography electrospray ion trap mass spectrometry. J. Am. Soc. Mass Spectrom. **12**, 707-715

Falcone Ferreyra, M.L., Rius, S.P., and Casati, P. (2012) Flavonoids: biosynthesis, biological functions, and biotechnological applications. Front. Plant Sci. **3**, 222

Ferreres, F., Silva, B.M., Andrade, P.B., Seabra, R.M., and Ferreira, M.A. (2003) Approach to the study of *C*-glycosyl flavones by ion trap HPLC-PAD-ESI/MS/MS: application to the seeds of Quince (*Cydonia oblonga*). Phytochem. Anal. **14**, 352-359

Ferreres, F., Llorach, R., and Gil-Izquierdo, A. (2004) Characterization of the interglycosidic linkage in di-, tri-, tetra- and pentaglycosylated flavonoids and differentiation of positional isomers by liquid chromatography/electrospray ionization mass spectrometry. J. Mass Spectrom. **39**, 312-321

Fournier, F., Remaud, B., Blasco, T., and Tabet, J.C. (1993) Ion-dipole complex formation from deprotonated phenol fatty acid esters evidenced by using gas-phase labeling combined with tandem mass spectrometry. J. Am. Soc. Mass Spectrom. **4**, 343-351

Fournier, F., Perlat, M.-C., and Tabet, J.-C. (1995) Control of internal proton transfers on ion-dipole complexes from $[M-H]^-$ ions of diphenol esters. Rapid Commun. Mass Spectrom. **9**, 13-17

Grossert, J.S., Fancy, P.D., and White, R.L. (2005) Fragmentation pathways of negative ions produced by electrospray ionization of acyclic dicarboxylic acids and derivatives. Can. J. Chem. **83**, 1878-1890

Grossert, J.S., Cook, M.C., and White, R.L. (2006) The influence of structural features on facile McLafferty-type, even-electron rearrangements in tandem mass spectra of carboxylate anions. Rapid Commun. Mass Spectrom. **20**, 1511-1516

Guha, R., and Cherto, M.R. (2017). rcdk: Integrating the CDK with R. https://mran.microsoft.com/snapshot/2018-04-25/web/packages/rcdk/vignettes/rcdk.pdf.

Hanhineva, K., Rogachev, I., Aura, A.-M., Aharoni, A., Poutanen, K., and Mykkänen, H. (2011) Qualitative characterization of benzoxazinoid derivatives in whole grain rye and wheat by LC-MS metabolite profiling. J. Agric. Food Chem. **59**, 921-927

Hughes, R.J., Croley, T.R., Metcalfe, C.D., and March, R.E. (2001) A tandem mass spectrometric study of selected characteristic flavonoids. Int. J. Mass Spectrom. **210-211**, 371-385

Hvattum, E., and Ekeberg, D. (2003) Study of the collision-induced radical cleavage of flavonoid glycosides using negative electrospray ionization tandem quadrupole mass spectrometry. J. Mass Spectrom. **38**, 43-46

Jena, B.S., Jayaprakasha, G.K., Singh, R.P., and Sakariah, K.K. (2002) Chemistry and biochemistry of (-)-hydroxycitric acid from *Garcinia*. J. Agric. Food Chem. **50**, 10-22

Jiang, N., Doseff, A.I., and Grotewold, E. (2016) Flavones: from biosynthesis to health benefits. Plants **5**, 27

Kanawati, B., and Schmitt-Kopplin, P. (2010). Exploring rearrangements along the fragmentation of glutaric acid negative ion: a combined experimental and theoretical study. Rapid Commun. Mass Spectrom. **24:** 1198-1206.

Kanawati, B., Herrmann, F., Joniec, S., Winterhalter, R., and Moortgat, G.K. (2008) Mass spectrometric characterization of *β*-caryophyllene ozonolysis products in the aerosol studied using an electrospray triple quadrupole and time-of-flight analyzer hybrid system and density functional theory. Rapid Commun. Mass Spectrom. **22**, 165-186

Kanawati, B., Joniec, S., Winterhalter, R., and Moortgat, G.K. (2008b) Mass spectrometric characterization of 4-oxopentanoic acid and gas-phase ion fragmentation mechanisms studied using a triple quadrupole and time-of-flight analyzer hybrid system and density functional theory. Rapid Commun. Mass Spectrom. **22**, 2269-2279

Kelland, M.A., Mady, M.F., and Lima-Eriksen, R. (2018) Kidney stone prevention: dynamic testing of edible calcium oxalate scale inhibitors. Cryst. Growth Des. **18**, 7441-7450

Kerwin, J.L., Wiens, A.M., and Ericsson, L.H. (1996) Identification of fatty acids by electrospray mass spectrometry and tandem mass spectrometry. J. Mass Spectrom. **31**, 184-192

Kerwin, J.L., and Torvik, J.J. (1996) Identification of monohydroxy fatty acids by electrospray mass spectrometry and tandem mass spectrometry. Anal. Biochem. **237**, 56-64

Kim, S., Thiessen, P.A., Bolton, E.E., Chen, J., Fu, G., Gindulyte, A., Han, L., He, J., He, S., Shoemaker, B.A., Wang, J., Yu, B., Zhang, J., and Bryant, S.H. (2016) PubChem Substance and Compound databases. Nucleic Acids Res. **44**, D1202-D1213

Kyada, A., Mansuri, N., and Patel, P. (2017) *In vitro* investigation of some alternative therapeutic agents for antiurolithiatic activity. J. Pharm. Res. **11**, 955-961

Lan, W., Lu, F., Regner, M., Zhu, Y., Rencoret, J., Ralph, S.A., Zakai, U.I., Morreel, K., Boerjan, W., and Ralph, J. (2015) Tricin, a flavonoid monomer in monocot lignification. Plant Physiol. **167**, 1284-1295

Lan, W., Morreel, K., Lu, F., Rencoret, J., del Río, J.C., Voorend, W., Vermerris, W., Boerjan, W., and Ralph, J. (2016) Maize tricin-oligolignol metabolites and their implications for monocot lignification. Plant Physiol. 171, 810-820

Lan, W., Rencoret, J., Lu, F., Karlen, S.D., Smith, B.G., Harris, P.J., del Rio, J.C., and Ralph, J. (2016b) Tricin-lignins: occurrence and quantitation of tricin in relation to phylogeny. Plant J. **88**, 1046-1057

Moe, M.K., Strøm, M.B., Jensen, E., and Claeys, M. (2004) Negative electrospray ionization low-energy tandem mass spectrometry of hydroxylated fatty acids: a mechanistic study. Rapid Commun. Mass Spectrom. **18**, 1731-1740

Morreel, K., Ralph, J., Kim, H., Lu, F., Goeminne, G., Ralph, S., Messens, E., and Boerjan, W. (2004a) Profiling of oligolignols reveals monolignol coupling conditions in lignifying poplar xylem. Plant Physiol. **136**, 3537-3549

Morreel, K., Ralph, J., Lu, F., Goeminne, G., Busson, R., Herdewijn, P., Goeman, J.L., Van der Eycken, J., Boerjan, W., and Messens, E. (2004b) Phenolic profiling of caffeic acid *O*-methyltransferase-deficient poplar reveals novel benzodioxane oligolignols. Plant Physiol. 136, 4023-4036

Morreel, K., Goeminne, G., Storme, V., Sterck, L., Ralph, J., Coppieters, W., Breyne, P., Steenackers, M., Georges, M., Messens, E., and Boerjan, W. (2006) Genetical metabolomics of flavonoid biosynthesis in *Populus*: a case study. Plant J. **47**, 224-237

Morreel, K., Kim, H., Lu, F., Dima, O., Akiyama, T., Vanholme, R., Niculaes, C., Goeminne, G., Inzé, D., Messens, E., Ralph, J., and Boerjan, W. (2010a) Mass spectrometry-based fragmentation as an identification tool in lignomics. Anal. Chem. **82**, 8095-8105

Morreel, K., Dima, O., Kim, H., Lu, F., Niculaes, C., Vanholme, R., Dauwe, R., Goeminne, G., Inzé, D., Messens, E., Ralph, J., and Boerjan, W. (2010b) Mass spectrometry-based sequencing of lignin oligomers. Plant Physiol. **153**, 1464-1478

Morreel, K., Saeys, Y., Dima, O., Lu, F., Van de Peer, Y., Vanholme, R., Ralph, J., Vanholme, B., and Boerjan, W. (2014) Systematic structural characterization of metabolites in *Arabidopsis* via candidate substrate-product pair networks. Plant Cell **26**, 929-945

Mulroney, B., Peel, B., and Traeger, J.C. (1999) Theoretical study of deprotonated glucopyranosyl disaccharide fragmentation. J. Mass Spectrom. **34**, 856-871

Nakamura, Y., Afendi, F.M., Parvin, A.K., Ono, N., Tanaka, K., Morita, A.H., Sato, T., Sugiura, T., Altaf-Ul-Amin, Md., and Kanaya, S. (2014) KNApSAcK metabolite activity database for retrieving relationships between metabolites and biological activities. Plant Cell Physiol. **55**, e7(1-9)

Neuwirth, E. (2014). RColorBrewer: ColorBrewer palettes. R package version 1.1-2. https://cran.r-project.org/web/packages/RColorBrewer/index.html.

Niculaes, C., Morreel, K., Kim, H., Lu, F., McKee, L.S., Ivens, B., Haustraete, J., Vanholme, B., De Rycke, R., Hertzberg, M., Fromm, J., Bulone, V., Polle, A., Ralph, J., and Boerjan, W. (2014) Phenylcoumaran benzylic ether reductase prevents accumulation of compounds formed under oxidative conditions in poplar xylem. Plant Cell **26**, 3775-3791

Niculaes, C., Abramov, A., Hannemann, L., and Frey, M. (2018) Plant protection by benzoxazinoids – recent insigths into biosynthesis and function. Agronomy **8**, 143

Rao, R.N., and Sakariah, K.K. (1988) Lipid-lowering and anti-obesity effect of (-)-hydroxycitric acid. Nutr. Res. 8, 209-212

Rasche, F., Svatoš, A., Maddula, R.K., Böttcher, C., and Böcker, S. (2011) Computing fragmentation trees from tandem mass spectrometry data. Anal. Chem. **83**, 1243–1251

R Core Team (2017) R: A language and environment for statistical computing. Foundation for Statistical Computing, Vienna, Austria. URL https://www.R-project.org/.

Revelou, P.-K., Kokotou, M.G., and Constantinou-Kokotou, V. (2019) Identification of auxin metabolites in *Brassicaceae* by ultra-performance liquid chromatography coupled with high-resolution mass spectrometry. Molecules **24**, 2615

Rojas-Cherto, M., Peironcely, J.E., Kasper, P.T., van der Hooft, J.J.J., de Vos, R.C.H., Vreeken, R., Hankemeier, T., and Reijmers, T. (2012). Metabolite identification using automated comparison of high-resolution multistage mass spectral trees. Anal. Chem. **84:** 5524-5534.

Schnable, P.S., Ware, D., Fulton, R.S., Stein, J.C., Wei, F., et al. (2009) The B73 maize genome: complexity, diversity, and dynamics. Science **326**, 1112-1115

Seethapathy, G.S., Tadesse, M., Urumarudappa, S.K.J., Gunaga, S.V., Vasudeva, R., Malterud, K.E., Shaanker, R.U., de Boer, H.J., Ravikanth, G., and Wangensteen, H. (2018) Authentication of *Garcinia* fruits and food supplements using DNA barcoding and NMR spectroscopy. Sci. Rep. **8**, 10561

Smith, C.A., Want, E.J., O'Maille, G., Abagyan, R., and Siuzdak, G. (2006) XCMS: processing mass spectrometry data for metabolite profiling using nonlinear peak alignment, matching and identification. Anal. Chem. **78**, 779-787

Stroobant, V., Rozenberg, R., Bouabsa, e. M., Deffense, E., and de Hoffmann, E. (1995) Fragmentation of conjugate bases of esters derived from multifunctional alcohols including triacylglycerols. J. Am. Soc. Mass Spectrom. **6**, 498-506

Tsuji, Y., Vanholme, R., Tobimatsu, Y., Ishikawa, Y., Foster, C.E., Kamimura, N., Hishiyama, S., Hashimoto, S., Shino, A., Hara, H., Sato-Izawa, K., Oyarce, P., Goeminne, G., Morreel, K., Kikuchi, J., Takano, T., Fukuda, M., Katayama, Y., Boerjan, W., Ralph, J., Masai, E., and Kajita, S. (2015) Introduction of chemically labile substructures into *Arabidopsis* lignin through the use of LigD, the Cα-dehydrogenase from *Sphingobium* sp. strain SYK-6. Plant Biotechnol. J. 13, 821-832

van der Hooft, J.J.J., Wandy, J., Barrett, M.P., Burgess, K.E.V., and Rogers, S. (2016) Topic modeling for untargeted substructure exploration in metabolomics. **113**, 13738-13743

Vanholme, R., Ralph, J., Akiyama, T., Lu, F., Rencoret Pazo, J., Kim, H., Christensen, J.H., Van Reusel, B., Storme, V., De Rycke, R., Rohde, A., Morreel, K., and Boerjan, W. (2010) Engineering traditional monolignols out of lignin by concomitant up-regulation of *F5H1* and down-regulation of *COMT* in Arabidopsis. Plant J. **64**, 885-897

Walker, V., Bertrand, C., Bellvert, F., Moënne-Loccoz, Y., Bally R., and Comte G. (2011) Host plant secondary metabolite profiling shows a complex, strain-dependent response of maize to plant growth-promoting rhizobacteria of the genus *Azospirillum*. New Phytol. 189: 494-506

Watrous, J., Roach, P., Alexandrov, T., Heath, B.S., Yang, J.Y., Kersten, R.D., van der Voort, M., Pogliano, K., Gross, H., Raaijmakers, J.M., Moore, B.S., Laskin, J., Bandeira, N., and Dorrestein, P.C. (2012) Mass

spectral molecular networking of living microbial colonies. Proc. Natl. Acad. Sci. U.S.A. **109**, E1743–E1752

Wheelan, P., Zirrolli, J.A., and Murphy, R.C. (1993) Low-energy fast atom bombardment tandem mass spectrometry of monohydroxy substituted unsaturated fatty acids. Biologic. Mass Spectrom. **22**, 465-473

Wheelan, P., Zirrolli, J.A., and Murphy, R.C. (1996) Negative ion electrospray tandem mass spectrometric structural characterization of leukotriene B_4 (LTB_4) and LTB_4-derived metabolites. J. Am. Soc. Mass Spectrom. **7**, 129-139

Wheelan, P., Zirrolli, J.A., and Murphy, R.C. (1996b) Electrospray ionization and low energy tandem mass spectrometry of polyhydroxy unsaturated fatty acids. J. Am. Soc. Mass Spectrom. **7**, 140-149

Wojakowska, A., Perkowski, J., Góral, T., and Stobiecki, M. (2013) Structural characterization of flavonoid glycosides from leaves of wheat (*Triticum aestivum* L.) using LC/MS/MS profiling of the target compounds. J. Mass Spectrom. **48**, 329-339

Wolf, S., Schmidt, S., Müller-Hannemann, M., and Neumann, S. (2010) In silico fragmentation for computer assisted identification of metabolite mass spectra. BMC Bioinformatics **11**, 148

Wouters, F.C., Reichelt, M., Glauser, G., Bauer, E., Erb, M., Gershenzon, J., and Vassão, D.G. (2014) Reglucosylation of the benzoxazinoid DIMBOA with inversion of stereochemical configuration is a detoxification strategy in lepidopteran herbivores. Angew. Chem. Int. Ed. **53**, 11320-11324

Wouters, F.C., Gershenzon, J., and Vassão, D.G. (2016) Benzoxazinoids: reactivity and modes of action of a versatile class of plant chemical defenses. J. Braz. Chem. Soc. **27**, 1379-1397

Xu, C., Ren, Y., Jian, Y., Guo, Z., Zhang, Y., Xie, C., Fu, J., Wang, H., Wang, G., Xu, Y., Li, P., and Zou, C. (2017) Development of a maize 55 K SNP array with improved genome coverage for molecular breeding. Mol. Breeding **37**, 20

Yan, C., Liu, S., Zhou, Y., Song, F., Cui, M., and Liu, Z. (2007) A study of isomeric diglycosyl flavonoids by SORI CID of Fourier transform ion cyclotron mass spectrometry in negative ion mode. J. Am. Soc. Mass Spectrom. **18**, 2127-2136

Ye, H., Zhu, L., Sun, D., Luo, X., Lu, G., Wang, H., Wang, J., Cao, C., Xiao, W., Wang, Z., Wang, G., and Hao, H. (2016) Nontargeted diagnostic ion network analysis (NINA): a software to streamline the analytical workflow for untargeted characterization of natural medicines. J. Pharm. Biomed. Anal. **131**, 40-47

Zhao, H., Jiang, M., Liang, Q., Xie, C., Song, S., Wang, J., Bai, G., and Luo, G. (2013) Fragmentation pathway studies of several plant hormones using an electrospray ionization-quadrupole/time-of-flight mass spectrometer. Int. J. Mass Spectrom. **335**, 7-15

Zhou, S., Richter, A., and Jander, G. (2018) Beyond defense: multiple functions of benzoxazinoids in maize metabolism. Plant Cell Physiol. **59**, 1528-1537
